# Climate deteriorations and Neanderthal demise in interior Iberia

**DOI:** 10.1038/s41598-018-25343-6

**Published:** 2018-05-04

**Authors:** D. Wolf, T. Kolb, M. Alcaraz-Castaño, S. Heinrich, P. Baumgart, R. Calvo, J. Sánchez, K. Ryborz, I. Schäfer, M. Bliedtner, R. Zech, L. Zöller, D. Faust

**Affiliations:** 1 Institute of Geography, Technische Universität Dresden, Helmholtzstr. 10, D-01069 Dresden, Germany; 2 Institute of Geography, Universität Bayreuth, Universitätsstr. 30, D-95440 Bayreuth, Germany; 3 Área de Prehistoria, Universidad de Alcalá, Calle Colegios 2, 28801 Alcalá de Henares, Madrid, Spain; 4 Neanderthal Museum, Talstraße 300, 40822 Mettmann, Germany; 5 Departamento de Ingeniería Civil y de la Edificación, E.T.S.I. Caminos, Canales y Puertos, Universidad de Castilla La Mancha, Ciudad Real, Spain; 6 Institute of Geography and Oeschger Centre for Climate Change Research, University of Bern, Hallerstr. 12, CH-3012 Bern, Switzerland; 7 Institute of Geography, Friedrich-Schiller-Universität Jena, Löbdergraben 32, D-07743 Jena, Germany

## Abstract

Time and circumstances for the disappearance of Neanderthals and its relationship with the advent of Modern Humans are not yet sufficiently resolved, especially in case of the Iberian Peninsula. Reconstructing palaeoenvironmental conditions during the last glacial period is crucial to clarifying whether climate deteriorations or competition and contacts with Modern Humans played the pivotal role in driving Neanderthals to extinction. A high-resolution loess record from the Upper Tagus Basin in central Spain demonstrates that the Neanderthal abandonment of inner Iberian territories 42 kyr ago coincided with the evolvement of hostile environmental conditions, while archaeological evidence testifies that this desertion took place regardless of modern humans’ activities. According to stratigraphic findings and stable isotope analyses, this period corresponded to the driest environmental conditions of the last glacial apart from an even drier period linked to Heinrich Stadial 3. Our results show that during Marine Isotope Stages (MIS) 4 and 2 climate deteriorations in interior Iberia temporally coincided with northern hemisphere cold periods (Heinrich stadials). Solely during the middle MIS 3, in a period surrounding 42 kyr ago, this relation seems not straightforward, which may demonstrate the complexity of terrestrial climate conditions during glacial periods.

## Introduction

It is in the very nature of major cultural shifts to attract considerable interest and provoke lively debate, in particular when that topic is at the intersection of natural and anthropological sciences^1–3^. Against this backdrop, the disappearance of Neanderthals and the arrival and spread of anatomically modern humans (AMH) in Eurasia, roughly coincident with the Middle to Upper Palaeolithic transition (MUPT), presents one of the most momentous transitions of cultural periods in human history^4^, although concomitant circumstances have not yet been fully explained^5–8^. In this context, the Iberian Peninsula occupies a particular position, since there is controversy on whether the MUPT proceeded spatially and temporally variable, which would enable a potential overlapping of several millennia between Middle and Upper Palaeolithic cultures^9–11^. One of the prima facie contrasting implications of such overlapping may be that Neanderthals were driven to extinction by competition with AMHs^5,12^, and^13^ or by assimilation into their genetic pool^7,10^. Another widely held view is, however, that the Neanderthals gradually disappeared due to a substantial deterioration of environmental conditions^14^.

Unfortunately, the absence of suitable environmental archives within the territories settled by hominines in Iberia seriously hampered to evaluate the significance of last glacial climate and environmental changes for cultural transitions and population movements. Instead, information from deep-sea drilling are frequently used in order to reconstruct and simulate palaeoenvironmental conditions across the Iberian Peninsula^15–17^. However, it is not yet fully clear whether these results are indeed transferrable to inner continental regions^18,19^.

**Figure 1:**
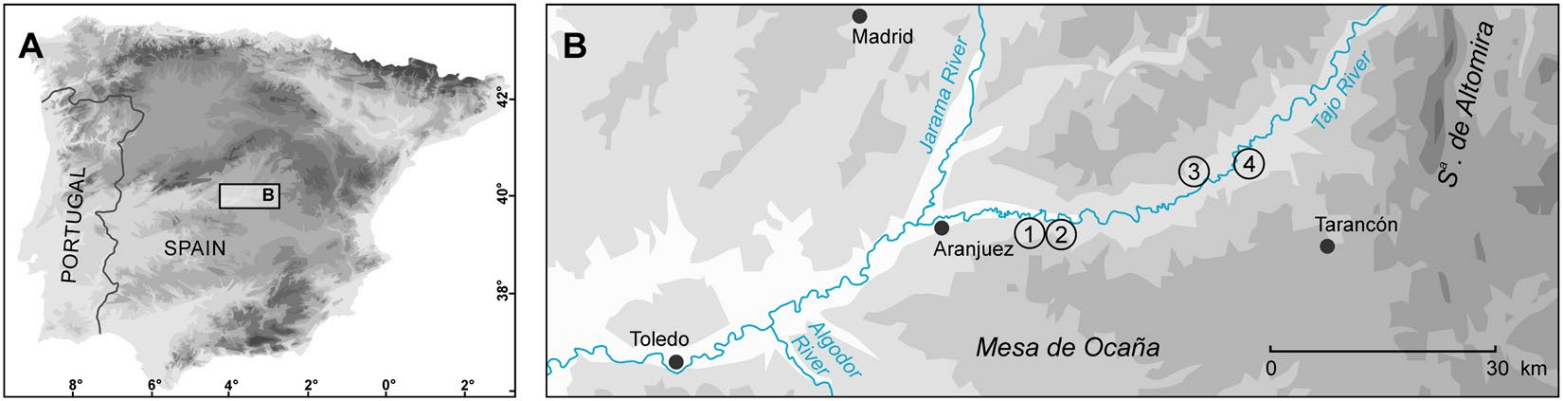
Maps showing (**A**) the location of the Tagus loess record in central Spain and (**B**) the position of the loess sections mentioned in the text: 1 Section Paraíso; 2 Section Villarubia; 3 Section Fuentidueña; 4 Section A3. The map was generated using ArcGIS 10.2.2 (http://www.esri.com/software/arcgis).

The final demise of Neanderthal populations is generally attributed to a dramatic drying and cooling of climatic conditions in the context of the Heinrich event 4 (H4)^20,21^ that has been recorded in a number of deep-sea drillings off the western Iberian margin and in the Western Mediterranean Sea. However, recent studies provide evidence that the disappearance of the Middle Palaeolithic precedes the onset of H4 in many places^11,22–24^. This questions the importance of climate for cultural dynamics, or alternatively the significance of marine records for environmental conditions in inland areas. To explore a terrestrial sedimentary archive that provides climate-related information independent from deep-sea drillings for the last glacial period, we investigated loess-palaeosoil sequences from the upper Tagus Basin (Fig. 1) and determined the stratigraphical and chronological framework of loess deposition as an indication of harsh environmental conditions. This paper examines the timing and duration of inner Iberian periods of climate deterioration and reviews whether these periods comply with regional Palaeolithic settlement patterns and ultimately with Heinrich events as detected in marine environments.

## Results

### Nature and origin of loess deposits in interior Iberia

The loess cover in the upper Tagus Basin close by the city of Aranjuez is broken down into small areas with a few hectares in size that generally follow wind-sheltered positions provided by the relief. These positions are mainly distributed on both sides of the Tagus River that incised up to 200 meters deep into the southern Meseta, a Pliocene erosional surface that exposes vast areas of Tertiary evaporitic marls (Fig. S1). Up to eight meters of primary loess but also relocated loess-like material rest either on strongly dissected valley flanks set up in evaporites, or on middle to late Pleistocene gravel terraces that make up about 40 percent of the valley floor and rise about 18 to 20 meters above recent river level.

The character of the loess deposits clearly demonstrates a local to regional origin of sediments and thus allows conclusions on palaeoenvironmental conditions representative for central Iberia. In agreement with reference samples from evaporites, gypsum contents of more than 15 percent in some loess units (SI Texts 1–4) bear witness on the incorporation of marl material into the loess deposits. Furthermore, high carbonate contents and a mineral spectrum rich in dolomite and tourmaline suggest that fluvial sediments from the Tagus River should be a major source of aeolian deposits (SI Text 8, Figs S17 and S18). Along with lacking evidence of significant remote components, this leads us to the conclusion that loess formation not just indicates higher wind speeds, but also regional environmental conditions that resulted in the mobilization and supply of deflatable fine material, especially from the Iberian Range and Sierra de Altomira in the headwaters of the Tagus River. The latter involves increased sediment production by cryoclastic, or even periglacial and glacial weathering processes^25^, as well as intensive fluvial transport and floodplain deposition. This drives us to relate dynamics of loess formation primarily with cold and dry climate conditions.

### Stratigraphical sequence and age determination

**Figure 2:**
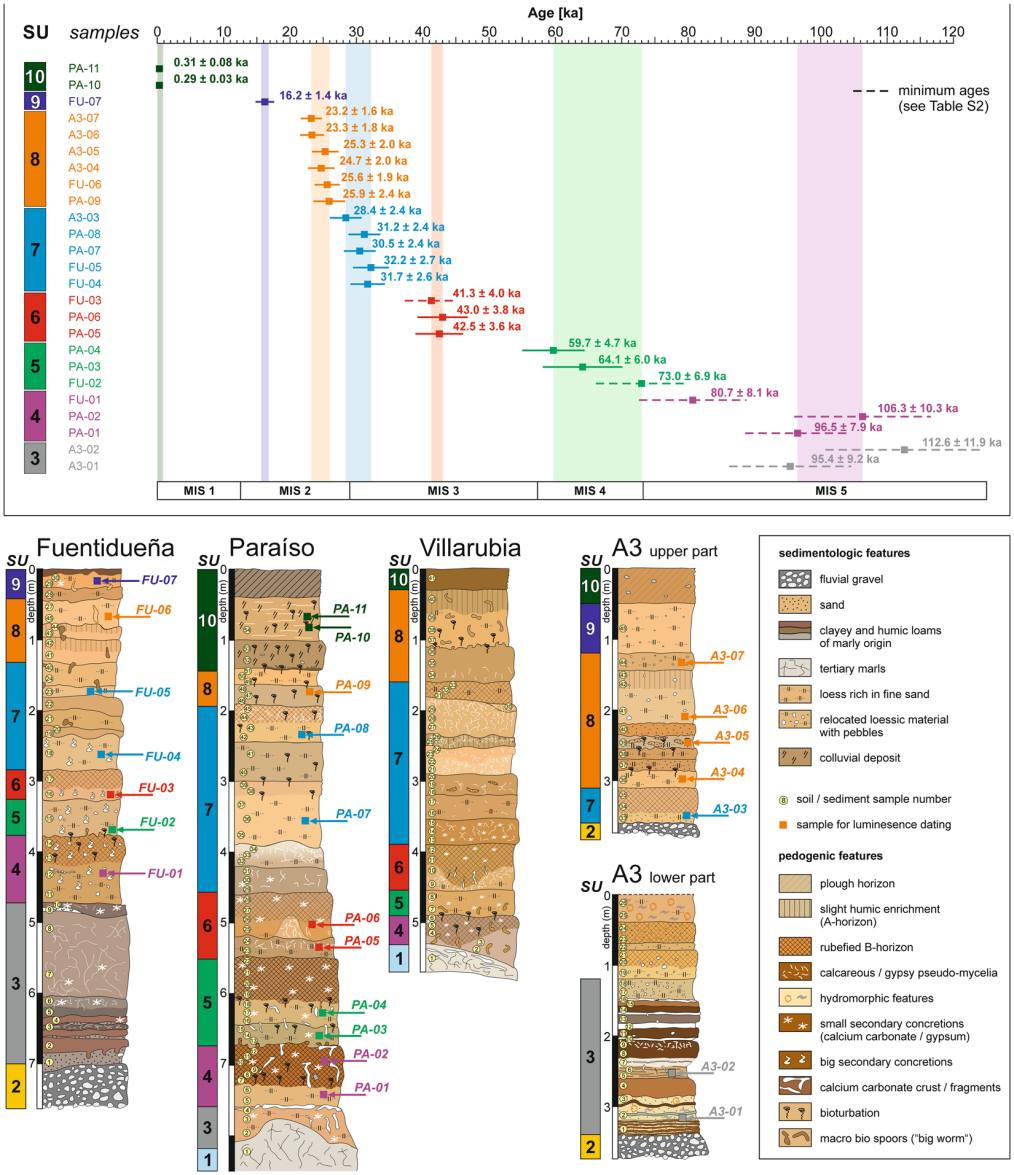
Stratigraphic evidences and age model of the Tagus loess record. Treaded profile sections are shown, together with sampling positions and ages obtained. The classification into sediment units is based on the results presented in Fig. S6. The detailed expose of the OSL ages is to be found in SI Text 5.

In the following, the stratigraphic characterisation of the Tagus loess record aims at differentiating between individual loess layers and providing them with a temporal resolution by means of 25 luminescence ages (Fig. 2). The sedimentary record can be subdivided into 10 stratigraphic units (SU-1 to SU-10) (detailed description in SI Texts 1–4, and Figs S2–S5). The base of the sections is characterized by alternating brownish and clayey sediments (SU-3) resting on fluvial gravels (SU-2) in floodplain positions, and by relocated marls (SU-3) in higher slope positions. Since OSL dating attempts of samples from SU-3 show clear signs of signal saturation, the sedimentation age lies beyond 100 kyr ago (SI Text 5). Environmental information regarding MIS 5 in central Iberia are scarce^26^. However, we interpret SU-3 to be comprised of relocated soil material that has also been generated during the last interglacial, which may point to at least temporarily humid and stable environmental conditions. Subsequently, this soil material has been eroded and deposited by both fluvial and slope processes indicating a period of high landscape dynamics at the transition from the last interglacial to the nascent glacial period. In contrast to the first three units, SU-4 to SU-10 are marked by high loess contents. A first phase of loess deposition (SU-4) probably still occurred during MIS 5, as indicated by dating results (SI Texts 1 and 2). An increased proportion of coarse to medium sand that is unlikely to be transported by wind shows that these sediments were still affected by slope erosion. After a period of stability and intensive soil formation being reflective for more temperate climate conditions (SI Text 7), a first accumulation of pure aeolian loess took place between 73.0 ± 6.9 kyr and 59.7 ± 4.7 kyr (SU-5) (Fig. 2), that gives evidence for cold and arid conditions throughout MIS 4. With respect to the onset of this period, we have to point out that the OSL age of 73.0 ± 6.9 kyr derived from sample BT 1367 has to be interpreted as minimum age. Thus, the accumulation of SU-5 may already have started during a transition period in the late MIS 5. The next phase of geomorphic activity (SU-6) has so far hardly ever been documented by other studies and occurred between 43.0 ± 3.8 kyr and 41.3 ± 4.0 kyr in the middle of MIS 3. Again, the age of 41.3 ± 4.0 kyr has to be interpreted as minimum age estimation, since sample HUB 470 revealed a significant number of aliquots showing distinct signs of dose saturation (Table S2). High contents of fine sand and coarse silt indicate a strong increase in wind speed compared to earlier stages of the last glacial period (Figs S2 and S3), and the narrow range of deposition ages suggests brief and vigorous loess accumulations. Thereafter, another prominent phase of loess deposition (SU-7) started around 32.2 ± 2.7 to 31.7 ± 2.6 kyr, replacing a prolonged period of stability. In the sections Fuentidueña and Paraíso, again the narrow range of deposition ages (32.2 ± 2.7 to 30.5 ± 2.4 kyr) points to high sedimentation amounts concurrently with the Heinrich Stadial 3 during the final stages of MIS 3. Highest contents of calcareous fine sands, and high GSI values (“grain size index”, see Methods) testify strong aeolian activity and probably reflect the driest and coldest conditions during the last glacial. Apart from an isolated dating out of the section A3 (HUB 472; 28.4 ± 2.4 ka), sediments of SU-7 are distinguishable from those of SU-8 based on non-overlapping 1σ-levels of luminescence ages (although there is an overlap in ages when considering the 2σ-uncertainties), but also individual geochemical properties (Fig. S6), which indicates the presence of two separate loess deposition phases. The last two periods of loess deposition took place between 25.9 ± 2.4 kyr and 23.2 ± 1.6 kyr (SU-8), and around 16.2 ± 1.4 kyr (SU-9) that both coincide with Heinrich Stadials 2 and 1. These findings suggest that during the first period of the global Last Glacial Maximum (26,5–19 kyr)^27^, conditions were cold and dry, but changed to relatively mild ones in the later part that fits with evidences from mountain glaciers^28^ and rock shelters^29^ in central Iberia. The uppermost unit (SU-10) consists of colluvial slope deposits and indicates strong sediment shifting most likely related to very recent human land use practices.

## Discussion

**Figure 3:**
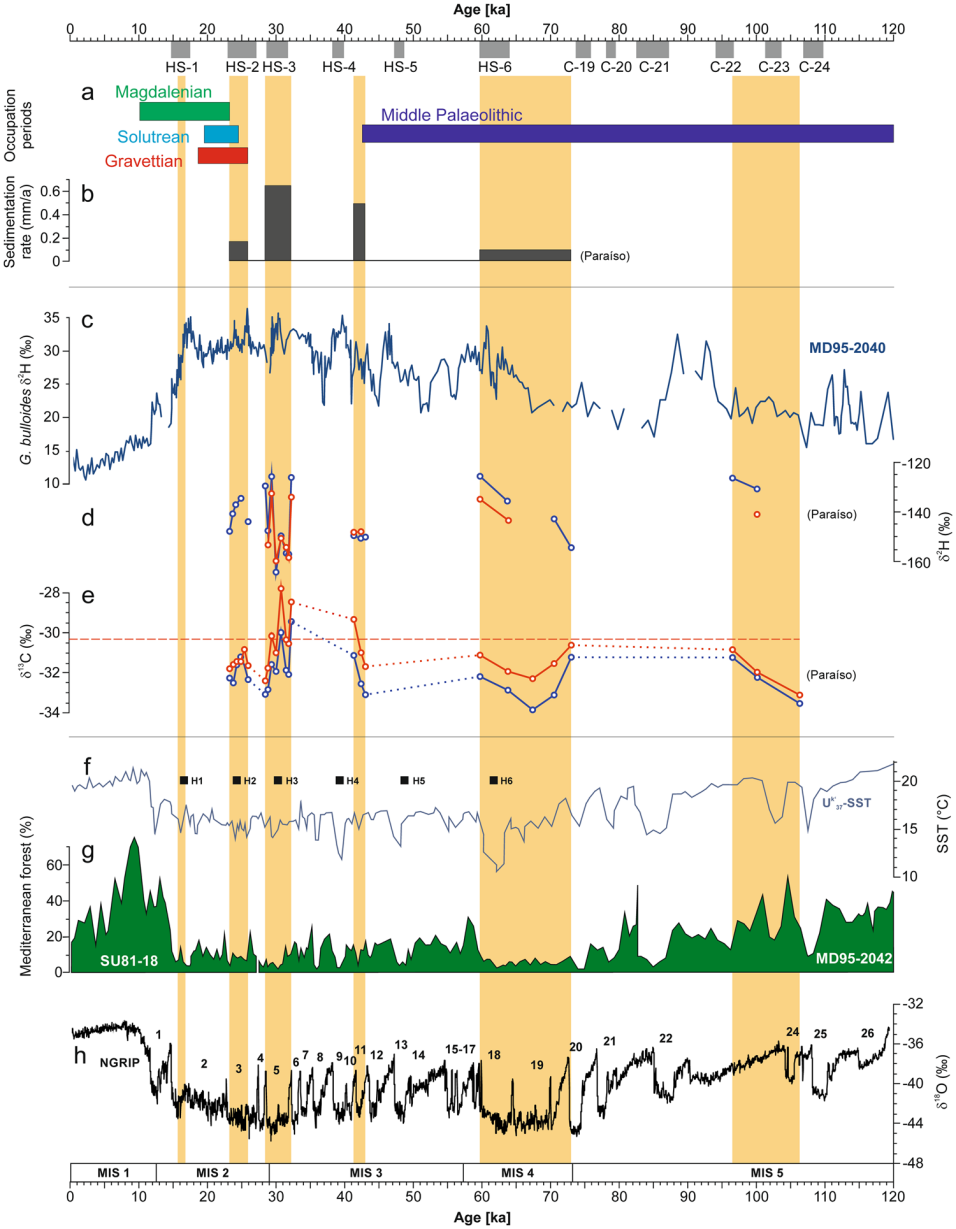
Main loess deposition periods (indicated by vertical ochre bars) in the upper Tagus Basin from the past 120 kyrs compared with human occupation patterns of interior Iberia and other palaeo-climate indicators from marine and ice-core records. (**a**) Temporal placement of Upper Palaeolithic and Middle Palaeolithic occupations of interior Iberia are based on the results presented in SI Text 9. (**b**) Loess deposition rates were estimated based on the thickness of loess units and the mean of obtained OSL ages. (**c**) δ^2^H record on *G. bulloides* of core MD95-2040^36^; δ^2^H values were calculated based on δ^18^O values using the global meteoric water line. (**d**) δ^2^H values of the *n*-alkane compounds n-C_29_ (red) and n-C_31_ (blue) from the loess section Paraíso^35^. (**e**) δ^13^C values of the *n*-alkane compounds n-C_29_ (red) and n-C_31_ (blue) from the loess section Paraíso^35^; the red dotted line marks the estimated limit for habitability of the interior of Iberia. (**f**) SST of marine drilling core MD95-2042 and SU81-18^33^, and Heinrich Events detected in the same core. (**g**) Pollen percentage of Mediterranean forest from core MD95-2042 and SU81-18^33^. (**h**) δ^18^O record of the NGRIP ice core with numbers referring to stadials^46^. Timing of HS-1 to HS-6 (Heinrich stadials) is based on ref.^46^, and C-19 to C-24 (North Atlantic ice rafting events) on ref.^75^.

The chronological order of loess deposition phases testifies the discontinuous nature of that sedimentation record. Accordingly, the archive reveals clearly defined phases of brief loess deposition during the last glacial caused by pronounced environmental aridity. Moreover, the timing illustrates that the Tagus loess record does not directly mirror the succession of Dansgaard-Oeschger cycles. However, during MIS 2 and 4 the synchronicity between phases of loess deposition in central Iberia and Heinrich stadials that are known from the Iberian margin^16,30^ (Fig. 3) shows that intense aeolian activity has been initiated during episodes of major ice sheet collapse following the Bond cycles^31,32^. During these episodes, decreases in sea surface temperatures have been linked to a southward incursion of the polar front over the Iberian margin^33,34^ that may have had serious impacts on inner continental climate situations. The strong coupling between central Iberian environmental conditions and a larger-scale northern hemispheric climate evolution demonstrates that for prolonged periods during the last glacial, information from marine records appear to be highly indicative of environmental dynamics in the continental interior.

However, this does not fully apply to the period of MIS 3. In contrast to MIS 2 and 4, loess deposition between 35 and 50 kyr does not point to a synchronous behaviour of oceanic and continental environments. This means that no loess dynamics were observed in the course of Heinrich Stadial 5 and, instead, loess deposition took place in a time around 41.3 ± 4.0 kyr to 43 ± 3.8 kyr (SU-6) that most likely preceded the actual Heinrich event 4 (H4) (Fig. 3). It has to be mentioned that in SU-6 the calculated luminescence ages are accompanied by relative errors in the range of 8–9%, which does not permit to completely exclude H4 (similar to 40–39 kyr BP). At the same time, however, we should note that for all other loess deposition phases within the last 70 kyr the mean ages show an exact temporal overlap with Heinrich events, or Heinrich stadials. The fact that all OSL dating from SU-6 exhibit mean ages that are several thousands of years higher than the onset of H4 prompts us to consider a period in between H4 and H5 to be connected with aeolian dynamics that caused the deposition of SU-6. An additional indication of inconsistency between H4 and the deposition of SU-6 may arise from δ^2^H analyses on *n*-alkanes that were extracted from bulk samples of the Paraíso section (ref.^35^; see Fig. 3). Generally, δ^2^H values may be sensitive to evapotranspirative enrichment or changes in atmospheric circulation, but according to its hydrograph, we expect that δ^2^H mainly reflects isotopic changes of the source, i.e. the ocean offshore Portugal^35^. The δ^2^H curve from the loess section (Fig. 3) demonstrates that the values of the loess layer SU-6 may be in the lower part of the whole range. Given that on the planktic foraminiferal δ^18^O curve on *Globigerina bulloides* (δ^18^O-values can be expressed as δ^2^H-values using the global meteoric water line: δ^2^H = 8 * δ^18^O + 10) from the core MD99–2343 on the southwestern Iberian margin^36^ the values related to H4 are amongst the highest values of all Heinrich stadials, this may reiterate the position that SU-6 and H4 do not relate to the same phase.

During the period in question, a drastic change has also been recorded related to Palaeolithic settlement patterns in central Iberia, since archaeological findings indicate the sudden disappearance of Neanderthals in the region shortly before 42 kyr. If until recently a late Neanderthal survival (i.e. post-40 kyr) was recognized in central and southern Iberia, including the inland areas^10^, currently such a survival has been falsified in the interior lands of the peninsula due to the re-dating and critical examination of key sites^11,22,37^. Thus, at the moment no archaeological or palaeoanthropological evidence attests for a Neanderthal presence in the complete Iberian interior after 42 kyr ago^37,38^ (Fig. 3, SI Text 9, and Figs S19 and S20). Interestingly, the forerunning Heinrich Stadial 5 neither is marked by loess deposition nor resulted in the final breakdown of the Middle Palaeolithic occupation, although it should be mentioned that archaeological remains are scarce for this period. A revealing aspect may arise from compound-specific δ^13^C analyses on long-chain *n*-alkanes in the Paraíso section (ref.^35^; see Fig. 3) that were used to reconstruct palaeoclimatic and palaeohydrological changes. The basic assumption here is that the δ^13^C signal of leaf waxes can be used to identify the input of C_3_ vs. C_4_ vegetation, while it also depends on the δ^13^C of the atmospheric CO_2_, as well as on its concentration, the air temperature, relative humidity and precipitation during the growing season^39^. It becomes evident that the formation of SU-6 goes hand in hand with a significant increase of δ^13^C (Fig. 3) reaching values of more than −30‰. Since δ^13^C values remained lower during all previous phases of loess deposition we expect that the formation of SU-6 may have been linked to so far unprecedented aridity in the Iberian interior that may be tantamount to the exceedance of a threshold for inhabitability. Therefore, we hypothesize that the end of the Middle Palaeolithic in inner Iberia in a period around 42 kyr corresponded with serious regional climatic deteriorations, which led to sparse vegetation cover, and thus affected availability of key resources for Neanderthals, such as game, plant foods and wood.

This gives rise to the question of what mechanisms are responsible for the fact that inner-Iberian loess deposition phases and Heinrich stadials were exactly in-phase during MIS 2 and MIS 4, but possibly decoupled in a period between 35 and 50 kyr right in the middle of MIS 3. Generally, the build-up of loess deposits requires two preconditions: a sediment source with large quantities of silt^40,41^, and strong winds for mobilization and transport. In case that intensity and frequency of winds are too low, this will prevent sediments from being picked up and blown over^42^. Steepened meridional temperature gradients and seasonality are considered responsible for increasing intensity and frequency of gusty winds during glacial periods^16,31,43,44^. A general polar front presence along the Iberian margin during Heinrich events has been proposed^19^, but one should also consider irregularities and uncertainness regarding the position and seasonal duration of the polar front over continental Iberia, which could explain lower wind strength during Heinrich Stadials 4 and 5. For instance, a northward deflection of the jet stream during the middle MIS 3^45^ supported by a blockading effect by northern Iberian mountain ranges, and/or a high-pressure area over Iberia may have led to lower storm activity in central Spain. On the other hand, a lack of deflatable material may likewise cause the absence of loess deposition, even in case of frequent storms. With the main source of loess being the Tagus floodplain, a lack of fine floodplain sediments may be caused either by a transport-limited system (e.g. with absolute dryness preventing an appreciable generation of runoff, fluvial transport, and floodplain sedimentation) or by a supply-limited system (e.g. with just minimal sediment supply because of strongly reduced sediment weathering processes). The first scenario seems to be rather unrealistic, especially with regard to the conformity of loess deposition and Heinrich events over the last 35 kyr. The second case may reflect strongly reduced physical weathering in the mountain ranges of the headwaters probably due to a decrease of periglacial (cryoclastic) processes in the course of somehow warmer climate conditions. In summary, with regard to Heinrich Stadial 5 it might be possible that the absence of loess deposition has been caused by a combination of less storm activity and reduced sediment supply due to a northward deflection of the polar front and a corresponding transport of warmer air masses from southern directions. The same may apply to H4, although we are currently unable to exclude loess deposition during that period because of the uncertainties regarding OSL dating. To better define the age range of the deposition of SU-6 should be a focal point in further research activities. However, in case that loess deposition did not take place at the same time but previously to H4, it will be challenging to uncover the mechanisms behind. Generally, H4 is a period of pronounced sea temperature cooling on the southwestern Iberian margin, and it is assumed that it exercised a great influence on central and southern Iberian environments^17^. One possibility would be that a cold period related to Greenland Stadials 11 or 12 (41.4–44.3 kyr BP)^46^ caused highly arid conditions over the Iberian interior that led to the generation of loess. Another possibility would be that the deposition of SU-6 related to somehow warmer and arid instead of cold and arid conditions, which in turn is not reflected by marine records. However, without using dating methods that are more precise this will remain a matter of speculation.

Everything seems to suggest that the deposition of loess layer SU-6 indicates the development of extraordinary aridity in the Iberian interior. According to our evaluation of the depositional ages, we suggest that this period of climatic deterioration together with emerging dust-loaded gusty winds played a significant role for the worsening of human living conditions, and thus the disappearance of Middle Palaeolithic populations from central Iberia. Increased evaporation in the course of the so far driest conditions of the last glacial may have contributed to a pronounced life-hostility during the middle and upper MIS 3 characterized by diminished vegetation cover, reduced bioproductivity and lesser prey. Thereby deprived of their livelihoods, Middle Palaeolithic populations would have had hardly any chance for surviving in such an inhospitable area. Going one step further, a higher atmospheric humidity even during periods of loess deposition may explain the continuity of Neanderthal occupation throughout MIS 4 (Fig. 3). In this respect, the archaeological record (SI Text 9, Tables S7 and S8, and Figs S19 and S20) suggests that the withdrawal from the inner-continental areas around 42 kyr happened without any intervention of AMHs and was therefore tantamount to a contraction of human settled areas, which contradicts existing simulations^5^. So far, although the presence of AMHs has been documented in northern Spain already for a time as early as *c*. 42 kyr cal BP^47^, there is no evidence of a later reuse of the inner-continental areas until around ~28 kyr cal BP for the peripheral fringes of the Meseta, and until ~25.5 kyr for the Meseta itself^48^ (S1 Text 9), neither by Neanderthals nor by AMHs. This supports the idea that environmental and climatic conditions largely determined the habitability of harsh zones for human populations, and that the interior of Iberia turned into a hostile and desolate area at least in a phase between ca. 42 and 25.5 kyr. Perhaps, this hostility culminated between ~32.2 ± 2.7 and 28.4 ± 2.4 kyr in a period of strongest aeolian activity as reflected by highest sedimentation amounts and maximum δ^13^C values concurrent with Heinrich Stadial 3 (Fig. 3). During these times, life had to be concentrated in climatically more favourable regions, such as along the Iberian coastline, since here climate extremes were attenuated and human population was less affected compared to the continental regions^49^.

After 32 kyr, when Heinrich stadials and central Iberian loess deposition occurred simultaneously again, we are faced with a changed situation of human-environment relations. Firstly, archaeological finds indicate that during MIS 2 Gravettian, Solutrean, and Magdalenian population dynamics displayed a roughly parallel development in inner and outer parts of the Iberian Peninsula^21^ suggesting that unfavourable climate conditions played a lesser role in constraining the utilisation of inner-Iberian sites. This means secondly that after 25.9 ± 2.4 ka in interior Iberia, the coincidence of a period that was cold but not quite as dry (Fig. 3e) and the onset of different Upper Palaeolithic cultural stages (Fig. 3a, Fig. S20) may illustrate a strong adaptability of AMHs to still harsh but a little less hostile environmental conditions. However, in view of the current scarcity of chronologic information related to the Upper Palaeolithic in central Iberia, this could only be a hypothesis that needs further research and empirical data (SI Text 9, Table S8).

Finally, our findings show a strong coupling of marine processes and atmospheric conditions over inner Iberia during the last glacial related to the occurrence of cooling events (HEs), with the exception, however, of the middle MIS 3 that does not indicate a clear agreement. As long as no verified age information is available for that specific period, we have to assume that regional conditions may have strongly overprinted large-scale climate fluctuations. Pursuant to our current interpretation of the data, adverse environmental conditions, dominated by a decrease in the vegetation cover and thus in the availability of biotic resources, appear to be connected to the exodus of Middle Palaeolithic populations from climatically disadvantaged regions of the Iberian interior around 42 kyr, and their probable movement to the coastal areas of the peninsula^37^. Here, current data still supports a Neanderthal survival until *c*. 37 kyr^50^ that consequently brings coastal sites as areas of retreat into focus when addressing the potential encounter of Middle and Upper Palaeolithic populations^51^, which however, is currently a matter of strong debates^10,14,50^.

## Methods

### Stratigraphic work

In total, we selected four profile sections for in-depths laboratory analysis. Overall, 178 samples were taken for soil physical, sedimentological and geochemical analyses. Moreover, we took 10 samples for micromorphological studies, and 12 samples for heavy mineral analysis. Sampling was generally oriented towards stratigraphic layers and soil horizons.

Grain-size determination was conducted by pipette analyses and wet sieve techniques^52^ after dispersion with sodium pyrophosphate. Because the samples contained certain quantities of gypsum that disrupted the settling process in the sample cylinder by flocculation, all samples passed through a repeated cycle of dissolution and centrifugation until measured electrical conductivity fell below a value of 400 µS cm^−3^ ^53^. For determining fraction-specific carbonate contents for different grain-size classes, we conducted grain-size analysis twice. One run was implemented without decalcification, another run took place after carbonates were dissolved by using 10% HCl. Following this procedure, a Grain Size Index (GSI) was calculated based on the carbonated samples by forming the ratio between the coarse silt fraction (>20 μm and <63 μm) and all finer fractions including medium silt, fine silt, and clay. The calcium carbonate content was determined by measuring the carbon dioxide gas volume after adding hydrochloric acid in a Scheibler apparatus^52^. Soil organic matter was measured via suspension and catalytic oxidation (TOC-VCPN/DIN ISO 16904). Total iron content (Fe(t)-values) was determined after pressure digestion with concentrated nitric and hydrofluoric acid using atomic adsorption spectrometry. Pedogenic iron content (Fe(d)-values) was measured after dithionite extraction using atomic adsorption spectrometry as well^52^. The ratio between pedogenic and total iron may provide information of the intensity of weathering processes in a specific sediment unit, and thus may indicate soil formation in case of an increase of values from parent material to weathering horizons.

Magnetic susceptibility was measured equidistantly every 5 cm in SI units using a Bartington MS2 susceptibility meter and S2F probe^54^ by taking the average of four measurements. Furthermore, all 178 samples that were taken for laboratory analysis were used for rock magnetic measurements that were conducted at the Laboratory for Palaeo- and Environmental Magnetism at the University of Bayreuth. In this study, initial low field magnetic susceptibility was measured on a frequency of 0,3 kHz using the MAGNON VFSM susceptibility bridge (320 Am^−1^ AC field). This measurement characterizes the volume susceptibility К and divided by the density of the sample it delivers the mass specific susceptibility χ (Fig. S6).

For micromorphological analyses, oriented and undisturbed soil samples were collected from selected horizons of the sequence Paraíso (Fig. S12). Vertical thin sections were prepared at the Laboratory of Soil Science and Geoecology at the Institute of Geography (Eberhard-Karls-University Tübingen). Using a polarizing microscope (Zeiss Axiovision) the thin sections were analyzed and photographed at the Institute of Physical Geography (Goethe-University Frankfurt/Main). The description of the thin sections is mainly based on the terminology after ref.^55^ and ref.^56^.

For compound-specific stable isotope analyses of δ^13^C and δ^2^H, we used the aliphatic lipid fraction containing the *n*-alkanes. Total lipid extraction and separation into lipid fractions is described in ref.^57^. The compound-specific carbon isotope measurements were performed on an IsoPrime 100 mass spectrometer, coupled to an Agilent 7890 A GC via a GC5 Pyrolysis/Combustion interface that operated in combustion mode (CuO reactor) at 850 °C. Samples were injected using a split/splitless injector in splitless mode. The GC was equipped with a 30 m fused silica column (HP5-MS, 0.32 mm i.d., 0.25 µm film thickness). The precision was checked by injecting a standard alkane mix (C27, C29, C33) with known isotopic composition (Schimmelmann) twice every six runs. The analytical error was generally better than 0.5‰ (triplicate measurements). The stable carbon isotope composition is given in the delta notation (δ^13^C) versus Vienna Pee Dee Belemnite (V-PDB). For the compound-specific hydrogen isotope measurement, the GC5 mode was changed to pyrolysis by using a Cr (ChromeHD) reactor at 1000 °C. Adjustments for the GC, the standard and the injection procedure were the same as for the stable carbon isotope measurements. The H3+ -correction factor was checked every two days and was stable at 3.48 ± 0.06. The analytical error was generally better than 5‰ (three replicates), and we report only values with a standard deviation better than 6‰. The stable hydrogen isotope compositions are given in the delta notation (δ^2^H) versus Vienna Standard Mean Ocean Water (V-SMOW).

For heavy mineral analysis, we examined 10 samples from the loess section Paraíso (Fig. S12) including the underlying Tertiary marls. For assessing the mineralogical signature of potential loess source areas, we analyzed reference samples from the three main surrounding geological units that are (i) Tertiary marls, (ii) metamorphic units and granites from Palaeozoic rocks, and (iii) Mesozoic sedimentary rocks (Fig. S1). The latter two are represented by sandy floodplain sediments of the Tagus River (draining Mesozoic rocks of the Iberian Range and the Sierra de Altomira), and the Algodor River (draining Palaeozoic granitoid rocks of the Montes de Toledo).

The separation of heavy minerals was conducted after the procedure described by ref.^58^ including drying of the samples, sieving to a grain size fraction between 40 µm and 400 µm, removing of carbonates by adding acetic acid, eliminating gypsum by repeated soaking and sieving, dispersing with Na_4_P_2_O_7_, and finally, separating of the heavy mineral fraction by using sodium polytungstate. For the identification of the heavy minerals, we produced strewn slides after ref.^59^ for transmitted light microscopy by coating with gelatin, and unilateral embedding in the immersion fluid α-chloronaphtalene with a light refraction of 1,633. For optimal analyses, we identified and counted at least 200 translucent mineral grains. The determination of translucent minerals was based on grain morphologies, colours, pleochroism features, fissility, break, light refraction, double refraction, as well as inclusions. For reasons of clarity, some minerals groups such as mica, tourmalines, or garnets were grouped together, since an optical representation of more than 10 different minerals is hardly feasible.

### Age determination

Luminescence dating was performed on 25 samples. We applied OSL measurements on the coarse grain quartz fraction (90–200 µm). Following standard procedures^60^, sample preparation was done under subdued red-light conditions (640 ± 20 nm). After wet sieving of the sediment, we removed carbonates and organic material by using 10% HCl and 10% H_2_O_2_, respectively. The remaining material was subjected to density separation using sodium polytungstate in order to separate quartz from heavy minerals (density > 2.75 g/cm^3^) and feldspars (density < 2.62 g/cm^3^). We etched the so gained quartz separates in 40% HF for 50 minutes and finally washed in 10% HCl for 30 minutes. The purity of the resulting quartz samples was checked by IR stimulation, determining the OSL IR depletion ratio^61^ and by visual inspection of the TL curve shape, i.e. the 110 °C TL peak^62^.

For OSL measurement, quartz grains were mounted on aluminium cups and fixed with silicon oil. Thereby, a 3 mm mask was used which provided so called ‘small aliquots’, restricting the number of grains to approximately 100–300 grains per disc. Except for samples HUB 470, HUB 471 and HUB 472, which were measured at the Humboldt University in Berlin, all luminescence measurements were carried out at the University of Bayreuth using an automated Risø-Reader TL/OSL-DA-15, equipped with a ^90^Y/^90^Sr β-source for artificial irradiation. Blue LEDs (470 ± 30 nm) were used for OSL stimulation and the luminescence signal was detected by a Thorn-EMI 9235 photomultiplier combined with a 7.5 mm U-340 Hoya filter (290–370 nm).

All luminescence shine-down curves were recorded for a total of 40 seconds at an elevated temperature of 125 °C, using a single aliquot regenerative-dose (SAR) protocol^63^, which was enhanced by an additional hot-bleach step at the end of each test dose measurement^64^. Equivalent doses were calculated with an R-script based on the R-package ‘Luminescence’, version 0.7.3^65–67^, using the first 0.6 seconds of the measured OSL signal after subtracting a background which was derived from the last 7.5 seconds of the recorded signal.

All aliquots that did not pass the commonly applied rejection criteria for OSL dating were excluded. Additionally, the OSL IR depletion ratio was calculated for each aliquot in order to discard all aliquots still contaminated by feldspar remnants^61^. Thus, only aliquots with a recycling ratio of 0.9–1.1, a recuperation of ≤5% of the natural sensitivity corrected signal intensity^63^, an OSL IR depletion ratio in the range of 0.9–1.1 and a signal not lower than 3 times the background were accepted for equivalent dose calculation. As the dose response curves of some investigated samples (BT 1376, BT 1368, BT 1372, BT 1374, BT 1383, BT 1384 and HUB 470) suggested the possibility of these samples being either in field saturation or at least close to their saturation levels, we also applied the ‘2D_0_-criterion’^68^ as additional rejection criterion. Therefore, aliquots were only regarded as reliable and considered for equivalent dose determination if their respective natural signal did not exceed the threshold of 2 D_0_. At this point, we would like to emphasize that^68^ did not suggest the 2D_0_-value as rejection criterion, but only proposed to use it as an indicator for aliquots being close to saturation. When using this value as additional rejection criterion, the upper part of the equivalent dose distribution will artificially be truncated, most probably resulting in mean D_e_-values that will seriously underestimate the true equivalent doses (e.g.^69,70^). Therefore, we emphasize that all ages derived from samples showing a large proportion of aliquots rejected due to the 2D_0_-criterion may underestimate the true burial age and can only be interpreted as minimum ages. The 2D_0_-criterion was uniformly applied for all samples, however, proved only to be problematic for the samples mentioned above.

The interpretative value of age determinations based on luminescence dating strongly depends on the luminescence properties of the used dosimeter and the performance of the applied measurement protocol. A measure for both is the dose recovery rate, which indicates how accurate the used protocol can recover a known dose from a bleached and artificially irradiated sample^71^. In order to determine the optimal preheat-temperatures and to assess the samples’ suitability for dating purposes, combined dose-recovery and preheat tests were conducted for each sample. After artificially bleaching the samples for 3 hours using a solar lamp (Osram Duluxstar 24 W), they were β-irradiated with known doses close to the equivalent doses expected for the investigated samples. These known doses were treated as unknown and determined by applying the SAR procedure described above, splitting the aliquots into five groups for which different preheat temperatures in the range of 180 °C to 260 °C (steps of 20 °C) were used. For each group the measured-to-given dose ratio was calculated. Based on this ratio, an individual preheat temperature was chosen for each sample for which the given laboratory dose could be recovered at its best.

For dose rate $(\dot{{\rm{D}}})$ determination, the U- and Th-concentrations were detected by thick source α-counting, the K-contents of the samples were measured by ICP-OES. Calculations for determining the environmental dose rate were done applying DRAC v1.2^72^ in combination with the conversion factors given by ref.^73^. An interstitial water content of 5 ± 3% was assumed to be representative for the burial period, derived from measurements of the present day water contents and considering both, sedimentological properties of the specific samples and differences in the geographical settings of the locations. Only for samples BT 1375, BT 1381, BT 1383, BT 1384 and BT 1544 a deviant water content of 8 ± 3% was used. Cosmic dose rates were calculated according to ref.^74^ using the ‘calc_CosmicDoseRate’-function provided by the R package ‘Luminescence’^65–67^.

## Electronic supplementary material

Supplementary Information
