# Supplementary material for: Climate deteriorations and Neanderthal demise in interior Iberia
Source: Sci Rep. 2018 May 4;8:7048. doi: 10.1038/s41598-018-25343-6 (PMC5935692; doi:10.1038/s41598-018-25343-6)
Supplement: Supplementary file 1 — Supplementary Information [file 41598_2018_25343_MOESM1_ESM.pdf]

Supplementary Information

Climate deteriorations and Neanderthal demise in interior Iberia

Daniel Wolf[1]*, Thomas Kolb[2], Manuel Alcaraz-Castaño[3,4], Susann Heinrich[1], Philipp Baumgart[1], Ruben Calvo[5], Jésus Sánchez[5], Karolin Ryborz[1], Imke Schäfer[6], Marcel Bliedtner[6], Roland Zech[6,7], Ludwig Zöller[2], Dominik Faust[1]

Affiliations:

[1]Institute of Geography, Technische Universität Dresden, Helmholtzstr. 10, D-01069 Dresden, Germany.

[2]Institute of Geography, Universität Bayreuth, Universitätsstr. 30, D-95440 Bayreuth, Germany.

[3]Área de Prehistoria, Universidad de Alcalá, Calle Colegios 2, 28801 Alcalá de Henares, Madrid, Spain.

[4]Neanderthal Museum, Talstraße 300, 40822 Mettmann, Germany.

[5]Departamento de Ingeniería Civil y de la Edificación, E.T.S.I. Caminos, Canales y Puertos, Universidad de Castilla La Mancha, Ciudad Real, Spain.

[6]Institute of Geography and Oeschger Centre for Climate Change Research, University of Bern, Hallerstr. 12, CH-3012 Bern, Switzerland.

[7]Institute of Geography, Friedrich-Schiller-Universität Jena, Löbdergraben 32, D-07743 Jena, Germany.

*Corresponding Author: daniel_wolf@tu-dresden.de (D.W.)

SI Text 1

Section Fuentidueña

This section is situated on a late Pleistocene terrace of the Tagus River between Villamanrique de Tajo and Fuentidueña de Tajo (N 40°05'14,76"; W 03°12'18,03"; 560 m a.s.l.). The base of the profile has an elevation of 13 m above recent river level and rests on poorly sorted and well-rounded gravels (SU-2) (Fig. S2). The bottom unit (SU-3) consists of alternating sandy and loamy layers with dark grayish to brownish colors. Compared to the upper part of the section, the main features of SU-3 are high contents of soluble salts (between 25 and 59 %), low contents of sands and silt, comparably high contents of clay, and accordingly the lowermost values of the Grain Size Index (GSI) ranging between 0 and 0.8. Moreover, magnetic susceptibility reaches relatively high values with χ > 20 x 10^{-8} m^3 kg^{-1}. Overall, this suggests that SU-3 is dominated by relocated soil material derived from surface erosion of the surrounding evaporate marls. The alternate layering indicates a mixture of fluvial and slope processes during sedimentation, which may suggest sedimentation in an active floodplain environment prior to the incision of the Tagus River into the terrace body of SU-2.

Above SU-3, units SU-4 and SU-5 are characterized by loess-like sediments, which means that a considerable amount of loess has been accumulated and was accompanied by relocation processes along the slope. This is evidenced by a strong increase of calcareous silts representing aeolian components, as well as still high proportions of the medium sand fraction that is too coarse to be transported by wind. Furthermore, small pebbles with diameters up to 5 mm were found in SU-4 and SU-5. SU-4 is marked by an extensive occurrence of calcified root cells and carbonate nodules in the lower part with maximum carbonate contents of > 45 %. The coarse sand fraction reaches up to 2.5 % but consists mainly of secondary carbonate concretions, while medium sand is largely siliceous. SU-4 concludes with an intensive palaeosoil that is indicated by a dark reddish ochre color, slightly higher clay contents and the maximum in magnetic susceptibility. This soil bears numerous bioturbation features, and especially the upper boundary was strongly dissolved by bioturbation. OSL-dating of unit SU-4 shows clear indications of signal saturation pointing to a sedimentation older than 80.7 ± 8.1 ka. The sedimentation of SU-5 and thus, the end of the soil formation in SU-4 is evidenced by the OSL age of 73.0 ± 6.9 ka.

In SU-6 the share of coarse silt exceeds 50 %, fine sand exceeds 20 %, and the clay content is below 15 %, which results in maximum GSI values of nearly 2.0. This point to very strong wind speeds in a period around 42.5 ± 2.9 ka according to OSL dating. The upper part of SU-6 has a slightly dark reddish ochre color and the lower part shows weak features of calcite hypo-coatings. However, neither magnetic susceptibility nor iron

dynamics and clay contents provide any indications of pedogenesis. Thus, we propose that the weak coloring and carbonate enrichment were caused by a long-time exposure of the surface under a hygrally dry environment, which did not allow for the initiation of serious pedogenic processes. A contribution of remote dust rich in hematite can just be assumed.

After a period of lacking loess deposition, the sedimentation of SU-7 started at about 31.7 ± 2.6 ka and lasted only a short time according to OSL dating. With more than 1,5 m, SU-7 is the thickest unit of the profile indicating high sedimentation amounts and rates, respectively. Silt contents stay on a high level, which is also reflected by permanently high GSI values. The rise of fine sand contents to more than 27 % may be taken as an indication of the highest wind speeds in the area during the whole last glacial. Calcite hypo-coatings in the lower part of SU-7 may again point to weak re-calcification during a slightly wetter interim phase. The whole unit is free of gypsum and related salts. Beginning in the middle part of SU-7, the complete upper profile is permeated with macro biospurs that are reminiscent of large worm traces up to 4 cm in diameter.

The next unit SU-8 is characterized by a slightly pale ochre color, and high silt and fine sand contents. Magnetic susceptibility reaches lowest values in the profile ($\chi < 7 \times 10^{-8}$ m^3 kg^{-1}), and OSL dating suggests loess sedimentation in a period between ~26 kyr and ~23 kyr.

The section is completed by SU-9 that consists of thin layers of partly relocated material. The upper layer has a light reddish-ochre color, and contains carbonate nodules and pebbles up to 3 cm in diameter. Coarse silt contents strongly decrease, while proportions of fine sand but also medium sand increase. OSL dating suggests that the deposition of this relocated material took place at about 16.2 ± 1.4 ka. There are no indications of a soil formed during the late glacial or Holocene apart from a brown plough horizon, proving serious surface erosion dynamics during the recent past.

SI Text 2

Section Paraíso

The section Paraíso is situated in a more elevated position, approx. 50 m above the recent river level, resting on strongly dissected valley flanks that were developed in evaporate marls. This position is located 10 km east of Aranjuez and south of the village of Balcón del Tajo (N 40°01'51,07"; W 03°28'01,70"; 556 m a.s.l.) with a straight distance of about 24 km from the section of Fuentidueña.

At the base of the Paraíso section (Fig. S3), whitish marls (SU-1) consist to 78 % of soluble salts and possess a carbonate content of 9 %, which is the lowest value that has been measured in all profiles. The overlying SU-3 is characterized by relocated marl material, but carbonate contents of up to 50 % and soluble salts with a share of solely

10 % indicate a changed source area most probably related to calcareous marls and limestone that are exposed further upslope. An increase of clay up to 21 % as well as higher iron contents and higher values of magnetic susceptibility (χ = *44.4* x 10^{-8} m^3 kg^{-1}) document that the material was affected by certain weathering processes prior to its accumulation.

The subsequent unit SU-4 is marked by an increase of calcareous silt (>45 % of the whole coarse silt fraction) and fine sand (>9 %) and thus, a GSI reaching up to 1.3. This evidences the contribution of loess, whereas angular stones up to 4 cm thick reflect the influence of relocation processes along the slopes. In the upper part of SU-4 we found a strongly weathered palaeosoil with a strong reddish ochre-brown color and plenty of carbonate nodules as well as up to 15 cm long carbonate concretions with a rizolith-like shape. The lower part of SU-4 shows about 20 % more carbonate than the upper part indicating strong decalcification and recalcification dynamics. At the boundary between SU-4 and SU-3, a massive crust that is 3 cm thick and contains 35 % of calcium carbonate and 36 % of soluble salts may be linked to this soil formation, too. The whole palaeosoil shows bioturbation features and especially the upper and lower boundaries were strongly frayed. All in all, this palaeosoil appears to be the most intensely developed soil that was found in last glacial sediments, which is additionally supported by the maximum contents of carbonate-free clay (> 19%) and the maximum values of magnetic susceptibility (χ ~ *50* x 10^{-8} m^3 kg^{-1}). OSL dating suggests that SU-4 has been accumulated around 96.5 ± 7.9 and 106.3 ± 10.3 ka, and that the palaeosoil in SU-4 has been exposed until about 64.1 ± 6.0 ka when the accumulation of SU-5 began.

SU-5 is marked by an ochre-brown color, and a low GSI, mainly with values below 1.3. This is caused by decreasing silt but also fine sand contents, and may be indicative of less strong wind speeds. In the upper part of SU-5, another palaeosoil developed, but less intense than the palaeosoil in SU-4. It has a dark pale reddish-brown color and bears a large number of carbonate nodules up to 2 cm in diameter. The palaeosoil shows carbonate contents of about 28 %, while the loess below is slightly carbonate enriched with values of up to 40 %. There is only a minimal increase in magnetic susceptibility, and especially in the lower part of the palaeosoil we found numerous macro biospurs. The palaeosoil has been exposed until about 42.5 ± 3.6 ka when a vigorous loess sedimentation started (SU-6).

The following unit SU-6 is noticeable by the first high peak of the GSI that surpasses the value of 2.0. This reflects the highest values of calcareous coarse silt in the whole profile (59 %) pointing to a maximum in wind speed. That is likewise supported by an increasing fine sand content. In figures S2 to S5, we have shown the percentage of the carbonate

content that is attributable to the coarse silt fraction. In SU-6, the main portion of carbonate appears in form of coarse silt. Taking the results from all sections together, we observed that layers with a particularly high loess content are likewise characterized by a particularly high share of coarse silt on the carbonate fraction.
At first sight, the upper part of SU-6 shows signs of a palaeosoil with a reddish ochre-brown color and a sharp boundary with high concentrations of calcified root cells in the lower part. However, all available information including magnetic susceptibility, clay contents, and pedogenic iron do not indicate the presence of soil forming processes. Thus, we propose that also in the Paraíso section the upper part of SU-6 shows a more intense color because of a prolonged surface exposure under environmental conditions that hampered the occurrence of strong soil formation processes.

The subsequent SU-7 with a thickness of more than 2.5 m shows signs of a short period of strong loess deposition around 31.2 ± 2.4 ka and 30.5 ± 2.4. The loess has mainly a slightly reddish-ochre color, and just in the lowermost and the middle part of SU-7 two layers with a pale grayish-ochre appear. SU-7 shows consistently high values of coarse silt, the highest contents of fine sand of the whole profile (partly more than 30 %), and even the medium sand fraction shows some weak swings. Accordingly, the GSI reaches the maximum values with 2.25 in the uppermost part of SU-7. Although this uppermost part reveals a more intense reddish-ochre color and many macro biospurs, it is linked with very low clay contents, a minimum in magnetic susceptibility, and a peak in carbonate content. Thus, ones more the coloring may indicate a certain stability period with surface exposure and lacking loess sedimentation, but no serious soil forming processes.

The youngest layer that is directly linked to loess deposition in the Paraíso section is SU-8 with a slightly pale grayish-ochre color in the lower part and a strong reddish ochre color in the upper part. SU-8 reaches only half a meter in thickness, whereas the transition to the layer above is marked by a sharp erosion discordance, thus there is no indication of the original thickness. The GSI strongly decreases that is equally reflected by higher contents of clay and medium silt, and a lower content of coarse silt. The magnetic susceptibility shows minimum values ($\chi \sim 5$ x 10^{-8} m^3 kg^{-1}), and OSL dating suggests a sedimentation age of 25.9 ± 2.4 ka.

The uppermost unit SU-10 is comprised of a pale reddish and grayish ochre material with lighten silty bands that may represent washing surfaces linked to erosion processes. The lower boundary is sharp and undulating and reveals a blackish fringe probably indicating burning by fire. Furthermore, pebbles up to 2 cm in diameter appear. The material is strongly enriched in organic carbon, and shows higher values of magnetic susceptibility ($\chi > 15$ x 10^{-8} m^3 kg^{-1}). All these evidences, together with OSL dating pointing to

0.29 ± 0.03 and 0.31 ± 0.08 ka in the upper part and a radiocarbon dating of 4.85-5.46 ka cal. BP in the lower part (Table S4), suggest that SU-10 consists of mid- to late Holocene slope deposits that were derived by the erosion of upper soil horizons in the surrounding area.

SI Text 3

Section Villarubia

The section of Villarubia is situated in a similar position to the Paraíso section, but some 4 km further east and approximately 93 m above recent river level (N 40°01'42,2"; W 03°25'30,3"; 607 m a.s.l.). It is exposed in a road-cut on a hilltop position, and the stratigraphic succession is very similar to the Paraíso section. The profile is about 5.5 m thick and rests on weathered whitish to grayish marls (SU-1) that were probably relocated (Fig. S4). The content of soluble salts within the underlying marls amounts to 48 % while the carbonate content reaches 29 %, and magnetic susceptibility values are below 4×10^{-8} $m^3 kg^{-1}$ (Fig. S6). Above the marls, SU-4 reveals a pale reddish-gray colored palaeosoil that is developed in relocated rose gray marl material. There are plenty of refilled macro biospurs, and the magnetic susceptibility shows maximum values with $\chi \sim 20^{-8}$ m^3 kg^{-1}. The upper part of the soil shows a carbonate content of 23 % and the layer below is enriched to 42 % indicating de- and recalcification processes.

First contributions of loess appear in SU-5, whose characteristics are rather unspecific. Results of grain size analysis show that the largest portion of $CaCO^3$ is attributable to coarse silt, which is an indication for a high loess content. An increase of coarse silt is visible in SU-6 that is likewise confirmed by an increase of the GSI. In contrast to the other profiles, we found a palaeosoil formed within SU-6 that is partly reflected by analytical results. For example, iron compounds show a slight increase of the Fe(d)/Fe(t)-ratio. The palaeosoil has an intense reddish ochre color and its lower boundary shows a sharp transition towards a pale ochre material that with intense carbonate nodules and calcified root cells. However, carbonate contents do not reflect these patterns and increase in the upper part of the soil that is most probably due to secondary recalcification.

The lower part of SU-7 has a darker reddish and grayish-ochre color, with secondary carbonate concretions (medium sand fraction consists primarily of carbonate), calcified root cells, and small sandy lenses in a depth of 3 m that probably indicate short distance relocation by slope processes. The upper part of SU-7 is characterized by pure loess with pale and slightly reddish-ochre colors, and maximum GSI values (~2.5). At least one temporarily stable surface is indicated by a reddish coloring and a concentration of fine calcified root cells below in a depth of 2.6 m. Likewise the uppermost part of SU-7 shows a strong and homogeneous reddish-ochre color, and a sharp boundary of calcified root

cells below and may indicate a prolonged period of surface stability. Here, also the magnetic susceptibility shows higher values with $\chi \sim 10^{-8}\ m^3\ kg^{-1}$.

The last loess layer in the profile is SU-8 with an intense reddish and yellowish-ochre color and numerous macro biospurs. The top is slightly darker and enriched in organic carbon and may probably relate to a late Pleistocene to Holocene soil formation. SU-8 shows lower contents of soluble salts (<5 % that is below the limit of detection) which may be linked to the Holocene leaching front.

SI Text 4

Section A3

Section A3 is located in the east of the study area related to a quarry close to the highway A3 (N 40°06'38,9"; W 03°08'47,5"; 560 m a.s.l.). The base of the profile is 10 m above the recent river level resting on a massive body of poorly sorted and well-rounded gravels (SU-2). The palaeo-relief linked to the surface of these gravels is strongly undulating, probably because of a juxtaposition of gravel bars and channels. Thus, the section A3 is split into two parts with the lower part being situated in the deepest point of a former channel, and the upper part being situated on the former top of a gravel bar.

The lower part of the profile is dominated by fluvial sedimentation patterns and the presence of sandy material and pebbles (Fig. S5). At the base, unit SU-3 spreads over more than 2 m. It is characterized by alternating layers of dark brown clayey soil sediments with clay contents of up to 55 % and a magnetic susceptibility of more than $50^{-8}\ m^3\ kg^{-1}$ on the one hand, and sandy layers with partly more than 50 % of fine and medium sand, on the other hand. The clayey layers are separated by hardened laminar carbonate accumulations that contain up to 66 % of carbonate, which is mainly attributable to the different sand fractions. These layers were most probably fed by lateral runoff saturated with carbonate. OSL dating that was realized on two samples from below the clayey layers revealed apparent ages of 112.6 ± 11.9 and 95.4 ± 9.2 ka. Because of clear indications of signal saturation, both ages should be considered as minimum ages, thus pointing to a sedimentation period distinctly older than 100 ka. We take this as a further indication that SU-3 may be mainly comprised of eroded and relocated material from last interglacial soils in the catchment.

Above SU-3, sediments show fine sand contents of more than 20 %, and medium sand contents of more than 4 and up to 22 %. Pebbles of up to 3 cm clearly indicate fluvial sedimentation processes. However, an intense palaeosoil with a reddish ochre-brown color, and a higher Fe(d)/Fe(t)-ratio, as well as higher clay contents (~ 22 %) may correspond to the palaeosoil usually found in SU-4. But since the upper connection to the profile is missing, we cannot state that with certainty. In the uppermost layer of this lower

part of the profile, coarse silt contents and the GSI strongly increase that suggests the incorporation of loess. Unfortunately, the profile became inaccessible here, so we had to switch to another exposure 60 m to the north.

The upper part of the profile is characterized by permanent sand contents between 16 and 52 %. Usually, the maximum is in fine sands, but occasionally medium sand reaches up to 25 %. However, most layers reveal features indicating a primal aeolian accumulation, although in the upper part dispersed small pebbles indicate the contribution of slope processes or probably extreme storm events. As the section is situated very close to the recent floodplain, both in vertical and horizontal distance, we assume that the sands may have been blown by wind over these short distances.

At the base of the upper part, directly above the gravels (SU-2), we found a palaeosoil with a weak reddish-ochre color in a pale ochre sediment (SU-7). This palaeosoil could not be confirmed by analytical data, thus we assume that the coloring was caused by long lasting surface exposure that led to a first decalcification under environmental conditions that hampered further weathering processes. OSL dating results show that the sediments of SU-7 were deposited at about 28.4 ± 2.4 ka, and that the incipient soil was exposed until 24.7 ± 2.0 ka. The following 2 m belong to SU-8, whereas the first meter reveals an alternate layering of reddish-ochre sandy material, and pale grayish-ochre more silty sediments. Afterwards, an ochre-brown material rich in silt and with high GSI values of more than 2.5 is following. This layer ends up with a darker and more reddish horizon showing features of weak soil aggregation. However, these features belong to a just short period of surface stability as indicated by OSL dating that suggests a relatively fast successive sedimentation of SU-8 between 24.7 ± 2.0 and 23.2 ± 1.6 ka.

Finally, the section is covered by a further loess-like sediment showing relocation features (SU-9), and a colluvial deposit that most probably belongs to the Holocene period.

SI Text 5

Luminescence dating results

Luminescence properties. Except for the youngest samples BT 1375 and BT 1544, all investigated luminescence samples showed bright and fast decaying shine down curves. Due to the low equivalent doses accumulated during the very short period elapsed since deposition, BT 1375 and 1544 showed fast decaying but dim shine down curves. Typical OSL shine down curves for both, bright and dim samples, are shown in Fig. S7. Indicated by OSL IR depletion ratios very close to unity and confirmed by the investigated IRSL shine down curves, no prominent IRSL signal was detectable and, thus, a significant contamination with feldspar remnants could be excluded. Recuperation was always close

to zero and therefore below the critical threshold of 5% proving the absence of any significant thermal transfer of charge from light-insensitive traps to luminescence traps during the measurement procedure (1). Recycling ratios for most aliquots (> 97%) were within the generally accepted range of 0.9 to 1.1, indicating an excellent performance of the applied SAR-protocol (see Fig. S8, Table S3). The results derived from the dose recovery tests confirm the good performance of the applied SAR-protocol and prove the appropriateness of the used sample material. Fig. S9 depicts examples of dose recovery tests conducted for four different samples. For all samples and all preheat temperatures, the mean values of the measured-to-given-dose ratio are within 10% of unity. Although the DRR for sample BT 1365 seems to reveal a slight trend with preheat temperature, this proved not to be statistically significant. Over all, a good average dose recovery could be determined for all samples.

Equivalent dose distribution and luminescence ages. Fig. S10 shows dose response curves and abanico plots of equivalent dose distributions (2) for a selection of different samples. For all investigated samples dose response curves were fitted using a single saturating exponential function. For the majority of samples dose response curve construction was possible without any serious problems indicated by low average fitting errors and yielding equivalent doses in the exponential but still growing part of the curve (Fig. S10 – upper part). However, for each investigated profile the oldest samples proved to be problematic (e.g. BT 1367 and BT 1368 for the Fuentidueña section, BT 1372 and BT 1374 for the A3 section as well as BT 1383 and BT 1384 for the Paraíso section). For quite a substantial number of aliquots measured for these samples the natural signal (Ln/Tn) was in saturation and, thus, no equivalent doses could be derived at all (Fig. S10 – lower part). For other aliquots it was at least possible to determine equivalent doses, but these doses often were in the saturating part of the respective dose response curve, showing large palaeodose errors and frequently exceeding the $2D_0$-criterion described above. For these problematic samples a large number of aliquots had to be dismissed, e.g. for sample BT 1368 only 9 out of 24 measured aliquots could be considered for equivalent dose determination after applying the rejection criteria (for details the reader is referred to Table S2).

When looking at the equivalent dose distributions, the non-problematic samples in general are characterized by narrow and tight, often slightly skewed Gaussian like distributions. The typical relative standard deviation is in a range of 5-6% with slightly higher values of 8-9% for the middle part of the Paraíso section. The problematic samples show much wider kernel density estimate (KDE) plots accompanied by relative standard deviations of 13% for sample BT 1368 up to more than 20% for BT 1372 and BT 1374. The calculated mean equivalent doses are well beyond 200Gy; for sample BT 1372 and BT 1374 even close to 300Gy. These equivalent doses are in a dose range area that is

regularly regarded to be close to the saturation level for typical quartz samples (3-5). Hence, samples BT 1367, BT 1368, BT 1372, BT 1374, BT 1383, BT 1384, and HUB 470 (Fig. S11) might most probably suffer from distinct saturation effects. Therefore, we clearly have to point out that the ages calculated for these problematic samples should be taken with care and can only be interpreted as minimum ages! They can give not more than a rough guess of the time when the sedimentation process might at least have taken place. However, we cannot preclude a much earlier deposition of the respective sediment layers. For the non-problematic samples, on the other hand, excellent luminescence properties could be determined and, therefore, we can emphasize that the age information derived from these non-problematic samples can be considered as highly reliable. The calculated luminescence ages, including the analytic data for OSL age calculation along with the data for dose rate determination are given in Tables S1 and S2.

SI Text 6

Radiocarbon dating of n-alkane biomarkers

For radiocarbon dating of *n*-alkane biomarkers, each sample was extracted three times using accelerating solvent extraction (ASE, Dionex 200, 6.9 MPa, 100°C, three extraction cycles) with DCM:MeOH 9:1 (v:v). Extracts were combined and separated over aminopropyl silica (Supelco, 45µm) pipette columns. The aliphatic fraction including the *n*-alkanes was eluted with *n*-hexane. Radiocarbon dating was carried out on the *n*-alkanes as a whole compound class. Prior to radiocarbon measurements, *n*-alkanes were cleaned over coupled silver-nitrate (AgNO3-) - zeolite pipette columns to increase purity. The *n*-alkanes were trapped in the zeolite and subsequently dissolved in hydrofluoric acid and recovered by liquid-liquid extraction with hexane. Afterwards, purified *n*-alkanes were transferred with DCM into tin capsules (3.5 * 5.5 * 0.1 mm). Radiocarbon measurements were performed on the MICADAS AMS coupled online to an EA unit at the LARA AMS Laboratory, University of Bern. Results are reported as fraction modern carbon ($F^{14}C$), normalized to the reference material (Oxalic Acid II, National Institute of Standards and Technology) after subtracting the background signal. All $F^{14}C$ values were corrected for cross and constant contamination. Radiocarbon ages were calibrated using OxCal and the IntCal13 calibration curve.
The bulk ^{14}C age of the *n*-alkane sample PA 39 ranges from 26.6 to 28.8 ka cal. BP (Table S4) and is consistent with the corresponding OSL age from the same stratigraphic position. Samples PA 54 and PA 52 on the other hand, are derived from Holocene colluvial deposits, while the upper one shows an older age compared to the OSL results. Moreover, the ^{14}C ages of those samples are stratigraphically inverse and might represent a “pre-aged” component since they have undergone pedogenesis before erosion and final deposition. This might explain the age offset between the older ^{14}C ages and the OSL ages.

The ^{14}C age of the samples PA 50 and PA 46 also show an offset with the OSL dating results in the respective loess layers with much younger ^{14}C ages (~15 ka) compared to the corresponding OSL ages. However, those *n*-alkanes have greater amounts of short-chain *n*-alkanes (C_{17}-C_{25}) without an odd-over-even predominance, whereas the shorter chain-lengths are thought to indicate microbial activity (6). Thus, microbial produced *n*-alkanes will contribute to the ^{14}C age of the leaf wax *n*-alkanes with much younger ages and can explain the offset to the OSL ages. To overcome the disadvantage of microbial contamination, compound-specific ^{14}C dating of the longer leaf wax derived *n*-alkanes *n*-C_{27} – *n*-C_{33} is necessary and might deliver more accurate ages (7). However, compound-specific ^{14}C-dating is cost and time consuming and not always possible since high concentration of the individual compounds are needed.

SI Text 7

Micromorphological observations

The micromorphological analysis of Paraíso thin sections (see Fig. S12 for sampling points) shows an uniformity in the groundmass of the material, which consists of well sorted silt with minor amounts of sand grains. The apedal material is characterized by a channel microstructure and a calcitic crystallitic b-fabric (Figs. S13a and S13b), due to micrite as a product of weathering of the sediment rich in calcite (8). The soil formation processes are minimal, solely consisting of bioturbation and carbonate/gypsum redistribution.

Six main types of calcite enrichment or depletion pedofeatures and two types of gypsum pedofeatures are present in the Paraíso thin sections:

- MD (matrix depletion): depletion of calcite with enrichment of clay and iron oxides partly enveloping mineral grains (Figs. S13c to S13f)
- DH (depletion hypocoating): depletion of calcite around a channel (Figs. S14a and S14b)
- DH-CI (compound depletion hypocoating and calcitic infilling): depletion of calcite around a channel in combination with a calcitic infilling of the channel (micrite, microsparite, sparite) (Figs. S14c and S14d)
- CC/CI (calcitic coating/ calcitic infilling): calcitic crystals as coatings or infillings of a void (channels, packing voids) (Figs. S14e and S14f)
- CH (calcitic hypocoating): enrichment of calcite in the matrix around a channel (Figs. S15a and S15b)
- CH-DI (compound calcitic hypocoating and depletion infilling): compound pedofeature of calcite enrichment in the matrix around a channel and decalcified infilling of a channel (Figs. S15c and S15d)

- GC/GI (gypsum coating/gypsum infilling): gypsum crystals as coatings or infillings of a void (channel) (Figs. S15e, S15f, S16a and S16b)
- DH-GC (compound depletion hypocoating and gypsum coating): depletion of calcite around a channel in combination with a gypsum coating of the channel (Figs. S16c and S16d)

The distribution of these features throughout the Paraíso sequence can be taken from Table S5 as a basis for following descriptions. The sequence can be divided into three parts: The lowest part comprising thin sections Pa1 to Pa4 is characterized by both depletion and precipitation pedo-features. Despite the dominance of micritic impregnation of the material in Pa1 a weak dissolution of carbonate has taken place resulting in a formation of residual clay partly enveloping mineral grains without a complete decalcification of the material (MD). The calcitic crystallitic b-fabric of the sediment was not impaired by this process. Decalcification can be observed along some biogenic voids forming depletion hypocoatings (DH) with a speckled b-fabric (Pa1, Pa3, Pa4). These features are formed by removal of calcite in solution (9). The decalcification tendency that can be observed in Pa1 indicates the material of the stratigraphic unit 4 (SU-4) has experienced some moist environmental conditions allowing weak carbonate dissolution subsequently following its sedimentation. Thin section Pa2 is lacking depletion pedofeatures but shows carbonate enrichment in the form of calcitic hypocoatings (CH) of voids and a strong micrite impregnation of the matrix. There are two main hypotheses concerning the formation of calcitic hypocoatings (10, 11). (i) They are formed from soil solutions percolating along the pores and penetrating into the matrix (9, 12). (ii) They represent fast accumulations in connection with root metabolism (water suction and desiccating effect (13)). Generally, calcitic hypocoatings are forming under arid and semiarid conditions and a patchy vegetation cover probably already during the loess accumulation (11). Although hypocoatings indicate dry formation conditions, some percolation during moister conditions (at least periodically occurring) must have taken place during their development (10). The characteristics of the thin sections Pa2 and Pa3 representing stratigraphic unit 5 (SU-5) are showing a contrary tendency of calcite pedofeatures. While in Pa3 depletion pedofeatures are dominating, Pa2 is characterized by strong accumulation of calcite. The combination of these features can be taken as evidence for local redistribution of carbonate as a result of soil formation affecting the material of stratigraphic unit 5 (SU-5). Thin section Pa4 (partly representing stratigraphic unit 6 (SU-6)) is showing both carbonate dissolution (depletion hypocoatings) in its upper part and accumulation (calcitic infillings) in its lower part and therefore can be integrated into the same tendency of pedogenesis. Occurrence and distribution of carbonate dissolution and accumulation features indicate the strongest phases of pedogenesis for the lower part of the Paraíso sequence. Soil formation in terms of partly decalcification or redistribution of carbonate are visible.

The second part of the Paraíso section (Pa5, Pa6, Pa7) is mainly characterized by strong bioturbation and the common appearance of compound calcitic hypocoatings and depletion infillings (CH-DI). This compound pedofeature is described from a loess-palaeosoil sequence in China (12), where two possible hypotheses for their formation are discussed: (i) calcitic hypocoatings around channels that were later infilled with decalcified material from overlying horizons and (ii) excremental infillings that were decalcified and the solutes were reprecipitated as micritic hypocoatings. In consequence of the lack of decalcified material in the overlying strata and a source for the secondary carbonate (hypocoating) the second formation hypothesis is privileged (12). The CH-DI pedofeature is most strongly developed in Pa5 (the lowest part of this section in Paraíso sequence). The overlying material (thin sections Pa8, Pa9, Pa10) are not considerably effected by decalcification and therefore not a source for material free from carbonate. Moreover, the amount of material washed in from overlying horizons should decline with depth. Here the reverse is true. Consequently, our interpretation follows the second formation hypothesis of a decalcification of channel infillings and subsequent precipitation of micrite as hypocoating in the adjacent matrix (12). In the lower part of the stratigraphic unit 7 (SU-7) represented by the thin sections Pa6 and Pa7 soil formation processes can be characterized by strong bioturbation and redistribution of carbonate restricted to larger biogenic voids facilitating preferential flow of percolating water.

The third and uppermost part of the Paraíso sequence comprising stratigraphic units 7 and 8 (SU-7 and SU-8, represented by the thin sections Pa8, Pa9, Pa10) shows that with accretion of the material the intensity of soil formation declines. The matrix contains large amounts of micrite and pedofeatures characterizing carbonate redistribution processes are absent or rarely appear.

The lower part of the Paraíso sequence (Pa1 to Pa6) contains accumulations of gypsum appearing as infillings and coatings of voids. The crystals are predominantly tabular or lenticular shaped and poorly sorted (fine to sand sized). Since the gypsum crystals are accumulated in pores they are regarded to be clearly pedogenic (8, 14). Some of the coatings forming a ring of gypsum crystals arranged around a central opening are containing remains of decomposed roots (Figs. S16e and S16f). Gypsum coatings with a change in crystal size and morphology with distance from the root are considered to be formed by direct precipitation processes (15). On the other hand, coatings in pores showing lenticular shaped crystals are assumed to be formed without the disturbance of roots (14). The gypsum coatings found in the thin sections of Paraíso cannot be clearly assigned to one of the two formation types since coatings containing root remains are consisting of only one row of crystals and coatings without roots are not necessarily formed of crystal with lenticular shapes. The occurrence of secondary gypsum as

coatings and infillings of voids in lower parts of the loess sequences indicates that the gypsum crystals are not part of the aeolian sediment but can be interpreted as leached from the gypsum-containing on-site substrates (16).

SI Text 8

Heavy mineralogical observations

Geological context. The study area is situated within the upper Tagus catchment (Fig. 1), but regarding potential source areas of loess it is also important to consider the catchments of the Jarama River north of the loess sections and the Algodor River that is located in the southwest (Fig. S1). Principally, loess can be found on deeply incised areas linked to the Tagus river system between Aranjuez and Rio Llano north of Tarancón. Here, the Tagus incised into Upper Miocene and Pliocene marls, marly clays and limestones, and Middle Miocene gypsum and gypsum marls that have been evaporated in the former center of a large closed depression. In the transition zone between the depression and the framing mountain ranges, the Tagus and its tributaries cut into detritic arkosic sediments. In the northernmost catchment, the Jarama and its tributaries Manzanares and Henares drain the granitic and metamorphic rocks of the Sierra de Guadarrama, the easternmost part of the Spanish Central System. Granites and granite gneiss likewise occur in the Montes de Toledo, a mountain range that is located south of the Tagus River in the catchment of the Algodor River. The east of the catchment that is drained by the Tagus and the Tajuña Rivers, belongs to the northwest-southeast directed Iberian Range and consists mainly of marine limestone and dolomite from the Jurassic and Cretaceous, and feldspar-rich sandstones from the Triassic. On its way through the Upper Tagus Basin, the Tagus River passes the Sierra de Altomira, a small mountain range that constitutes limestone, dolomite, and marls from the Cretaceous. These main geological units, including the valleys of the rivers that drain them, provide the main sources of the Tagus loess deposits. This already indicates that gypsum marls and marly clays appear in the immediate vicinity of the main loess sections, whereas granites and metamorphic rocks are limited to areas in a certain distance to the north and southwest. Finally, limestone and dolomites are mainly distributed in the east of the catchment that is drained by the Tagus River. These spatial patterns are emphasized, since they have special importance for the provenience of mineral spectra found in loess.

Reference samples. The general aim of this methodological approach was to determine the main proveniences of the Tagus loess. Therefore, we have chosen specific reference samples according to the main geological units surrounding the study area (see chap. 'Geological context'). A first indication of one possible source of the loess was already provided by texture analyses. Since the loess sections reveal fine sand contents of at least 10 % up to 30 %, it is conceivable that a main component has been blown out of the Tagus River floodplain, which is located in the immediate vicinity. Thus, one reference

sample was taken from a sandy to loamy Tagus river terrace of an assumed Late Pleistocene age (sample 'Tajo 1', see Table S6). Moreover, this sample serves as a reference for the Mesozoic sedimentary rocks of the Iberian Range and the Sierra de Altomira that are both located further east and that are mainly drained by the upper Tagus River. Further west and north of the study area, the large mountain ranges are mainly composed of granitic and metamorphic Palaeozoic rocks, such as the Sierra de Gredos, Sierra de Guadarrama, and the Montes de Toledo. In order to cover the mineralogical signature that is representative for these areas we sampled a sandy sediment body from the floodplain of the Algodor River that drains the main areas of the Montes de Toledo (sample 'Algodor 2'). A third option results from the hypothesis that during dry periods silt and sand-sized particles made up of marls have been blown out from the weathering mantle of the tertiary marls in the surroundings of the studied sections. Therefore, we likewise took a reference sample of these marls from the location of the Paraíso section (sample 'Miocene marl').

These reference samples reveal some individual heavy mineralogical characteristics that can be used to evaluate the main origin of the Tagus loess (see Table S6). In line with the typical mineral composition of Palaeozoic plutonic rocks and regional metamorphic rocks (17, 18), the fraction of transparent heavy minerals of the sample 'Algodor 2' shows a maximum in garnet and topaz. The sample 'Tajo 1' that represents sediments originating from the Iberian Range and the Sierra de Altomira reveals a maximum in tourmaline, but also rutile. Finally, the Tertiary marls should actually provide a mixed-signal from all the surrounding mountain ranges according to their genesis in a large depression, mainly during the Miocene. However, the respective sample shows a maximum in apatite, and it is the only sample that indicates the occurrence of small amounts of titanite and brookite.

Because of the good correlation in the ternary plot (Fig. S17) we selected following reference minerals for the interpretation of the loess samples. Palaeozoic granitic and metamorphic rocks are represented by garnet, Tertiary evaporitic marls are represented by apatite, and the Mesozoic sedimentary rocks are represented by tourmaline. However, since tourmaline is not so clearly assignable because of relatively high concentrations in the marls, as well as the Algodor sample, we added dolomite as a further reference mineral for the Mesozoic. Dolomite was not eliminated during sample pretreatment (see Methods) because of its resistance against acidic acid, and was captured during the heavy mineral separation because of its high density of 2.9 g/cm^3. Generally, dolomite can clearly be attributed to the area of Mesozoic sedimentary rocks, thus we depicted a second ternary diagram where tourmaline and dolomite were combined (Fig. S18). However, since in our case dolomite represents rather a main component than an accessory, it clearly dominates the heavy mineral distribution and thus has to be interpreted with caution.

Loess samples (Paraíso section). All reference samples plot close by the corners of the ternary diagram (Figs. S17 and S18), while the samples 'Miocene marl' and 'Algodor 2' are slightly shifted along the z-axis due to a certain amount of tourmaline within these samples. Tertiary marls nearby the Paraíso section show increased contents of apatite, titanite and mica, lower contents of dolomite and opaque minerals, and the unique occurrence of brookite and pyroxene group. Starting with the first sediment layer (SU-3), all loess units reveal the impact of foreign admixtures. Already sample 9 (SU-3) shows decreased apatite contents, but increased contents of tourmaline and garnet that indicates the incorporation of allochthonous material. The diagram in Fig. S17 shows a strong concentration of several loess samples nearby the center of the reference samples with a slight shift to the corner representing Tagus floodplain sediments. That might indicate a homogeneous mixture of all three components with a somehow greater influence of the Tagus floodplain as sediment source.

The diagram in Fig. S18 that includes dolomite as a reference for the Tagus floodplain reveals a much stronger shift of some loess samples along the z-axis, which is not surprising in view of dolomite contents of even more than 95%. A clear cluster is formed by samples belonging to units SU-3, SU-4 and SU-10 with a shift towards garnet and apatite. This result fits well with a stronger impact of slope processes that affected these units, which is likewise supported by field evidences. SU-7 and SU-8 are clearly dominated by dolomite suggesting that the Tagus floodplain was the main sediment source during the upper part of the last glacial.

Finally, SU-6 (sample no. 6) occupies a particular position. In Fig. S16, it is closest to the 'Miocene marl' sample and shows less influence of the Tagus floodplain. However, in Fig. S18 the influence of the Tagus floodplain increases, but the content of 57% of dolomite is still much lower compared to the samples of the upper profile section. From this, we conclude that the Tagus floodplain was probably not the dominant sediment source during the deposition of SU-6. Apparently, local marls but also granitic and metamorphic rocks from mountainous areas and river floodplains located further west had a greater importance regarding the supply of loess material, which points to a changed mode of loess generation and thus, may indicate somehow modified environmental conditions.

SI Text 9

The Late Pleistocene human occupation of interior Iberia

Until the beginnings of the current decade, Late Pleistocene population dynamics in interior Iberia seemed fairly well established. A late survival of Middle Palaeolithic contexts and associated Neanderthal groups was mostly accepted for these territories until

at least *circa* 36 ka cal. BP, and hence a prolonged period of coexistence with Modern Humans, established in Northern Iberia at c. 40-42 ka cal. BP, was proposed at the peninsular level (19-21). This late Neanderthal survival was thought to be followed by a population breakdown that caused a total depopulation of the Iberian interior until the end of the Last Glacial Maximum (22, 23). However, given the sparse geoarchaeological and chronometric record traditionally available in the inland territories of Iberia, evidence supporting these models has always been weak. Consequently, a handful of new chronometric data obtained in the last five years has significantly modified the paradigmatic view.

Application of new chronometric techniques to previously dated sites supporting a late Neanderthal survival in interior Iberia, mainly limited to La Ermita cave (Burgos) and Jarama VI rock shelter (Guadalajara), has shown a very different picture. At La Ermita, dates obtained by Aminoacid Racemization and Uranium/Thorium techniques have reassigned level 5a, previously radiocarbon dated in the range of *c*. 36.6 – 34.7 ka cal. BP, to MIS 5 (24). At Jarama VI, evidence supporting a late Neandertal persistence was shown by two radiocarbon dates placing the latest Mousterian of this site (level JVI.2.1.) between *c*. 30 and 41 ka cal. BP (25). However, new chronometric analyses, including radiocarbon measurements of bone samples pre-treated with ultrafiltration (26, 27) and luminescence dating (post-IR IRSL) of associated sediments (28), consistently places the Mousterian occupation of Jarama VI between *c*. 50 and 60 ka. All evidence supporting a late Neanderthal survival in inland Iberia has been thus refuted.

In consonance with these results, all other chronometric evidence obtained in the last years suggests that no Neanderthal presence was evident in interior Iberia later than 42 ka cal. BP. In the recently discovered rock shelter of El Molino (Segovia) (29), a series of radiocarbon and luminescence dates places the last Mousterian occupation of the site between *c*. 44 and 42 ka cal. BP (30). Similar ages have been obtained at Los Casares cave (Guadalajara), where both U/Th and radiocarbon measurements show that Neanderthal use of this cave took place between c. 45 and 42 ka cal. BP (31). Other inland Middle Palaeolithic sites having yielded reliable chronometric dates within MIS 3 are Prado Vargas (32), Hotel California (33), Valdegoba (34), La Mina (35) and Hundidero (36). None of them have provided any date younger than 42 ka cal. BP (see Table S7).

Yet, there are still three sites having yielded chronometric results younger than 42 ka cal. BP in the Iberian interior. However, all of them present important problems that prevent using these dates as compelling evidence for supporting a late Neanderthal survival. In Cueva Millán (Burgos), two radiocarbon measurements on bone were obtained in the 1980's, ranging from *c*. 43 to 40 ka cal. BP[35]. However, these dates were

obtained by the conventional radiocarbon method more than thirty years ago, and hence a new chronometric program aimed at contrasting these results is required before they can be considered as reliable. In the 12 de Octubre site (Madrid), a typical Mousterian assemblage is associated to a series of OSL dates between 40 and 33 ka. However, their excavators cast doubt on these results suggesting that ages, which contradict geomorphological data, are most probably underestimations (37). Lastly, in Cañaveral - Área 3 (Madrid), a TL date of 33 + 4.0/-3.5 ka was obtained at the top of a layer containing Levallois industries (38). However, besides the high standard deviation of this measurement, and that it must be considered a *terminus ante quem* for human activity, full discussion of methods and results of chronometric research conducted at this site is still to be published.

In short, at the current state of research, a Neanderthal persistence after 42 ka cal. BP cannot be accepted in the Iberian interior, but rather an abandonment of these territories was very likely around that date. Since current chronometric evidence supports a late presence of Neanderthals in the southern Iberian coasts until *c*. 37 ka cal BP (84), a parsimonious interpretation is that Neanderthal populations from the Iberian interior moved to the more favorable ecological niches of the coastlines around or shortly after 42 ka cal. BP, due to climatic stress or any other reason (31).

After this Neanderthal population breakdown, so far there is no evidence of a later reuse of the inner areas of Iberia until Gravettian times. No Aurignacian or so-called transitional industries have been documented in the Iberian interior (23, 39). The first occurrences of the Modern human occupation of these territories are found at the western fringe of the Meseta. In the Côa Valley (Duero basin, Portugal), two Gravettian layers have provided TL dates in the range of *c*. 31 - 26.5 ka at the sites of Olga Grande 4 (average of 28.7 ± 1.8 ka) and Cardina 1 (average of 27.9 ± 1.4 ka) (40, 41). Also in inland Portugal, at a northern area of the Douro basin, the site of Foz do Medal (Alto Sabor valley) has shown a fire structure associated to a scarce lithic assemblage, where two dates were obtained: a TL measurement of 25.1 +2.6/-2.3 ka and a radiocarbon date of 31.5 – 31.1. ka cal. BP (42). However, due to the contradictory results showed by these measurements and that associated lithic assemblage remains unpublished, these data cannot yet be considered useful.

In the northern fringe of the Meseta it is found the open-air site of Valle de las Orquídeas (Atapuerca), where two TL dates of 27.5 ± 2.3 ka and 29.9 ± 2.3 ka were obtained for a *terra rossa* layer below a level containing lithics typical of both Middle and Upper Palaeolithic technologies (43) (Table S8). Also at the Atapuerca area, in the site of Cueva Mayor, a sequence bearing few lithic and faunal remains (including some blades) was radiocarbon dated between *c*. 34 and 20 ka cal. BP (44). Finally, although fully located in

the southern foothills of the Baetic Mountains, and hence not within the Meseta, the southern site of El Palomar (Albacete) has shown two Gravettian assemblages radiocarbon dated to 31.1 – 30.3 ka cal. BP (level 4) and 30.9 – 29.9 ka cal. BP (level 5) (45).

All these evidences suggest that the first modern human occupation of the Iberian interior took place on the western and northern fringes of the Northern Meseta around c. 28 ka cal. BP, with a slightly earlier presence in at least the south-western vicinitiy of the plateau. Despite lithic raw materials mobility has been attested between the Côa Gravettian sites and inner areas of the Northern and Southern Mesetas (41, 46, 47), effective presence of human settlement in the very interior of Iberia is not found until *c*. 25.5 ka cal. BP. This is shown by a radiocarbon date on bone from a Gravettian layer at the site of Peña Capón (Guadalajara), a rock shelter located very close to the south-eastern foothills of the Central System Range (39, 48).

This spatio-temporal distribution of Gravettian sites suggests that resettlement of the interior areas of the Peninsula started in the peripheral areas of the Meseta and followed a progressive trend towards the center. In any case, Gravettian presence is still very sparse in Central Iberia, and it is only with the Solutrean when strong evidences of a more permanent settlement of the Iberian interior are found. This is shown by the growing number of Solutrean occurrences, including pre-Magdalenian rock art depictions, documented in central Iberia during the last years, especially around the Central System Range and the Madrid basin (48-50). This Gravettian and Solutrean presence at the very interior of the Iberian Peninsula , including a highly settled area in the Manzanares valley and a long occupational sequence at Peña Capón, contradicts previous models positing a depopulated or only sporadically visited Meseta until the retreat of the Last Glacial Maximum (39).

If during the last years the temporal scope of the Middle Palaeolithic in interior Iberia has been shortened for its youngest limit, an opposite process has occurred concerning the first phases of the Upper Palaeolithic. Thus, first evidences of modern human occupation are now recorded much earlier than previously thought, even in the central part of the peninsula. Yet, there is still a large population gap between the Middle and Upper Palaeolithic in the Iberian interior, which is currently recorded between c. 42 and 28 ka cal. BP, and must be extended until c. 25.5 ka in the case of the very interior territories (Fig. S20). Since inland Iberia should be still considered as an under-investigated area compared to the coastal regions of the Peninsula, especially concerning the Upper Palaeolithic (21, 23, 39, 46, 48, 51), it is important to bear in mind that this picture could change in the near future as ongoing research in a handful of sites will hopefully show new results (30, 51-55).

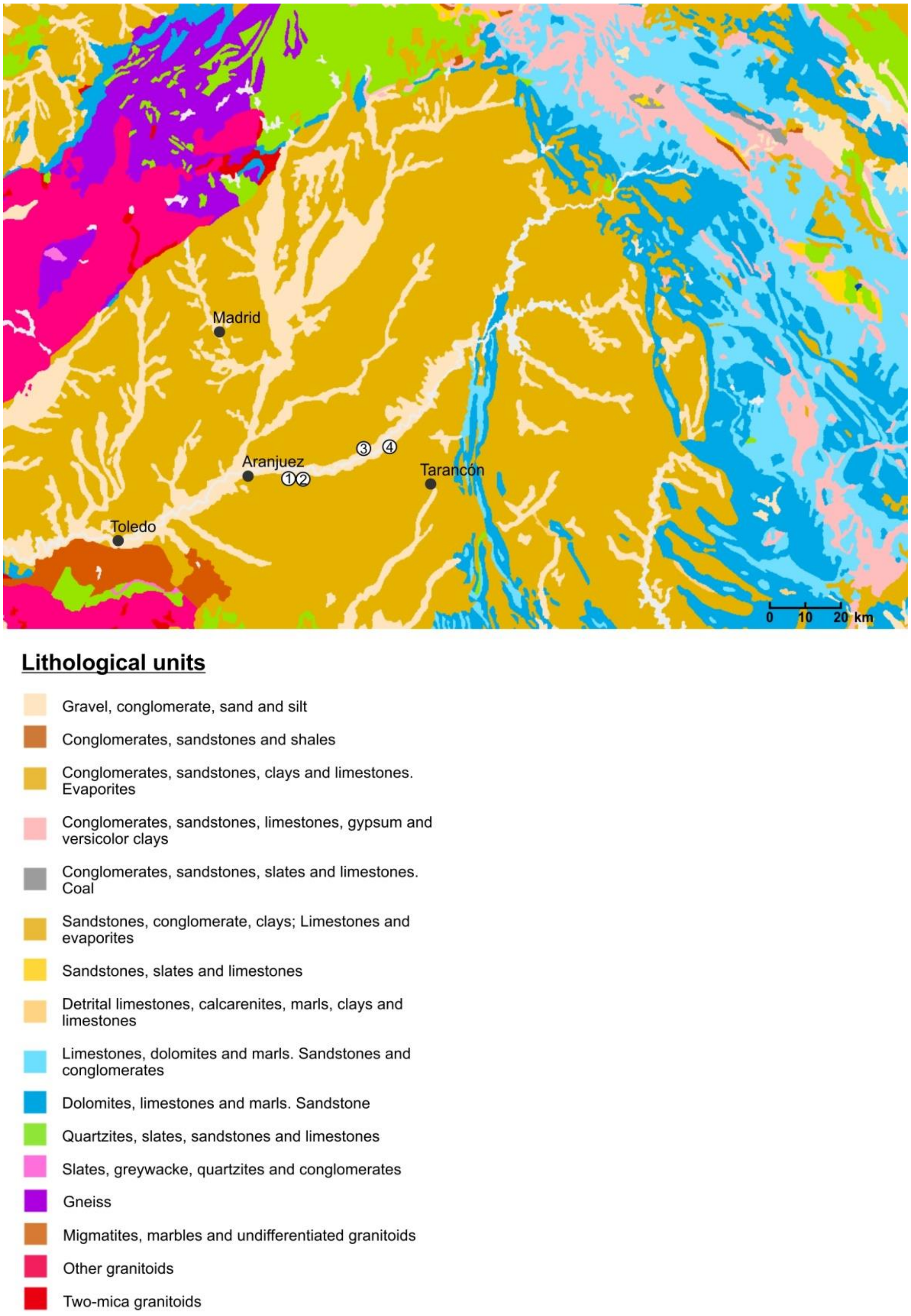

Fig. S1. Geological map and location of studied loess sections. (1) Profile Paraíso; (2) Profile Villarubia; (3) Profile Fuentidueña; (4) Profile A3. The map was generated using ArcGIS 10.2.2 (http://www.esri.com/software/arcgis) based on data from the Instituto Geológico y Minero de España (IGME)

(http://mapas.igme.es/gis/rest/services/Cartografia_Geologica/IGME_Litologias_1M/MapServer)

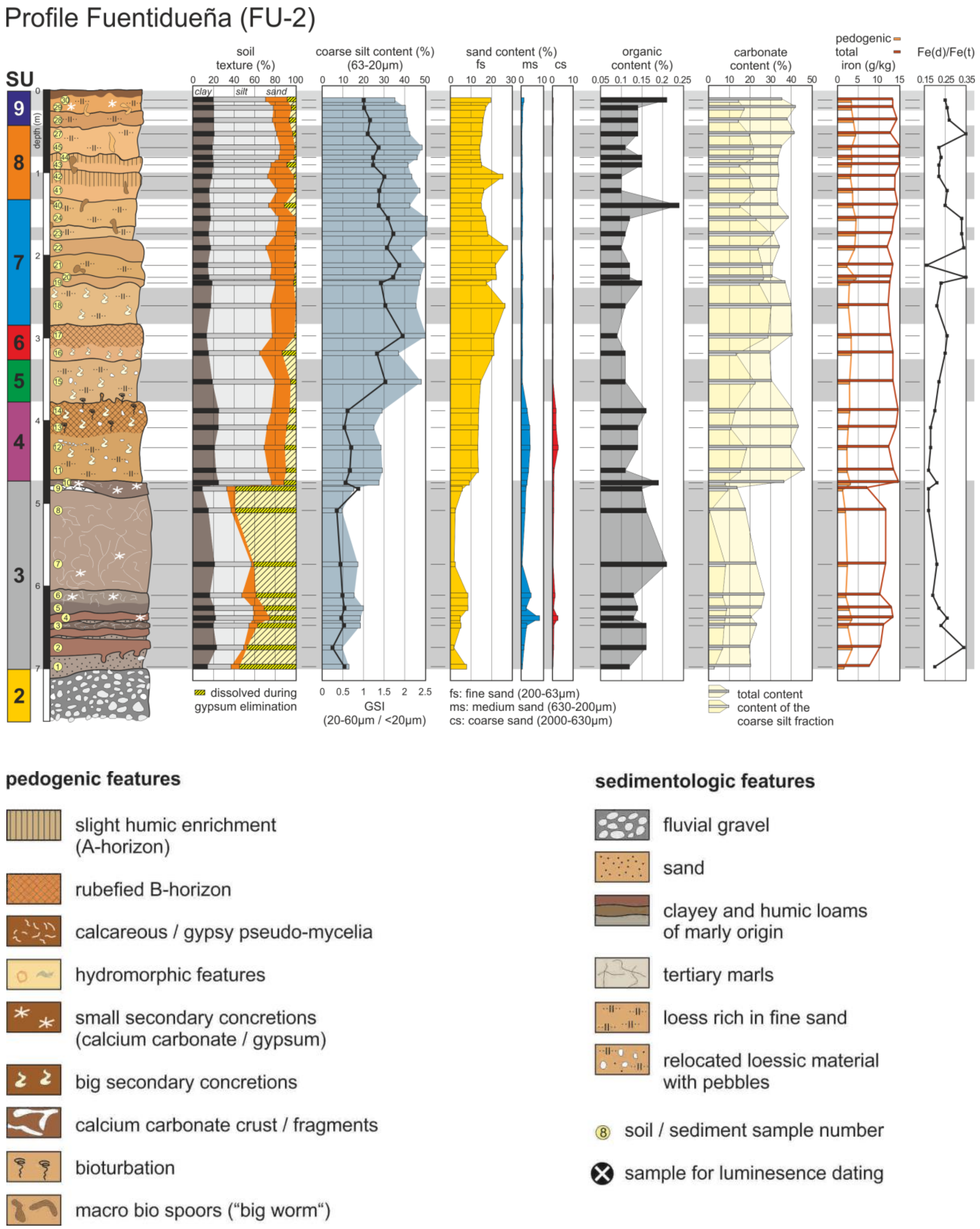

Fig. S2. Stratigraphic section of the Fuentidueña profile with indication of sediment units, sampling points, and analytical data, including grain size parameters, organic content, carbonate content, and the ratio between pedogenic and total iron as a weathering index. Please reference Fig. 2 for dating results.

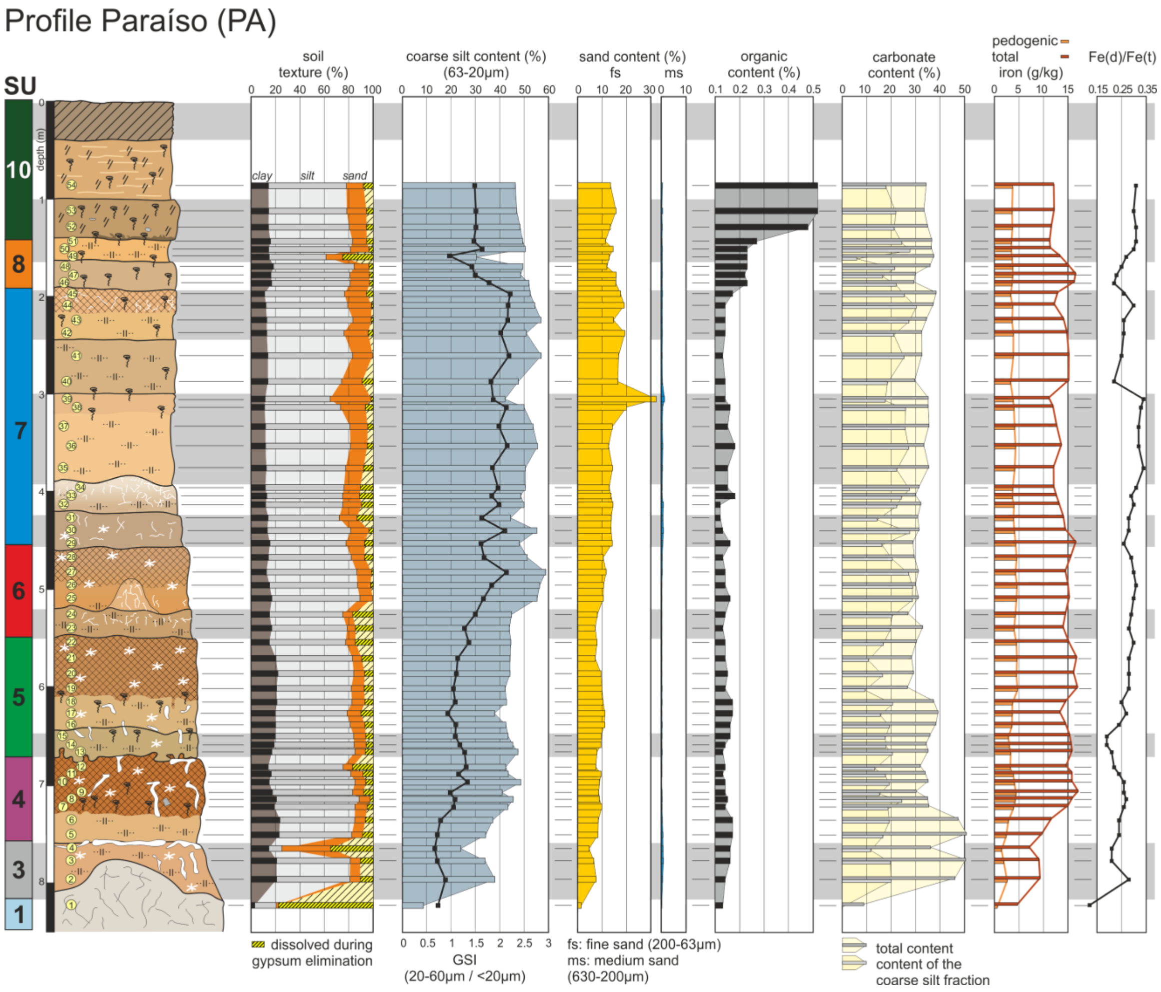

Fig. S3. Stratigraphic section of the Paraíso profile with indication of sediment units, sampling points, and analytical data, including grain size parameters, organic content, carbonate content, and the ratio between pedogenic and total iron as a weathering index. Please reference Fig. 2 for dating results, and Fig. S2 for legend.

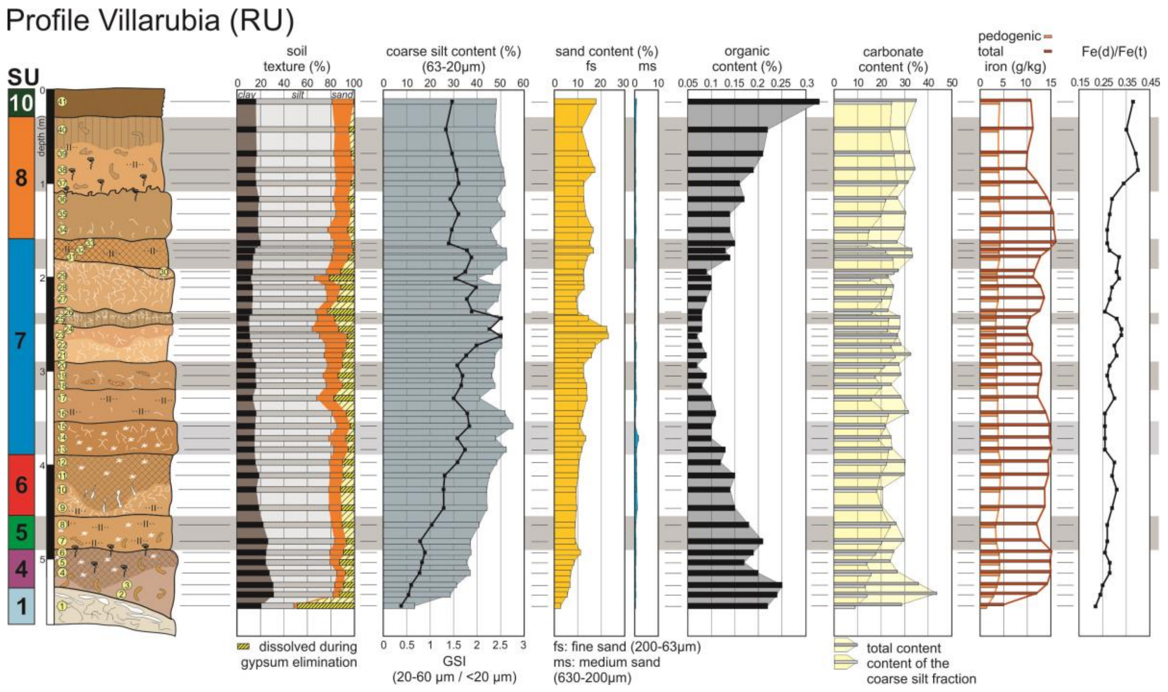

Fig. S4. Stratigraphic section of the Villarubia profile with indication of sediment units, sampling points, and analytical data, including grain size parameters, organic content, carbonate content, and the ratio between pedogenic and total iron as a weathering index. Please reference Fig. 2 for dating results, and Fig. S2 for legend.

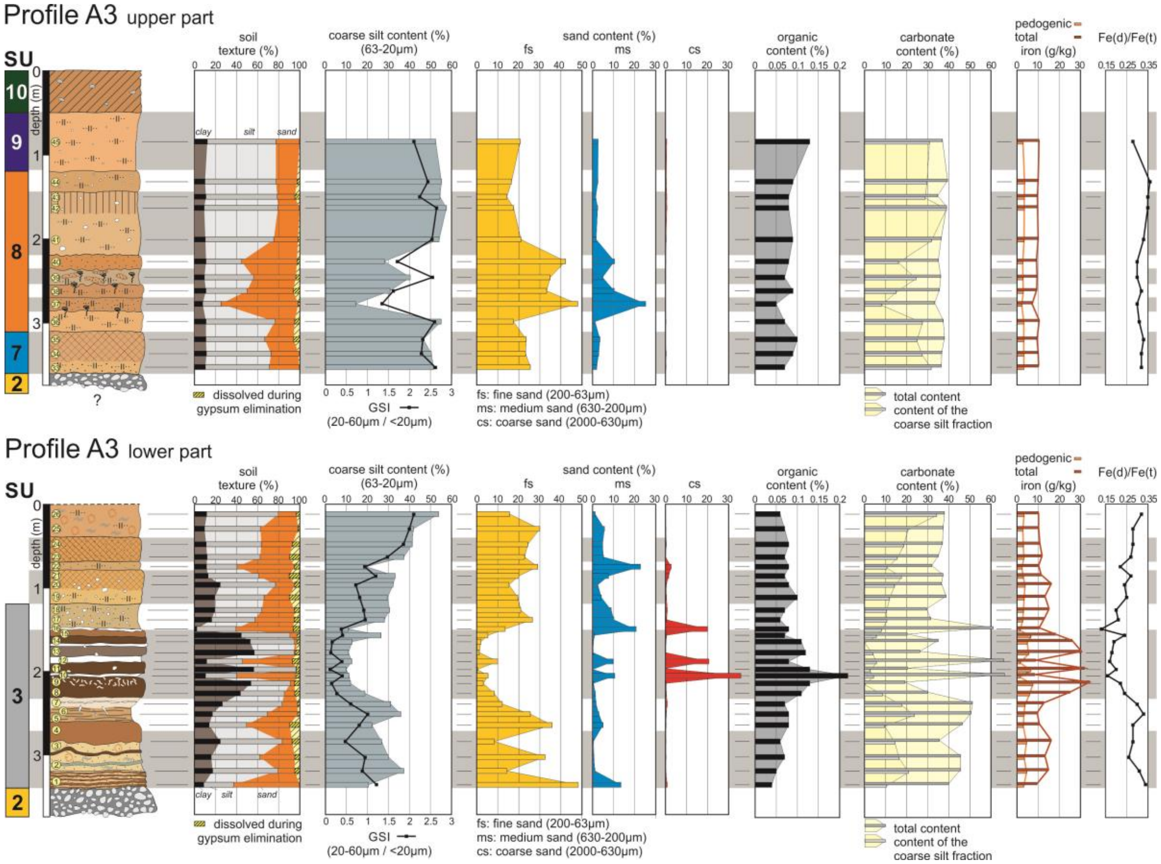

Fig. S5. Stratigraphic section of the upper and lower parts of the A3 profile with indication of sediment units, sampling points, and analytical data, including grain size parameters, organic content, carbonate content, and the ratio between pedogenic and total iron as a weathering index. Please reference Fig. 2 for dating results, and Fig. S2 for legend.

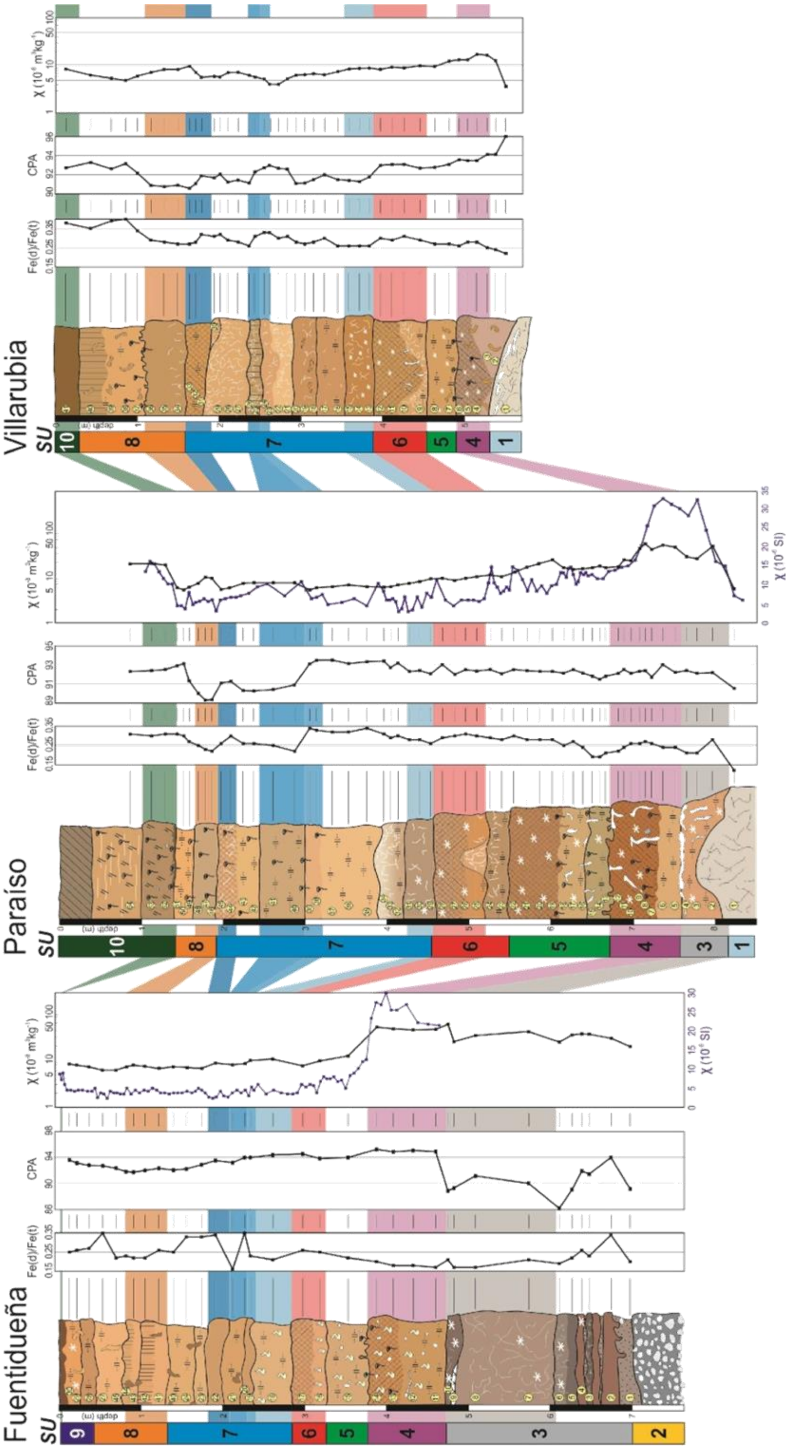

Fig. S6. Correlation between the loess sequences Fuentidueña, Paraíso, and Villarubia based on stratigraphic findings, the ratio between pedogenic and total iron, the 'Chemical Proxy of Alteration' (CPA) and the magnetic susceptibility. The CPA is based on the ratio between aluminum and sodium (56). The magnetic susceptibility is presented according to measurements in the field using a Bartington MS2 susceptibility meter and S2F probe in SI units (purple color), and as mass specific susceptibility χ that was measured in the laboratory (black color).

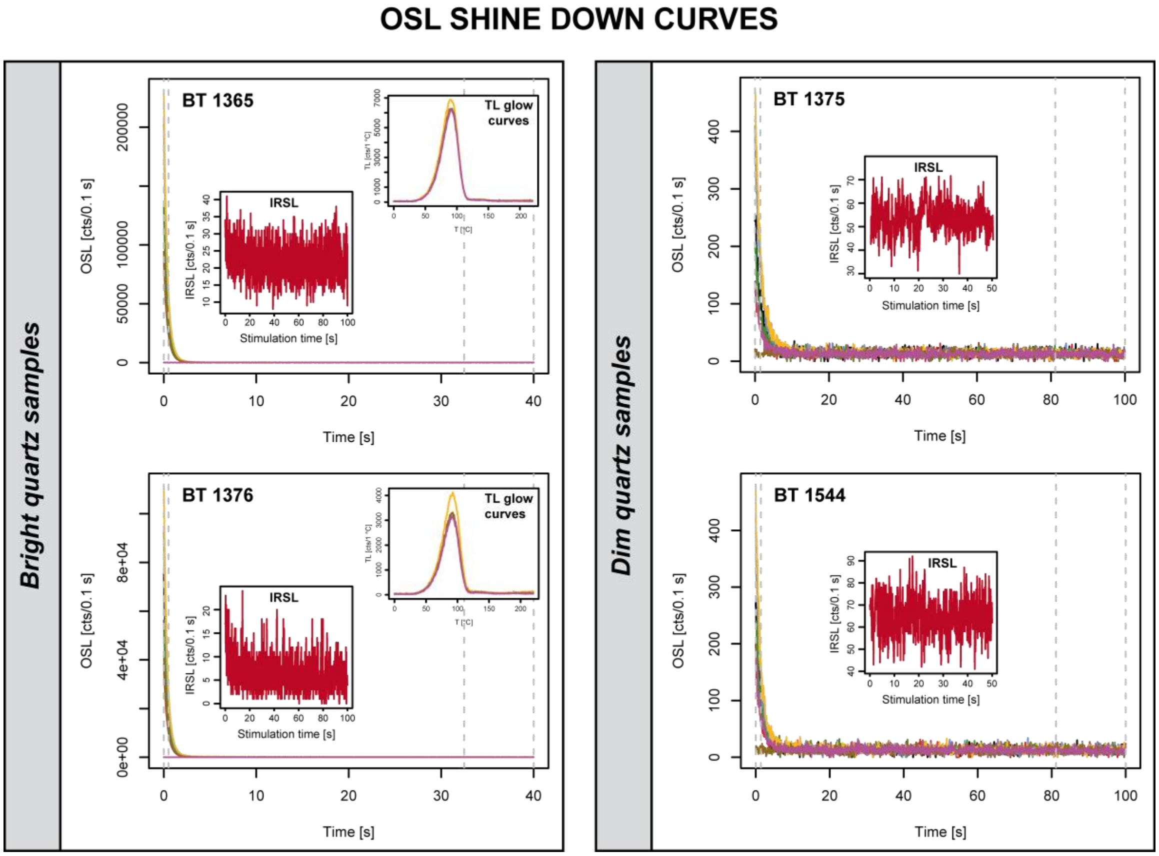

Fig. S7. Typical OSL shine down curves for bright quartz samples (left hand side) and dim quartz samples (right hand side). Whereas shine down curves for all other samples were recorded for a total of 40s, the measurement time for BT 1375 and BT 1544 was set to 100s. All OSL signals are characterized by a fast decay to a very low residual level. The insets show IRSL signals close to the measurement background indicating that there was no significant contribution of any feldspar remnants to the luminescence signal. For samples BT 1365 and BT 1376, also the recorded TL glow curves prior to the test dose measurement are depicted.

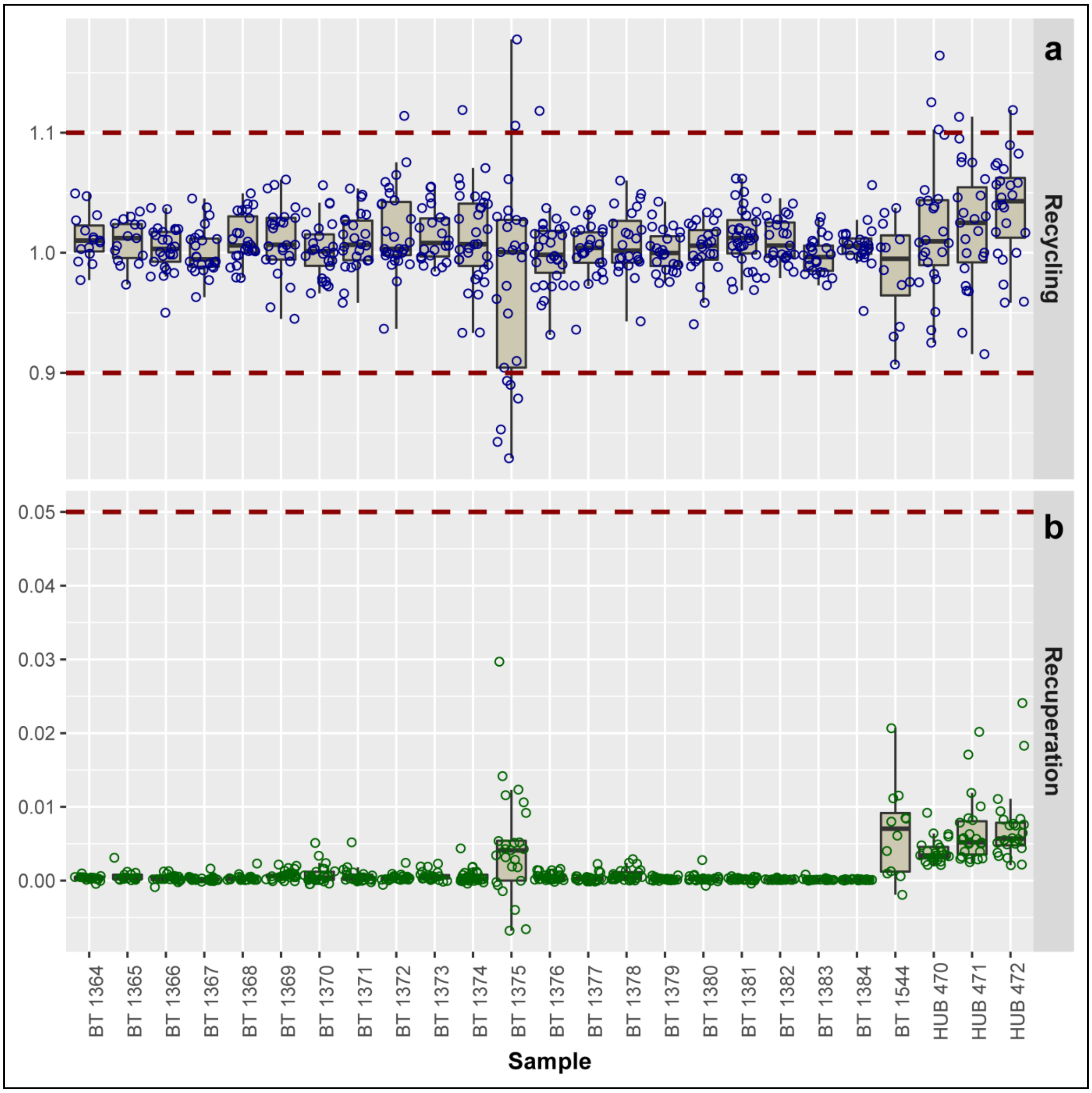

Fig. S8. Recycling ratios and recuperation. (*A*) Recycling ratios for all measured aliquots in this study. The dashed red lines indicate the threshold values typically applied as rejection criteria for luminescence measurements. 97% of all measured aliquots passed this criterion indicating the good performance of the used SAR protocol. (*B*) Recuperation for all investigated aliquots were below the critical value of 5%.

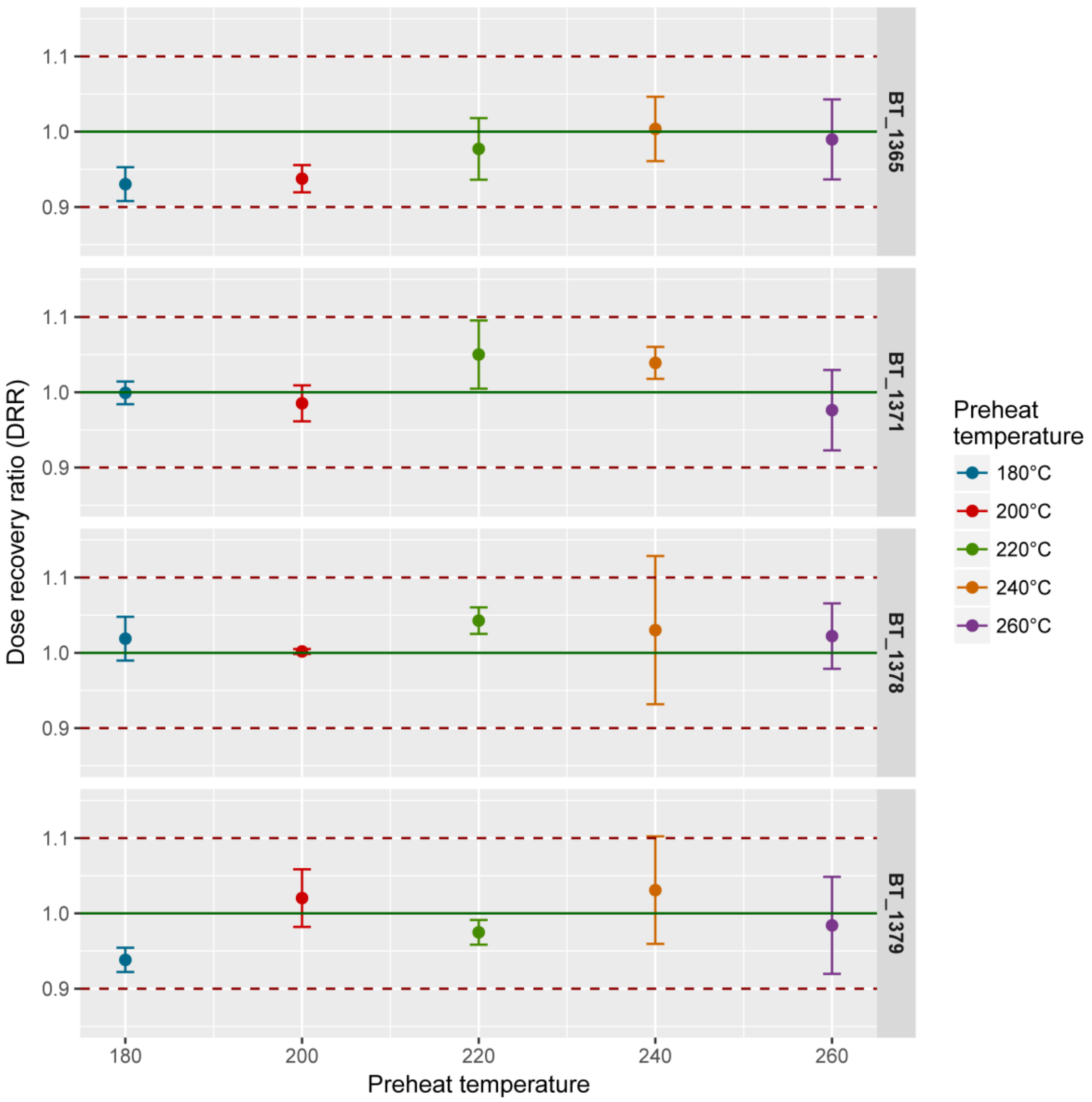

Fig. S9. Results from combined dose recovery and preheat tests for four different samples. While the green line indicates the known beta dose given to the sample prior to OSL measurement, the dashed red lines represent the threshold values for dose recovery. The y-axis shows the measured-to-given-dose ratio (dose recovery ratio). The various preheat temperatures are highlighted by different colors. The depicted data points represent the mean dose recovery ratio derived from three aliquots for each preheat temperature. Error bars indicate the 1σ-standard deviation. All investigated samples showed good dose recovery for all applied preheat temperatures. The optimal preheat temperature was individually determined for each sample.

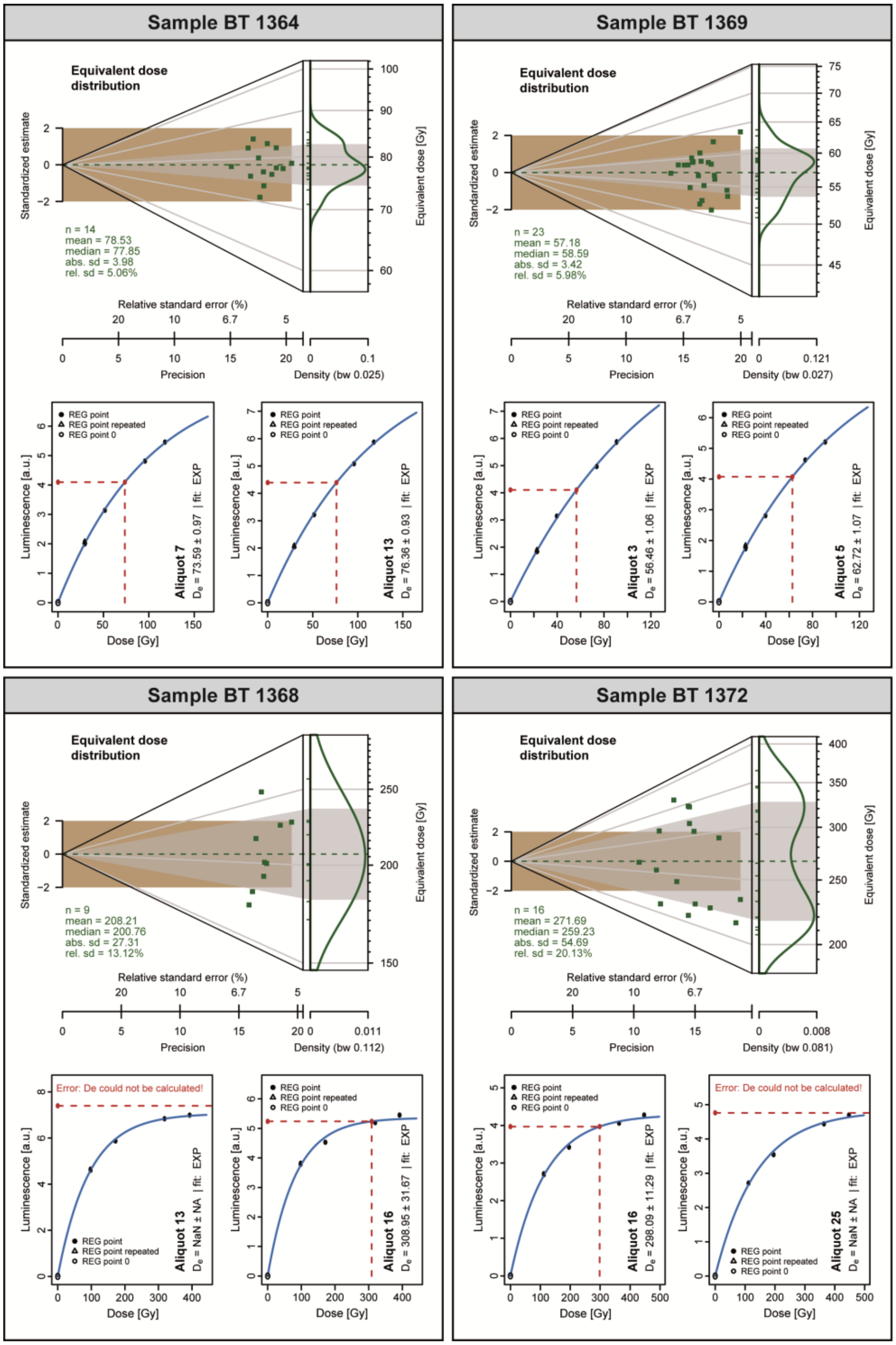

Fig. S10. Abanico plots of equivalent dose distributions. Each distribution shows two characteristic dose response curves for four different quartz samples. The greyish scatter polygons in the abanico plots illustrate the quartile range of the determined equivalent doses for the respective sample. The two samples in the upper part (BT 1364 and BT 1369) represent non-problematic samples characterized by narrow dose distributions. With BT 1368 and BT 1372 two examples for problematic samples are depicted in the lower part. They show a significantly higher scatter in equivalent dose distribution and a large number of aliquots that yielded dose response curves indicative of samples either in or close to saturation level.

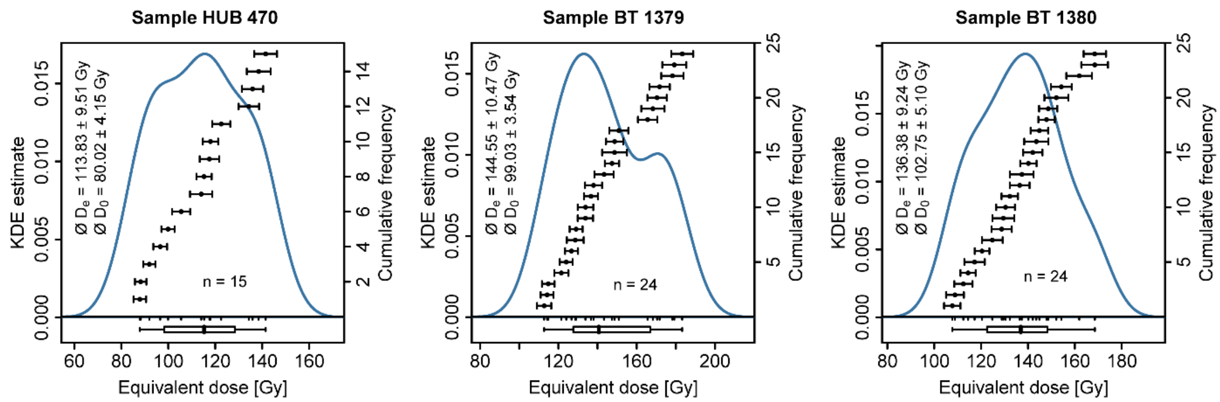

Fig. S11. Kernel density plots showing the equivalent dose distributions of three samples that revealed luminescence ages of ~ 40-42 ka. For all samples, the mean D_e and the average D_0 values are shown. While the mean D_e was derived only from those aliquots passing the rejection criteria, the average D_0 value was calculated as arithmetic mean of D_0 values of all aliquots for which dose response curve construction was possible. Please note that only for sample HUB 470 some aliquots had to be restricted due to supposed dose saturation (also see Table S2).

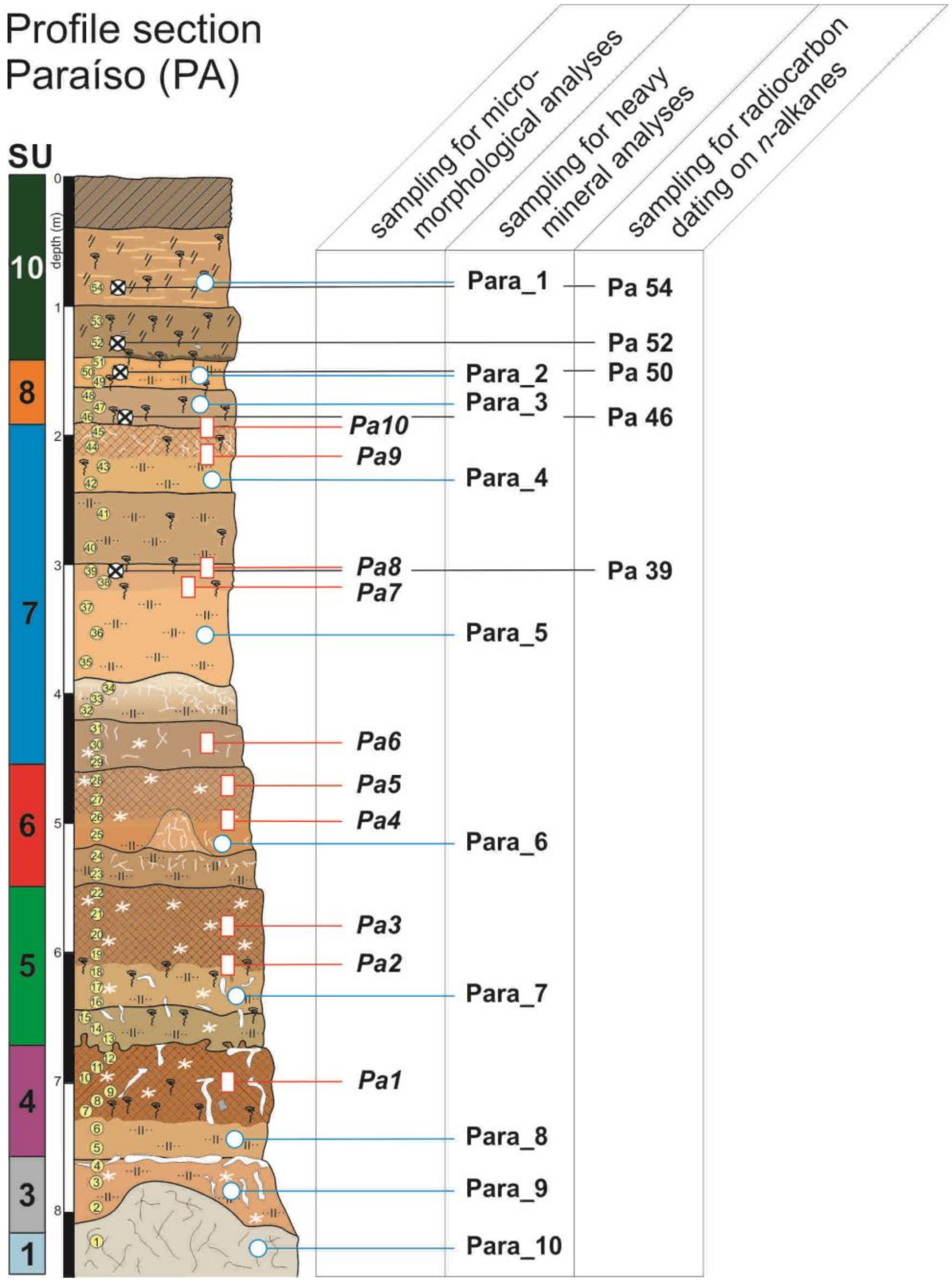

Fig. S12. Sampling points for micromorphological analyses (red squares), heavy mineral analyses (blue circles), and radiocarbon dating on *n*-alkanes (black circles) at the Paraíso section.

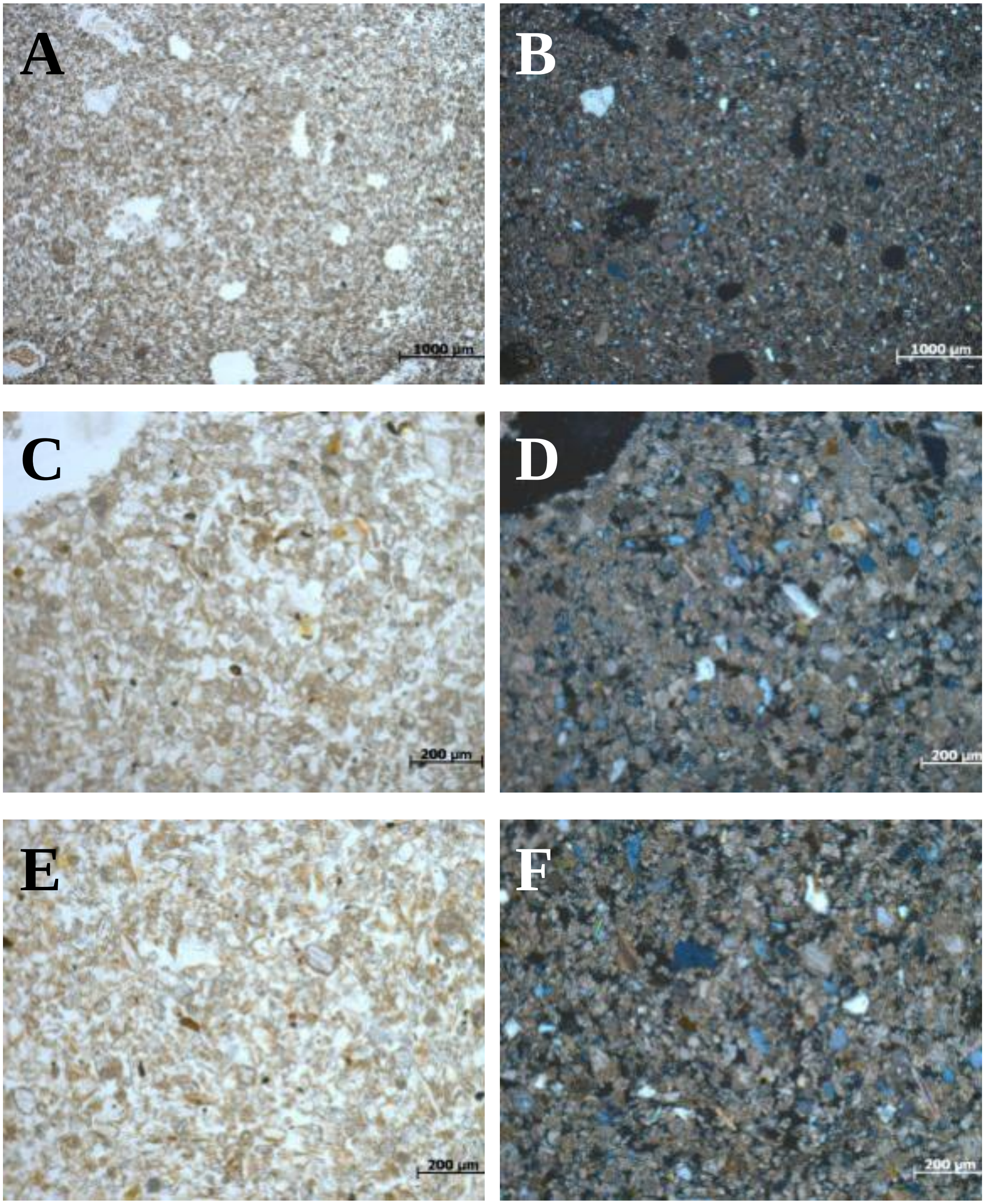

Fig. S13. (*A*) Micromorphology sample Pa3. Apedal material, channel microstructure, mainly silt-sized (ppl). (*B*) Same image detail like (*A*), calcitic crystallitic b-fabric, note the sand sized grain in the silty matrix (xpl). (*C*) Sample Pa2. Detail of the micritic groundmass (ppl). (*D*) Same image detail like (*C*), note the strong calcitic crystallitic b-fabric (xpl). (*E*) Sample Pa1. Weak depletion of calcite with enrichment of clay and iron oxides partly enveloping mineral grains (ppl). (*F*) Same image detail like (*E*), note the weaker calcitic crystallitic b-fabric (xpl).

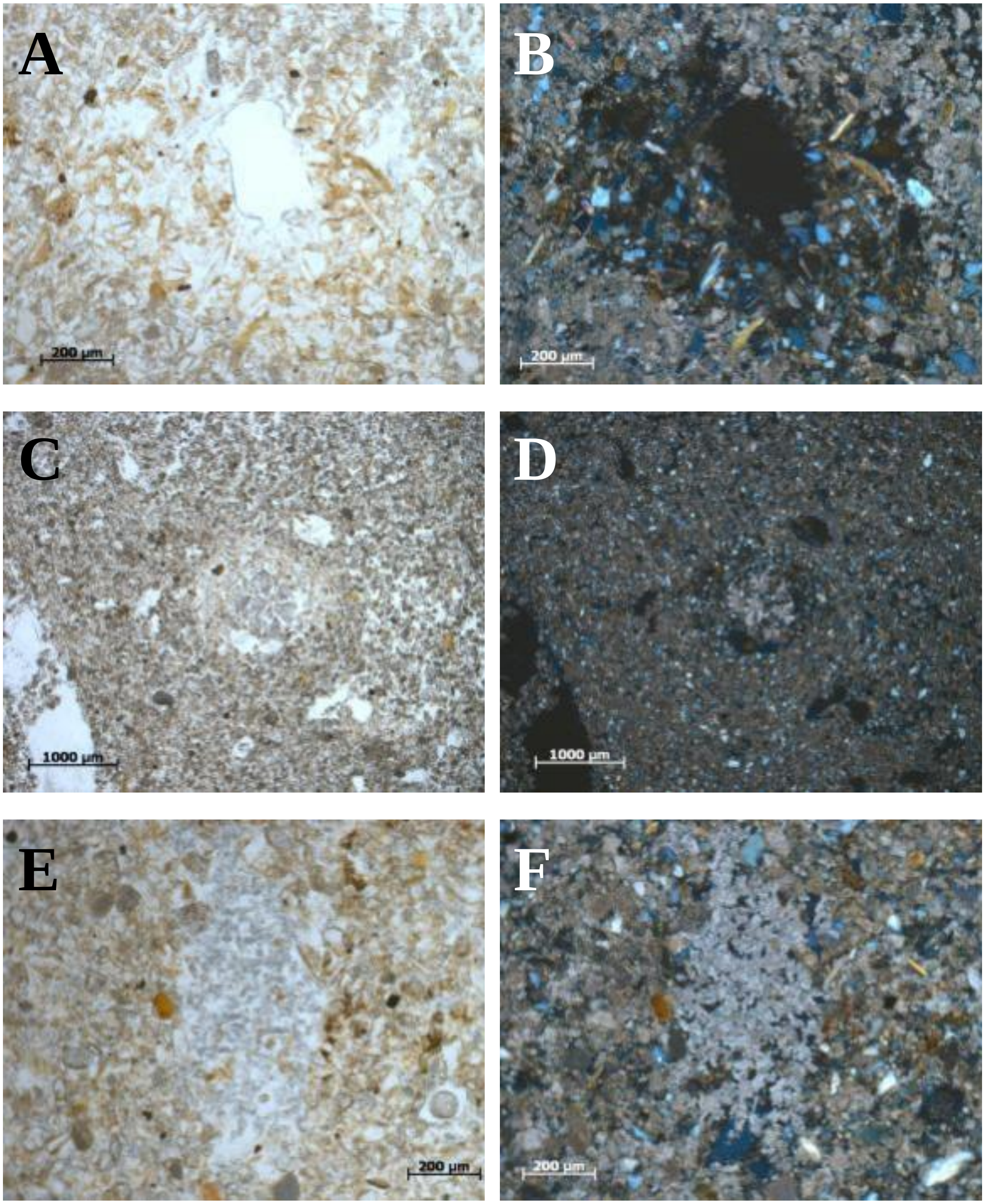

Fig. S14. (*A*) Micromorphology sample Pa1. Depletion hypocoating (DH) (ppl). (*B*) Same image detail like (*A*), note the speckled b-fabric of the matrix adjacent to the void (xpl). (*C*) Micromorphology sample Pa10. Compound depletion hypocoating and calcitic infilling (DH-CI) (ppl). (*D*) Same image detail like (*C*) (xpl). (*E*) Micromorphology sample Pa4. Calcitic crystals as infilling of a void (CC/CI) (ppl). (*F*) Same image detail like (*E*) (xpl).

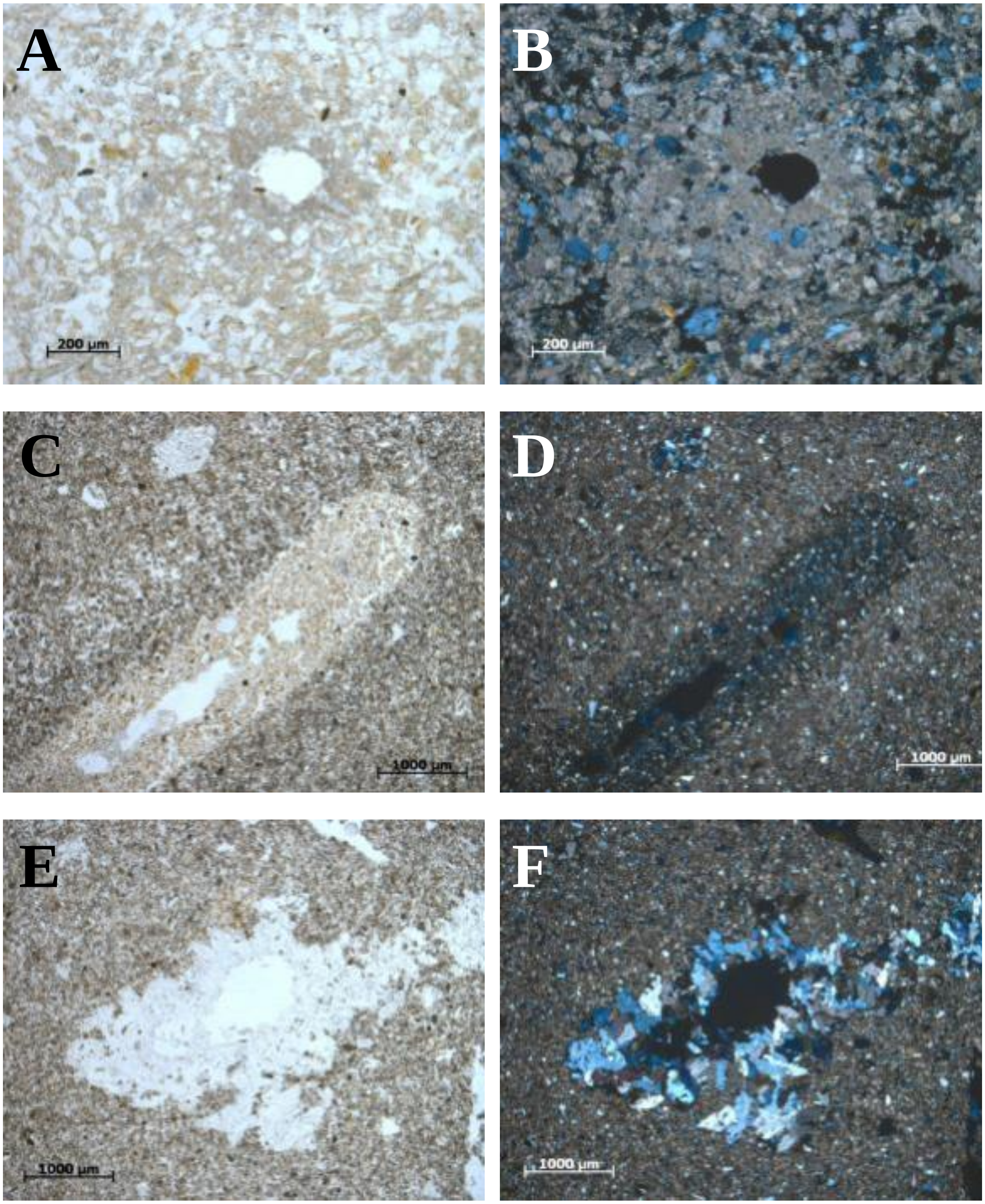

Fig. S15. (*A*) Micromorphology sample Pa3. Calcitic hypocoating (CH) (ppl). (*B*) Same image detail like (*A*), note the strong crystallitic b-fabric of the matrix adjacent to the void (xpl). (*C*) Micromorphology sample Pa5. Compound calcitic hypocoating and depletion infilling (CH-DI) (ppl). (*D*) Same image detail like (*C*), note the different b-fabric of the hypocoating and the infilling (xpl). (*E*) Micromorphology sample Pa5. Gypsum coating (GC) (ppl). (*F*) Same image detail like (*E*) (xpl).

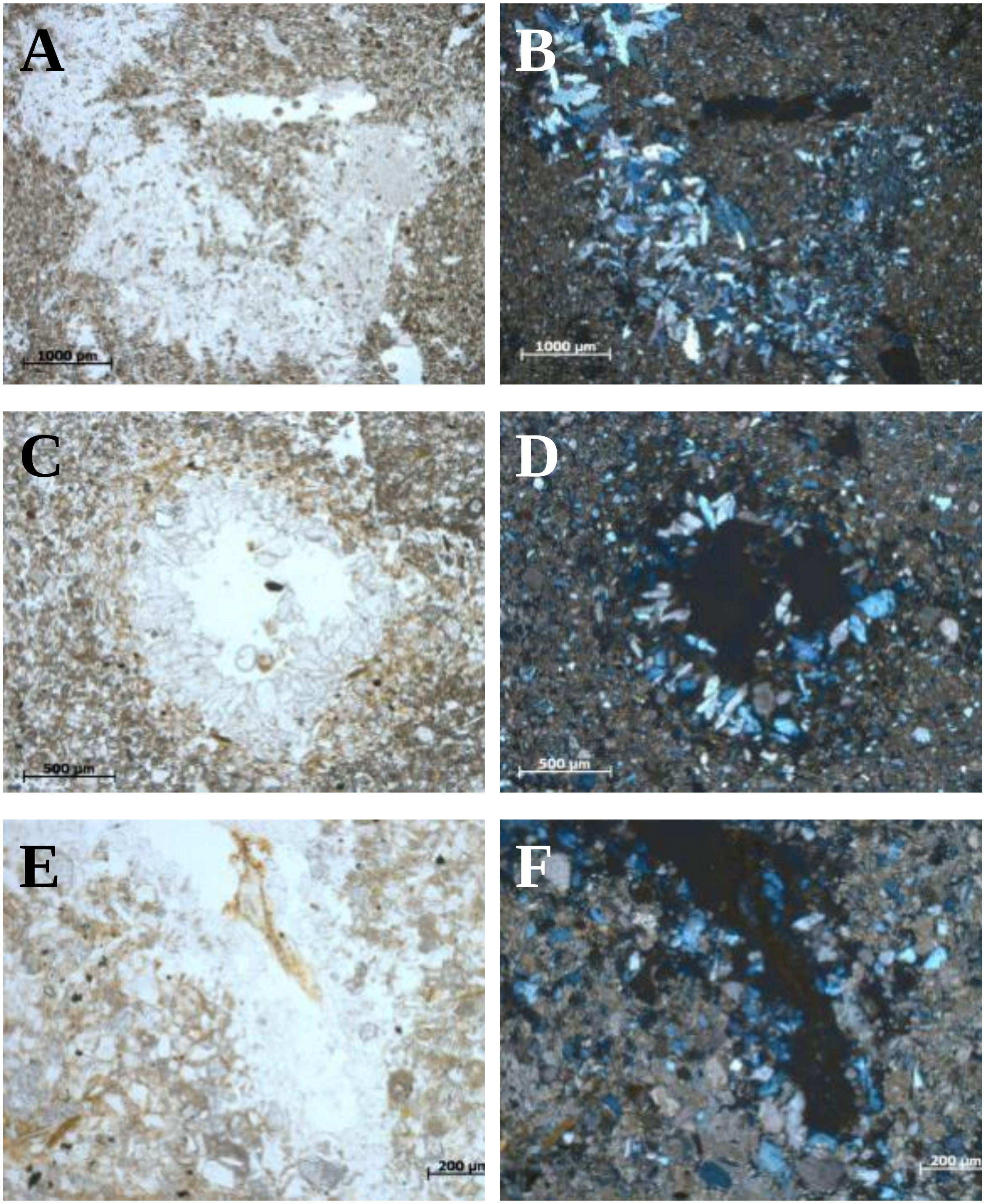

Fig. S16. (*A*) Micromorphology sample Pa4. Gypsum infilling (GI) (ppl). (*B*) Same image detail like (*A*), note the different shapes and sizes of the crystals (xpl). (*C*) Micromorphology sample Pa3. Compound depletion hypocoating and gypsum coating (DH-GC) (ppl). (*D*) Same image detail like (*C*) (xpl). (*E*) Micromorphology sample Pa3. Gypsum coating of a void (GC) with remains of a partly decomposed root in the center (ppl). (*F*) Same image detail like (*E*) (xpl).

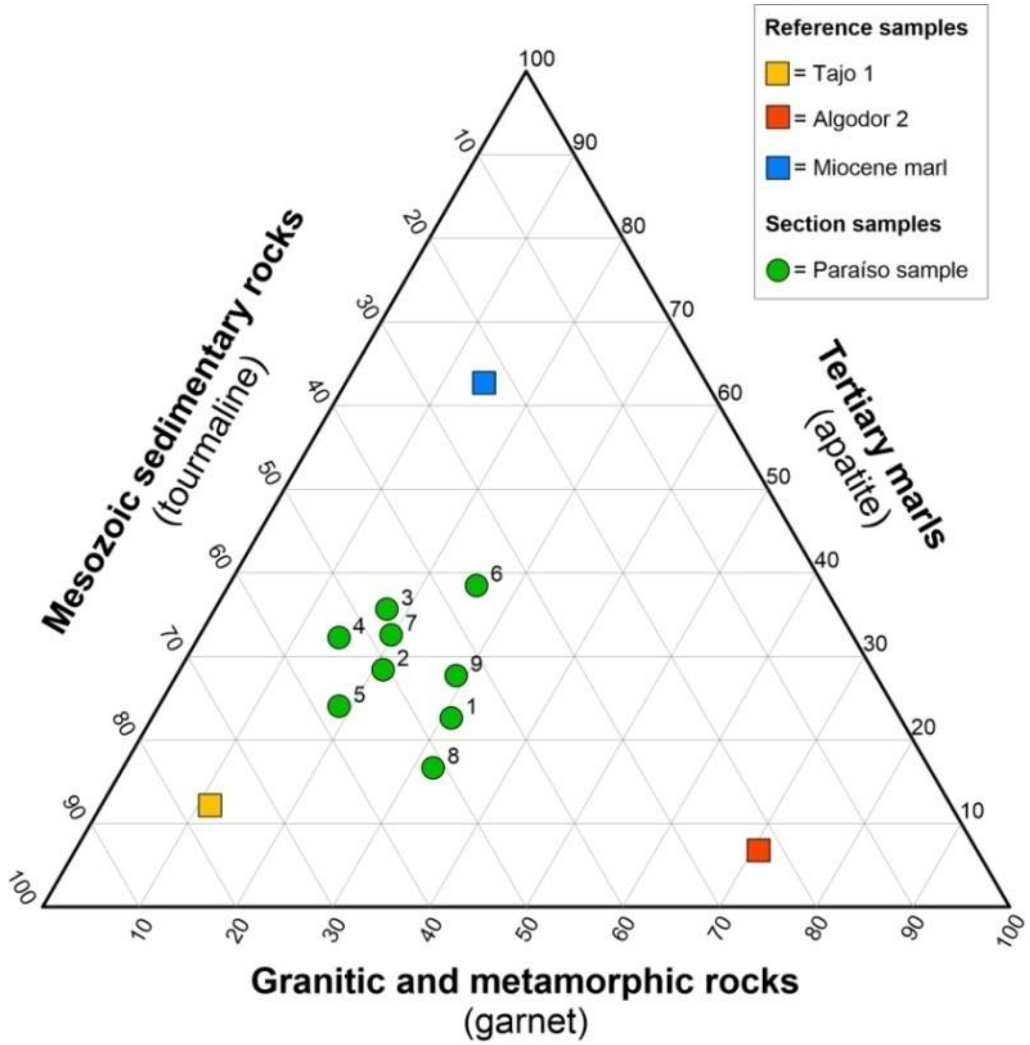

Fig. S17. Ternary plot displaying the composition of heavy mineral samples. Shown are nine loess samples from the Paraíso section, and three reference samples. Reference samples were taken from the Tagus floodplain that represents Mesozoic sedimentary rocks, the Algodor floodplain that represents granitic and metamorphic rocks, as well as Tertiary marls. Please note that this distribution is based on the relation between garnet, apatite, and tourmaline. Please reference Fig. S12 for sampling points.

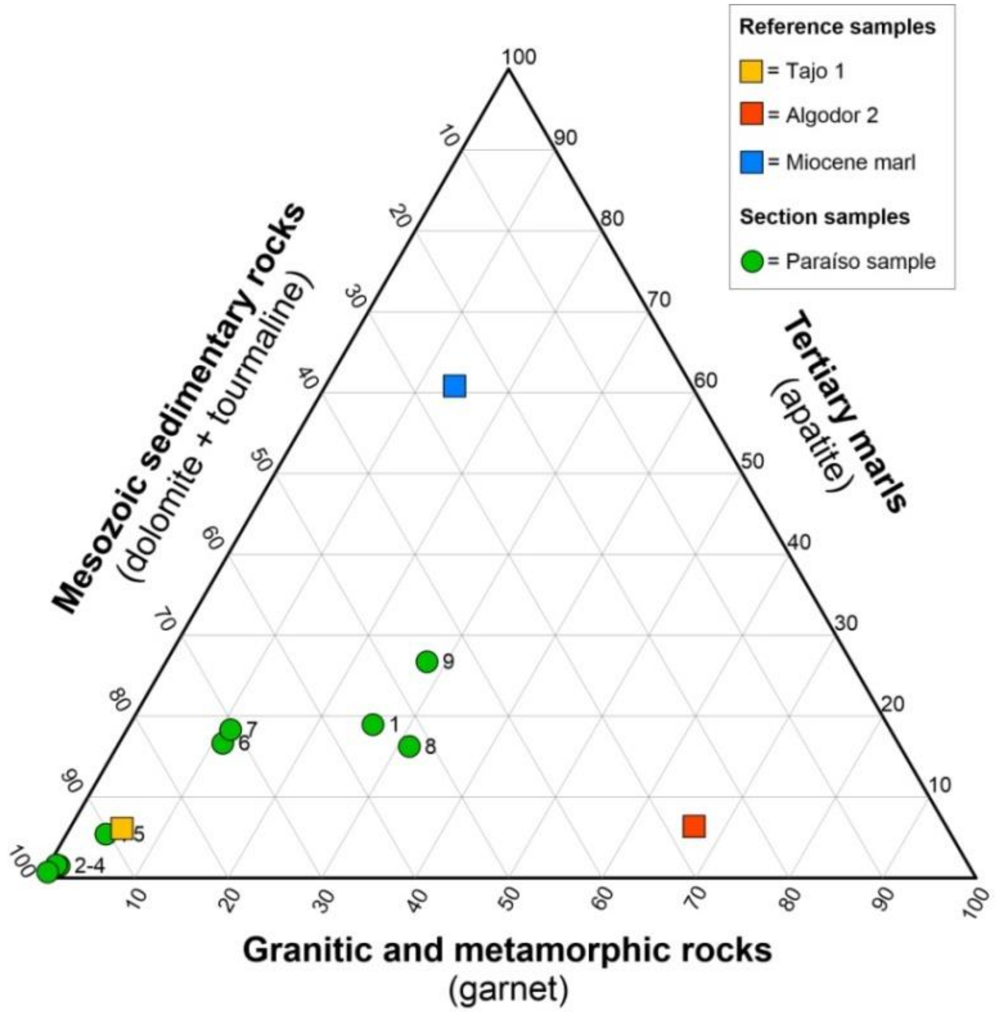

Fig. S18. Ternary plot displaying the composition of heavy mineral samples (including dolomite). Shown are nine loess samples from the Paraíso section, and three reference samples that were taken from the Tagus floodplain representing Mesozoic sedimentary rocks, the Algodor floodplain representing granitic and metamorphic rocks, as well as Tertiary marls. Please note that contrary to Fig. S17 this distribution is based on the relation between garnet, apatite, and dolomite + tourmaline. Please reference Fig. S12 for sampling points.

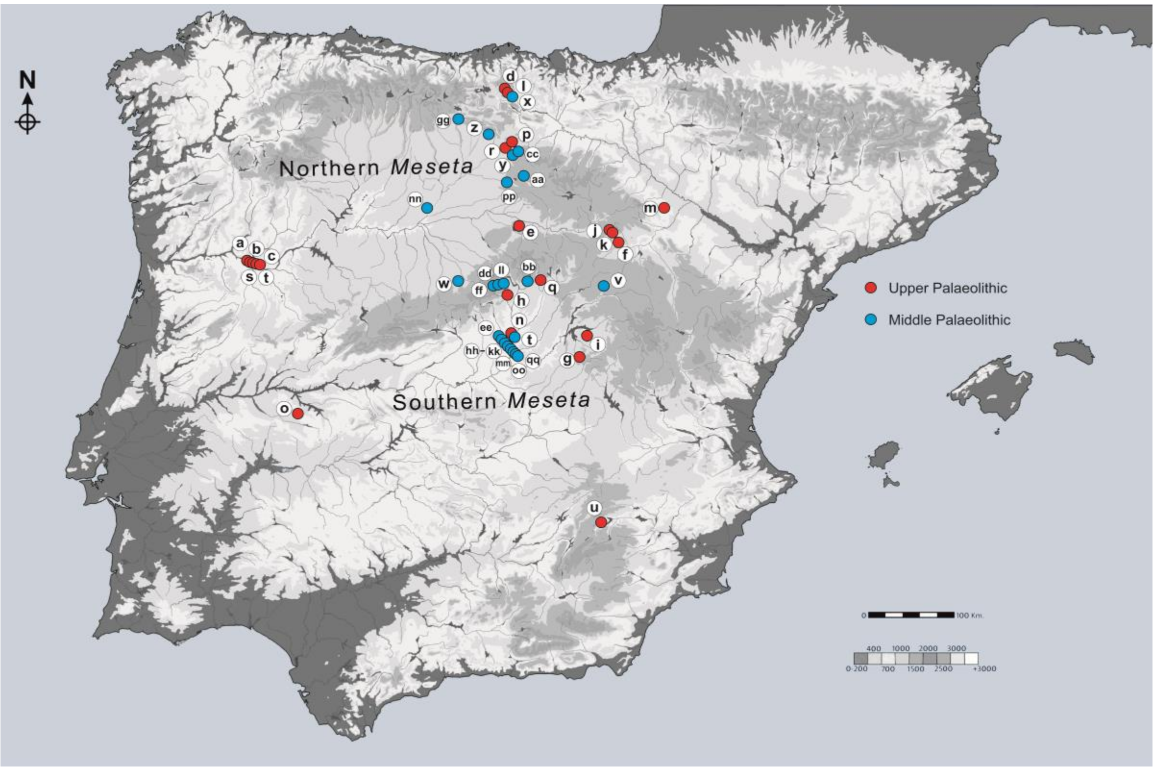

Upper Palaeolithic sites

(a):	Fariseu (open-air)	Duero Basin
(b):	Quinta da Barca Sul (open-air)	Duero Basin
(c):	Foz do Medal (open-air)	Duero Basin
(d):	Ojo Guareña - Gueva Palomera	Duero Basin
(e):	La Peña de Estebanvela rock shelter	Duero Basin
(f):	Peña del Diablo 1 rock shelter	Ebro Basin
(g):	Verdelpino rock shelter	Guadiana Basin
(h):	El Monte rock shelter	Tagus Basin
(i):	Buendía rock shelter	Tagus Basin
(j):	Vergara rock shelter	Ebro Basin
(k):	Alexandre rock shelter	Ebro Basin
(l):	Ojo Guareña - Galería de las Huellas	Duero Basin
(m):	Gato 2 rock shelter	Ebro Basin
(n):	Las Delicias (open-air)	Tagus Basin
(o):	Maltrevieso cave	Tagus Basin
(p):	Cueva Mayor cave	Duero Basin
(q):	Peña Capón rock shelter	Tagus Basin
(r):	Valle de Orquídeas (open-air)	Duero Basin
(s):	Cardina 1 (open-air)	Duero Basin
(t):	Olga Grande 4 (open-air)	Duero Basin
(u):	El Palomar rock shelter	Baetic Mountains

Middle Palaeolithic sites

(v):	Los Casares	Tagus Basin
(w):	El Molino rock shelter	Duero Basin
(x):	Prado Vargas cave	Duero Basin
(y):	Hotel California (open-air)	Duero Basin
(z):	Valdegoba cave	Duero Basin
(aa):	La Mina cave	Duero Basin
(bb):	Jarama VI rock shelter	Tagus Basin
(cc):	Hundidero (open-air)	Duero Basin
(dd):	Buena Pinta cave	Tagus Basin
(ee):	Estragales III (open-air)	Tagus Basin
(ff):	Navamaíllo (open-air)	Tagus Basin
(gg):	San Quirce (open-air)	Duero Basin
(hh):	Estanque de Tormentas (open-air)	Tagus Basin
(ii):	Preresa (open air)	Tagus Basin
(jj):	La Gavia III (open-air)	Tagus Basin
(kk):	Estragales II (open-air)	Tagus Basin
(ll):	Camino cave	Tagus Basin
(mm):	Arriaga (open air)	Tagus Basin
(nn):	Corazón cave	Duero Basin
(oo):	Estragales I (open air)	Tagus Basin
(pp):	La Ermita cave	Duero Basin
(qq):	Edar-Culebro I (open-air)	Tagus Basin

Fig. S19. Location of the archaeological sites in interior Iberia. Sites are referenced in SI Text 9 and Tables S7 and S8. Red dots refer to Upper Palaeolithic sites and blue dots refer to Middle Palaeolithic sites. The map was generated using ArcGIS 10.2.2 (http://www.esri.com/software/arcgis).

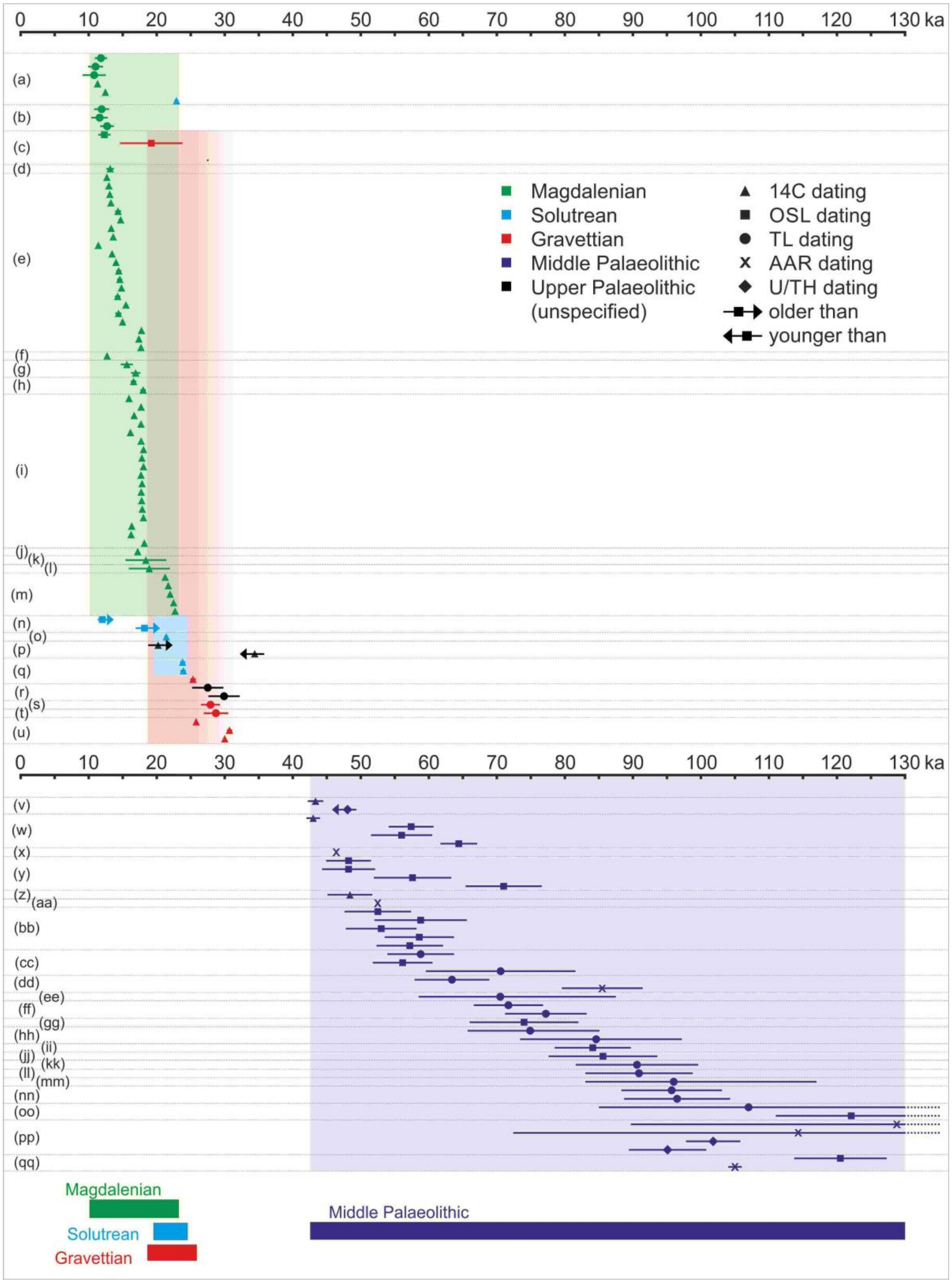

Fig. S20. Timeline of the Late Pleistocene human occupation of interior Iberia and deduced occupational stages. Numbering refers to Fig. S19.

Table S1. Analytic data for dose rate determination. Sample codes, water contents, radionuclide-concentrations, cosmic and total dose rates for all investigated quartz samples.

Laboratory code	Moisture [wt.%]	U [ppm][a]	Th [ppm][a]	K [wt.%][b]	$\dot{D}_{cosmic}$ [Gy/ka][c]	Total dose rate $\dot{D}$ [Gy/ka][d]
Fuentidueña section						
HUB 470	0.05 ± 0.03	4.16 ± 0.30	9.12 ± 0.97	1.17 ± 0.12	0.16 ± 0.02	2.75 ± 0.13
HUB 471	0.05 ± 0.03	4.42 ± 0.35	12.93 ± 1.16	1.29 ± 0.13	0.17 ± 0.02	3.18 ± 0.15
BT 1364	0.05 ± 0.03	4.48 ± 0.39	8.84 ± 1.27	1.40 ± 0.14	0.20 ± 0.02	3.06 ± 0.16
BT 1365	0.05 ± 0.03	3.81 ± 0.41	11.11 ± 1.37	1.34 ± 0.13	0.18 ± 0.02	2.99 ± 0.16
BT 1366	0.05 ± 0.03	5.44 ± 0.48	13.01 ± 1.48	1.34 ± 0.13	0.16 ± 0.02	3.44 ± 0.17
BT 1367	0.05 ± 0.03	3.88 ± 0.38	8.25 ± 1.25	1.07 ± 0.11	0.14 ± 0.01	2.53 ± 0.13
BT 1368	0.05 ± 0.03	3.97 ± 0.42	10.49 ± 1.39	0.97 ± 0.10	0.13 ± 0.01	2.58 ± 0.14
A3 section						
HUB 472	0.05 ± 0.03	3.48 ± 0.27	7.79 ± 0.87	1.11 ± 0.11	0.17 ± 0.02	2.48 ± 0.12
BT 1369	0.05 ± 0.03	3.63 ± 0.24	7.15 ± 0.80	1.08 ± 0.11	0.20 ± 0.02	2.47 ± 0.12
BT 1370	0.05 ± 0.03	3.38 ± 0.38	9.18 ± 1.27	1.18 ± 0.12	0.18 ± 0.02	2.62 ± 0.14
BT 1371	0.05 ± 0.03	3.87 ± 0.28	7.05 ± 0.91	1.15 ± 0.12	0.17 ± 0.02	2.56 ± 0.12
BT 1372	0.05 ± 0.03	3.17 ± 0.25	6.04 ± 0.82	1.23 ± 0.12	0.17 ± 0.02	2.41 ± 0.12
BT 1373	0.05 ± 0.03	3.68 ± 0.30	8.73 ± 1.00	1.13 ± 0.11	0.16 ± 0.02	2.59 ± 0.13
BT 1374	0.05 ± 0.03	3.88 ± 0.39	9.78 ± 1.30	1.34 ± 0.13	0.16 ± 0.02	2.90 ± 0.15
Paraíso section						
BT 1375	0.08 ± 0.03	4.18 ± 0.41	10.62 ± 1.37	1.38 ± 0.14	0.22 ± 0.02	3.02 ± 0.15
BT 1376	0.05 ± 0.03	5.05 ± 0.50	14.66 ± 1.66	1.53 ± 0.15	0.19 ± 0.02	3.67 ± 0.19
BT 1377	0.05 ± 0.03	4.06 ± 0.28	10.84 ± 0.93	1.36 ± 0.14	0.17 ± 0.02	3.04 ± 0.14
BT 1378	0.05 ± 0.03	3.89 ± 0.36	9.98 ± 1.18	1.29 ± 0.13	0.15 ± 0.02	2.86 ± 0.15
BT 1379	0.05 ± 0.03	5.04 ± 0.40	11.17 ± 1.32	1.50 ± 0.15	0.13 ± 0.01	3.36 ± 0.17
BT 1380	0.05 ± 0.03	5.39 ± 0.37	9.10 ± 1.22	1.40 ± 0.14	0.13 ± 0.01	3.21 ± 0.16
BT 1381	0.08 ± 0.03	4.51 ± 0.36	9.55 ± 1.20	1.48 ± 0.15	0.12 ± 0.01	3.01 ± 0.15
BT 1382	0.05 ± 0.03	4.58 ± 0.40	12.13 ± 1.33	1.50 ± 0.15	0.11 ± 0.01	3.31 ± 0.17
BT 1383	0.08 ± 0.03	2.81 ± 0.21	6.87 ± 0.70	1.06 ± 0.11	0.11 ± 0.01	2.09 ± 0.10
BT 1384	0.08 ± 0.03	3.34 ± 0.21	6.65 ± 0.71	1.08 ± 0.11	0.10 ± 0.01	2.28 ± 0.11
BT 1544	0.08 ± 0.03	3.93 ± 0.38	11.34 ± 1.26	1.43 ± 0.14	0.22 ± 0.02	3.16 ± 0.16

[a] Determined by thick source α-counting.
[b] Determined by ICP-OES.
[c] Cosmic dose rates calculated according to ref. 57.
[d] Environmental dose rates were calculated applying DRAC v1.2 (58) in combination with the conversion factors given by ref. 59.

Table S2. Analytical data for luminescence age calculation: Sample codes, used preheat and cutheat temperatures, number of aliquots, number of aliquots supposed to be in saturation, total dose rates, equivalent doses and OSL ages.

Laboratory code	Field code	PH | CH [°C] [a]	N[b]	n[c]	n_{sat}[d]	Total dose rate $\dot{D}$ [Gy/ka]	Equivalent Dose D_e [Gy] [e]	Luminescence Age [ka]
Fuentidueña section								
HUB 471	FU-07	240 | 180	24	19	-	3.18 ± 0.15	51.57 ± 3.67	16.20 ± 1.38
HUB 470	FU-03	240 | 180	24	15	5	2.75 ± 0.13	113.83 ± 9.51	41.34 ± 3.97
BT 1364	FU-06	240 | 220	15	14	-	3.06 ± 0.16	78.53 ± 4.24	25.62 ± 1.91
BT 1365	FU-05	240 | 220	15	14	-	2.99 ± 0.16	96.03 ± 6.33	32.15 ± 2.71
BT 1366	FU-04	240 | 220	25	24	1	3.44 ± 0.17	109.29 ± 7.22	31.73 ± 2.61
BT 1367	FU-02	240 | 220	24	19	5	2.53 ± 0.13	184.45 ± 14.53	73.03 ± 6.92
BT 1368	FU-01	240 | 220	24	9	14	2.58 ± 0.14	208.21 ± 17.79	80.67 ± 8.09
A3 section								
HUB 472	A3-03	240 | 180	24	23	-	2.48 ± 0.12	69.76 ± 4.95	28.35 ± 2.44
BT 1369	A3-07	180 | 160	25	23	-	2.47 ± 0.12	57.18 ± 3.03	23.16 ± 1.64
BT 1370	A3-06	180 | 160	25	21	-	2.62 ± 0.14	61.09 ± 3.23	23.29 ± 1.75
BT 1371	A3-05	200 | 180	24	19	-	2.56 ± 0.12	64.57 ± 4.00	25.27 ± 2.00
BT 1373	A3-04	200 | 180	22	18	-	2.59 ± 0.13	63.92 ± 4.08	24.67 ± 1.99
BT 1372	A3-02	220 | 200	27	16	10	2.41 ± 0.12	271.69 ± 25.09	112.59 ± 11.92
BT 1374	A3-01	220 | 200	32	20	7	2.90 ± 0.15	276.25 ± 22.34	95.38 ± 9.21
Paraíso section								
BT 1544	PA-11	180 | 160	12	11	-	3.16 ± 0.16	0.97 ± 0.25	0.31 ± 0.08
BT 1375	PA-10	180 | 160	25	14	-	3.02 ± 0.15	0.88 ± 0.09	0.29 ± 0.03
BT 1376	PA-09	240 | 220	29	27	-	3.67 ± 0.19	95.11 ± 7.21	25.89 ± 2.36
BT 1377	PA-08	240 | 220	25	22	-	3.04 ± 0.14	94.82 ± 5.77	31.22 ± 2.40
BT 1378	PA-07	200 | 180	24	21	-	2.86 ± 0.15	87.12 ± 5.35	30.49 ± 2.43
BT 1379	PA-06	220 | 200	24	24	-	3.36 ± 0.17	144.55 ± 10.47	42.96 ± 3.78
BT 1380	PA-05	220 | 200	24	24	-	3.21 ± 0.16	136.38 ± 9.24	42.53 ± 3.56
BT 1381	PA-04	240 | 220	32	26	3	3.01 ± 0.15	179.63 ± 10.84	59.69 ± 4.71
BT 1382	PA-03	220 | 200	25	21	4	3.31 ± 0.17	211.90 ± 16.76	64.11 ± 6.03
BT 1383	PA-02	200 | 180	24	11	12	2.09 ± 0.10	222.59 ± 18.56	106.31 ± 10.32
BT 1384	PA-01	200 | 180	24	11	12	2.28 ± 0.11	213.02 ± 14.07	96.53 ± 7.90

[a] Preheat (PH) and cutheat (CH) temperature combinations used for the measurement procedures applied for equivalent dose determination.
[b] N = number of measured aliquots.
[c] n = number of aliquots passing the rejection criteria and used for equivalent dose determination. For t details on the rejection criteria the reader is kindly referred to the main text.
[d] n_{sat} = number of aliquots exceeding the $2D_0$-criterion and therefore supposed to be in saturation. Please note that samples showing a high number of saturated aliquots have to be taken with care since the calculated ages can only be interpreted as minimum ages.
[e] Equivalent doses were calculated as unweighted means. No specific age model was applied.

Table S3. Details on the used single aliquot regenerative (SAR) dose protocol (85, 86).

Step	Treatment	Result
1	Dose D_x[a]	-
2	Preheat (hold for 10s)[b]	-
3	OSL at 125°C for 40s	L_x
4	Test dose D_t	-
5	Cutheat (no holding)[c]	-
6	OSL at 125°C for 40s	T_x
7	OSL at 280°C for 40s (hot bleach step)	-

[a] For determining the natural signal no additional dose was given (i.e., x = n D_n= = 0 Gy).
[b] Preheat temperatures were individually chosen for each sample, derived from the results of a combined dose recovery and preheat test. For details, the reader is referred to the main text.
[c] Cutheat temperatures were always 20°C below the respective preheat temperatures. This does not apply to samples HUB 470, HUB 471 and HUB 472 (preheat temperature = 240°C; cutheat temperature = 180°C).

Table S4. Radiocarbon dating on *n*-alkanes. Sample labels refer to sampling points of the Paraíso section shown in Fig. S12.

Sample label	Sample code	Carbon mass (μg)	$F^{14}C$	u	$\delta^{13}C$ (‰)	Age range (2σ) [cal. ka BP]
Pa 54	4800.1.1	49.9	0.5533	0.0077	-27.0	5.07-5.74
Pa 52	4801.1.1	77.2	0.5722	0.0078	-26.5	4.85-5.46
Pa 50	4802.1.1	45.3	0.2029	0.0066	-36.3	14.23-16.04
Pa 46	4803.1.1	57.2	0.1753	0.0056	-31.2	16.24-17.67
Pa 39	4804.1.1	79.2	0.0537	0.0037	-24.9	26.6-28.79

Table S5. Micromorphological features of the Paraíso section. See Fig. S12 for location of samples.

sample	MD	DH	DH-CI	CC/CI	CH	CH-DI	GC/GI	DH-GC	Bio
Pa10	-	(•)	•	•	•	(•)	-	-	••
Pa9	-	(•)	-	-	-	(•)	-	-	••
Pa8	-	(•)	-	•	-	(•)	-	-	••
Pa7	-	•	-	-	•	••	-	-	•••
Pa6	-	-	-	•	-	••	••	••	•••
Pa5	-	-	-	-	-	•••	•••	-	•••
Pa4	(•)	••	-	••	-	-	•••	-	••
Pa3	-	••	-	-	••	-	•••	•	••
Pa2	-	-	-	•	•••	-	••	-	•
Pa1	•	••	(•)	•	-	-	••	-	••

		**MD**	matrix depletion
		**DH**	depletion hypocoating
		**DH-CC**	compound depletion hypocoating and calcitic infilling
		**CC/CI**	calcitic coating/ calcitic infilling
-	absent	**CH**	calcitic hypocoating
(•)	very rare/very weak	**CH-DI**	compound calcitic hypocoating and depletion infilling
•	rare/weak	**GC/GI**	gypsum coating/gypsum infilling
••	few/average	**DH-GC**	compound depletion hypocoating and gypsum coating
•••	common/strong	**Bio**	Bioturbation

Table S6. Results of heavy mineral analyses. Shown are samples from the section Paraíso together with three reference samples. See Fig. S12 for the location of samples and SI Text 8 for discussion of the results**.**

	Rf	Op	Mca	Dol	Ep	Ol	Zrn	Ttn	Grt	S,A,D	Toz	St	Tur	Px	Hbl	G,R	Tk	Cst	Rt	Ant	Brk	Ap
Loess samples																						
Para_1	403	92	36	16	24	1	49	2	26	10	23	7	39	2	-	1	-	4	2	-	3	19
Para_2	509	106	2291	1420	24	1	25	9	17	13	25	18	41	2	8	-	6	3	1	5	1	23
Para_3	484	64	2616	1527	21	-	34	3	13	4	22	10	34	2	7	-	-	7	-	3	-	26
Para_4	653	81	2087	2577	25	5	41	1	9	6	10	5	33	4	7	-	-	6	1	-	-	20
Para_5	546	95	577	258	23	3	39	-	14	11	10	8	43	-	8	-	-	2	2	1	1	18
Para_6	481	91	476	102	24	-	28	2	20	10	16	2	28	-	5	-	-	5	1	-	1	30
Para_7	421	77	812	67	37	2	37	-	17	13	9	5	41	1	4	2	1	4	-	-	-	28
Para_8	370	105	23	2	32	3	44	5	25	7	8	7	40	2	4	-	-	7	2	-	1	13
Para_9	404	68	54	3	39	-	25	3	24	9	3	9	36	4	3	-	-	-	2	-	-	23
Reference samples																						
Marl	364	71	103	4	23	-	21	4	18	4	11	2	29	2	3	-	-	2	-	-	1	79
Tajo 1	914	219	25	107	30	-	77	-	12	7	56	8	82	-	9	-	-	2	8	-	-	13
Algodor 2	328	181	31	8	31	-	40	-	94	7	69	5	30	-	7	-	-	-	-	-	-	9

Rf = rock fragments; Op = opaque minerals; Mca = mica; Dol = dolomite; Ep = Epidote; Ol = olivine group; Zrn = zircon; Ttn = titanite; Grt = garnet; S,A,D = sillimanite, andalusite, disthene; Toz = topaz; St = staurolithe; Tur = tourmaline; Px = pyroxene; Hbl = hornblende; G,R = glaucophane, riebeckite; Tk = talc; Cst = cassiterite; Rt = rutile; Ant = anatase; Brk = brookite; Ap = apatite

Table S7. Middle Palaeolithic sites having yielded chronometric dates in interior Iberia. Unless otherwise indicated, all dated layers contain archaeological (Mousterian) or palaeoanthropological (Neanderthal) evidence. Results are presented as calibrated ages BP at the 95.4% probability using OxCal 4.3 (60) and IntCal13 (61) in case of radiocarbon dating. Dates of sites marked with * are not considered reliable (but see SI Text 9 for discussion).

Site	Location	Dates	References	Notes
*** Cañaveral-Área 3 (open-air)**	Madrid Basin	33.0 + 4.0/ - 3.5 ka (TL) - Level 2<br>96.0 + 34.0/ - 21.0 ka (TL) - Level 3	*(31)*	Unreliable dates considering published data.
*** 12 de Octubre (open-air)**	Madrid Basin	c. 33-40 ka (OSL) (see Silva *et al.*[62]: Table 2)	*(62)*	Unreliable dates due to high saturation of samples.
*** Millán rock shelter**	Eastern Duero Basin. Burgos	43.0 – 40.9 cal BP (Conventional 14C on charcoal) - Level 1a<br>42.8 – 40.8 cal BP (Conventional 14C on charcoal) - Level 1b	*(35)*	Unreliable dates due to conventional 14C method.
**Los Casares**	Upper Tagus Basin - Iberian Range. Guadalajara	42.2 – 44.5 (AMS on charcoal) - Level c<br>48.1 ± 0.2 (U/Th on flowstone) – Level d	*(31)*	Dating of level d is a *terminus post quem* for human occupation.
**El Molino rock shelter**	Middle Duero Basin-Central System northern foothills. Segovia	c. 42 - 44 ka cal BP (AMS on bone) - Level 2<br>57.4 ± 3.3 ka (OSL) - Level 3G<br>64.4 ± 2.7 ka (OSL) - Level 3E<br>56 ± 4.5 ka (OSL) - Unit M (sterile)	*(29, 30)*	See ref. 29 for other dates considered too young. Complete radiocarbon dates still to be published.
**Prado Vargas cave**	Eastern Duero Basin-Southern foothills of the Cantabrian Range. Burgos	46.4 ka (AAR on horse tooth) - Level 4	*(63)*	
**Hotel California (open-air)**	Eastern Duero Basin-Atapuerca Mountains. Burgos	48.2 ± 3.3 ka (OSL) - Unit V (top level)<br>48.2 ± 3.9 ka (OSL) - Unit V (top level)<br>57.6 ± 5.7 ka (OSL) - Unit II<br>71.0 ± 5.6 ka (OSL) - Unit I (base level)	*(33, 36)*	

**Valdegoba cave**	Eastern Duero Basin-Southern foothills of the Cantabrian Range. Burgos	48.4 ± 3.3 ka BP (AMS, ultrafiltration) - Level V (Unit D)<br>*c*. 75 and 93 ka (U/Th) -Capping speleothem.<br>*c*. 72-138 ka (AAR) - Levels IV-VI (Unit D)	*(34)*	Detritial contamination suggest U/Th dates are too old.<br>AAR results contradict both radiocarbon and U/Th data.
**La Mina cave**	Eastern Duero Basin. Burgos	52.5 ka (AAR on tooth) - Reworked deposit	*(35)*	
**Jarama VI rock shelter**	Upper Tagus Basin-Southeastern foothills of the Central System. Guadalajara	>50.2 14C ka BP on cut-marked bone - Level JVI.1<br>49.4 ± 3.7 14C ka BP on cut-marked bone - Level JVI.2.2<br>>47 14C ka BP on cut-marked bone - Level JVI.3<br>c. 50-60 ka (post-IR IRSL and AMS) - Levels JVI.2.2 and JVI.2.3.	*(26, 28)*	See ref. 28 for a list of luminescence dates
**Hundidero (open-air)**	Eastern Duero Basin-Atapuerca Mountains. Burgos	58,788 ± 4907 (TL) - Base of Level H2<br>56,157 ± 4389 (OSL) - Level H3<br>70,556 ± 11,011 (TL) - Top of Level H4	*(36, 64)*	A date of 30,221 ± 3636 BP (OSL - Top of Level H2) was dismissed by the site's workers due to chronostratigraphic inconsistency.
**Buena Pinta cave**	Southern foothills of the Central System. Madrid	63.4 ± 5.5 ka (TL) - Level 3<br>85.5 ± 11.9 ka (AAR) - Level 3	*(65)*	
**Estragales III (open-air)**	Madrid basin	70. 5 + 17/- 12 ka (TL)	*(66)*	
**Navalmaíllo (open-air)**	Southern foothills of the Central System. Madrid	71.7 ± 5.1 ka (TL) - Level F<br>77.2 ± 6.0 ka (TL) - Level F	*(67)*	
**San Quirce (open-air)**	Eastern Duero Basin-Southern foothills of the Cantabrian Range. Palencia	*c*.73-74 ka (Median of OSL dates).	*(68)*	Ages are considered only a "rough estimate" due to saturated or nearly saturated signals.
**Estanque de Tormetas de Butarque (open-air)**	Madrid Basin	74.9 +10.2/−9.2 ka (TL) - Level H - 02<br>84.6 +12.6/−11.2 ka (TL) - Level H - 02	*(69)*	Dates contradict biocronology suggesting a Middle Pleistocene age (ref. 70).
**Preresa (open-air)**	Madrid Basin	84.1 ± 5.6 ka (OSL)	*(71)*	

**La Gavia III (open-air)**	Madrid Basin	85.6 ± 8 ka (OSL) - Level III	*(37)*	Level contains few lithics in a reworked deposit.
**Estragales II (open-air)**	Madrid Basin	90.6 ± 9 ka (TL)	*(66)*	
**Camino cave**	Southern foothills of the Central System. Madrid	90.9 ± 7.9 ka (TL) - Level 5	*(72)*	TL dates in other areas of the site not related to human occupation.
**Arriaga (open-air)**	Madrid Basin	96 + 21/ - 13 ka (TL) - Top of MZ5 Unit	*(73)*	
**Corazón cave**	Eastern Duero Basin-Southern foothills of the Cantabrian Range. Palencia	95.7 ± 7.4 ka (TL on thermoaltered quartzite) - Level 2<br>96.5 ± 7.8 ka (TL on thermoaltered quartzite) - Level 2	*(74)*	
**Estragales I (open-air)**	Madrid Basin	107 + 39/-22 ka (TL) Upper levels<br>122.1 ± 11.1 ka (OSL) - Upper levels	*(66)*	
**La Ermita cave**	Eastern Duero Basin. Burgos	128.8 ± 39.1 ka (AAR on teeth) - Level 5a<br>114.3 ± 41.9 ka (AAR on teeth) - Level 5b<br>101.8 ± 4.0 ka (U/TH on flowstone) - Top of the sequence.<br>95.1 ± 5.7 ka (U/TH on flowstone) - Top of the sequence.	*(24, 35)*	A date of 36.6 – 34.7 cal BP (14C on charcoal- Level 5a) is unreliable due to low collagen and conventional 14C method.
**Edar-Culebro I (open-air)**	Madrid Basin	120.5 ± 6.8 ka (OSL) - Unit 2<br>133 ± 2.8 and 105 ± 1 ka (AAR on horse molars) - Unit 2	*(37)*	

Table S8. Upper Palaeolithic sites having yielded chronometric dates in interior Iberia. Unless otherwise indicated, all dated layers contain archaeological or palaeoanthropological (modern humans) evidence. Results are presented as calibrated ages BP at the 95.4% probability using OxCal 4.3 (60) and IntCal13 (61).

Site	Location	Dates & cultural attribution	References	Notes
**Fariseu (open-air)**	Western Duero Basin. Trás-os-Montes e Alto Douro	10.8-11.8 ka (TL/OSL) - Level 4 (Final Magdalenian)<br>11.1–11.5 ka cal BP (AMS on charcoal) - Level 4 (Final Magdalenian)<br>12.3–12.6 ka cal BP (AMS on charcoal) - Level 4 (Final Magdalenian)<br>22.6–23.2 ka cal BP (AMS on charcoal) - Level 9 (Upper Solutrean)	*(41, 75)*	See ref. 41 for a complete list of luminescence dates.
**Quinta da Barca Sul (open-air)**	Western Duero Basin. Trás-os-Montes e Alto Douro	Average of 12.1 ± 0.6 ka (TL on burnt pebble) - Magdalenian	*(40, 41)*	See ref. 40 for a complete list of dates.
**Foz do Medal Right Bank (open-air)**	Western Duero Basin. Trás-os-Montes	12.3 ± 0.9 ka (OSL) - Slope deposit covering level 1055 (Magdalenian)<br>19.2 ± 4.6 ka (OSL) - Level 1098 (Gravettian)<br>* 25.1 + 2.6/-2.3 Ka (TL on fire structure) - Level 1184 (EUP) (Structure 1332)<br>* 31.5 – 31.1. Ka (AMS on charcoal from fire structure) - Level 1184 (EUP) (Structure 1332)	*(42)*	* Unreliable dates due to contradictory results and unpublished lithic assemblage.
**Ojo Guareña cave - Gueva Palomera**	Eastern Duero Basin-Southern foothills of the Cantabrian Mountains. Burgos.	*c*. 12.6 - 13.7 ka cal BP (black pigments of rock paintings).	*(76)*	
**La Peña de Estebanvela rock shelter**	Middle Duero Basin-Central System northern foothills. Segovia	c. 12.5 - 17.1 ka cal BP (21 AMS dates on charred material and sediment) – Levels I-VI (Middle, Upper and Late Magdalenian)	*(77)*	See ref. 77 for a complete list of dates.

**Peña del Diablo 1 rock shelter**	Middle Ebro Basin. Zaragoza	12.7 ± 0.2 ka cal BP (Conventional 14C on charcoal) - Level 2 (Final Magdalenian)	*(78)*	
**Verdelpino rock shelter**	Eastern Guadiana Basin. Cuenca	15.6 ± 0.9 ka cal BP (Conventional 14C) - Level VA (Magdalenian)<br>16.9 ± 0.7 ka cal BP (Conventional 14C) - Level VB (Magdalenian)	*(79)*	
**El Monte rock shelter**	Upper Tagus Basin-Southeastern foothills of the Central System. Madrid	16.6 ± 0.4 ka cal BP (AMS on bone)- Level I (Magdalenian)<br>18.0 ± 0.4 ka BP (AMS on bone)- Level II (Magdalenian)	*(80)*	
**Buendía rock shelter**	Upper Tagus Basin. Cuenca	15.7 – 18.4 ka cal BP – Levels N1W-N33C (18 AMS dates on charcoal and bone)	*(81)*	See ref. 81 for a complete list of dates.
**Vergara rock shelter**	Middle Ebro Basin. Soria	17.2 ± 0.2 ka cal BP (AMS on horse tooth)- Level D (Lower Magdalenian)	*(78)*	
**Alexandre rock shelter**	Middle Ebro Basin. Soria	18.4 ± 3 ka cal BP (Conventional 14C on charcoal) - Level IIIb (Lower Magdalenian)	*(78)*	
**Ojo Guareña cave - Galería de las Huellas**	Eastern Duero Basin - Southern foothills of the Cantabrian Mountains. Burgos	18.9 ± 3 ka cal BP (charcoal on torch) - Rock art context	*(76)*	The date was obtained in 1974.
**Gato 2 rock shelter**	Middle Ebro Basin. Zaragoza	*c*. 22.8 - 21.0 ka cal BP (4 AMS dates on bone and charcoal) - Level 2 (Archaic Magdalenian)	*(78)*	See ref. 78 for a complete list of dates.
**Puente de los Tres Ojos (open-air)**	Madrid Basin	11.7 ± 8 ka (OSL) - Level 3a<br>14.4± 0.7 ka (OSL) – Level 1c (Solutrean in reworked deposits 2a and 2b between dated levels)	*(82)*	
**Las Delicias (open-air)**	Madrid Basin	12.0 ± 0.7 ka (OSL) - Sector II-Level 3b (Solutrean in lower level 4)<br>18.2 ± 1.3 ka (OSL) - Sector I-Level IIb (Solutrean; and also in lower levels IIc and IId)	*(49)*	Dates are considered terminus *terminus ante quem* for human occupation.

**Maltravieso cave**	Lower Tagus Basin. Cáceres	21.4 ± 0.3 (AMS on charcoal) - Sala de las Chimeneas Level A (Solutrean?)	*(83)*	
**Cueva Mayor cave**	Eastern Duero Basin - Atapuerca Mountains. Burgos	20.2 ± 0.3 ka cal BP (AMS on bone) - Level 10 P-1<br>34.4 ± 0.2 ka cal BP (AMS on bone) - Level 10 P-11 (scarce Upper Palaeolithic in Level 10 P-8)	*(44)*	Scarce evidence of human occupation is found between the the two dated layers (level 10 P-8).
**Peña Capón rock shelter**	Upper Tagus Basin – Southeastern foothills of the Central System. Guadalajara	24.285 - 23,671 cal BP (AMS on tooth) - Level 2-1970 (Solutrean).<br>24.339 - 23735 cal BP (AMS on tooth) - Level 3-1970 (Proto-Solutrean).<br>25,820 - 25,265 cal BP (AMS on tooth) - Level 4-1970 (Gravettian).	*(48)*	
**Valle de las Orquídeas (open-air)**	Eastern Duero Basin - Atapuerca Mountains. Burgos	27.5 ± 2.3 ka (TL) - Terra rossa level (Upper Palaeolithic)<br>29.9 ± 2.3 ka (TL) - Terra rossa level (Upper Palaeolithic)	*(43)*	
**Cardina 1 (open-air)**	Western Duero Basin. Trás-os-Montes e Alto Douro	Average of 27.9 ± 1.4 ka (TL on burnt pebble) - Level 4.10 (Gravettian)	*(40, 41)*	See Table 1 in ref. 40 for a complete list of dates.
**Olga Grande 4 (open-air)**	Western Duero Basin. Trás-os-Montes e Alto Douro	Average of 28.7 ± 1.8 ka (TL on burnt pebble) - Level 3 (Gravettian)	*(40, 41)*	See Table 1 in ref. 40 for a complete list of dates.
**El Palomar rock shelter**	Southern foothills of the Baetic Mountains. Albacete	26.0 - 25.6 ka cal BP (AMS on bone) - Level 3 (Gravettian)<br>31.1. - 30. 3 ka cal BP (AMS on bone) - Level 4 (Gravettian)<br>30.0 - 29.9 ka cal BP (AMS on bone) - Level 5 (Gravettian)	*(45)*	Although still an interior site, it is located fully outside the Southern Meseta.

Supplementary References

1. Wallinga, J., Murray, A. & Wintle, A. The single-aliquot regenerative-dose (SAR) protocol applied to coarse-grain feldspar. *Radiation Measurement* **32**, 529-533 (2000).
2. Dietze, M. *et al.* The abanico plot: visualising chronometric data with individual standard errors. *Quaternary Geochronology* **31**, 12-18 (2016).
3. Li, B., Jacobs, Z., Roberts, R. G. & Li, S.-H. Review and assessment of the potential of post-IR IRSL dating methods to circumvent the problem of anomalous fading in feldspar luminescence. *Geochronometria* **41**, 178-201 (2014).
4. Buylaert, J.-P. *et al.* Optical dating of Chinese loess using sand-sized quartz: Establishing a time frame for Late Pleistocene climate changes in the western part of the Chinese Loess Plateau. *Quaternary Geochronology* **3**, 99-113 (2008).
5. Thiel, C., Buylaert, J.-P., Murray, A. S. & Tsukamoto, S. On the applicability of post-IR IRSL dating to Japanese loess. *Geochronometria* **38**, 369-378 (2011).
6. Chikaraishi, Y. & Naraoka, H. Compound-specific δD–δ13C analyses of n-alkanes extracted from terrestrial and aquatic plants. *Phytochemistry* **63**, 361-371 (2003).
7. Häggi, C., Zech, R., McIntyre, C., Zech, M. & Eglinton, T. I. On the stratigraphic integrity of leaf-wax biomarkers in loess paleosols. *Biogeosciences* **11**, 2455-2463 (2014).
8. Boixadera, J., Poch, R. M., Lowick, S. E. & Balasch, J. C. Loess and soils in the eastern Ebro Basin. *Quaternary International* **376**, 114-133 (2015).
9. Durand, N., Monger, C. H. & Canti, M. G. in *Interpretation of micromorphological features of soils and regoliths* (eds Stoops, G, Marcelino, V. & Mees, F.) 149-194 (Elsevier, Amsterdam, Boston, Heidelberg, London, New York, Oxford, Paris, San Diego, San Francisco, Singapore, Sydney, Tokyo, 2010).
10. Becze-Deák, J., Langohr, R. & Verecchia, E. P. Small scale secondary $CaCO_3$ accumulations in selected sections of the European loess belt. Morphological forms and potential for paleoenvironmental reconstruction. *Geoderma* **76**, 221-252 (1997).
11. Barta, G. Secondary carbonates in loess-paleosoil sequences: a general review. *Cent. Eur. J. Geosci.* **3**, 129-146 (2011).
12. Kemp, R. A. Distribution and genesis of calcitic pedofeatures within a rapidly aggrading loess-paleosol sequence in China. *Geoderma* **65**, 303-316 (1995).
13. Wieder, M. & Yaalon, D. H. Micromorphological fabrics and development stages of carbonate nodular forms related to soil characteristics. *Geoderma* **28**, 203-220 (1982).
14. Poch, R. M., Artieda, O., Herrero, J. & Lebedeva-Verba, M. in *Interpretation of micromorphological features of soils and regoliths* (eds Stoops, G, Marcelino, V. & Mees, F.) 149-194 (Elsevier, Amsterdam, Boston, Heidelberg, London, New York, Oxford, Paris, San Diego, San Francisco, Singapore, Sydney, Tokyo, 2010).

15. Dultz, S. & Kühn, P. Occurrence, formation, and micromorphology of gypsum in soils from the Central-German Chernozem region. *Geoderma* **129**, 230-250 (2005).

16. Balasch, J. C., Poch, R. M., Ruiz-Bellet, J. C. & Boixadera, J. Particle size and micromorphology of loess deposits in the Lower Ebro river valley. *Proceedings of the 14th International Working Meeting on Soil Micromorphology. Lleida 8-14 July 2012* (2012).

17. Mange, M. A. & Maurer, H. F. *Schwerminerale in Farbe* (Enke, Stuttgart, 1991).

18. Boenigk, W. *Schwermineralanalyse* (Ferdinand Enke Verlag, Stuttgart, 1983).

19. Straus, L. G. A mosaic of change: the Middle–Upper Paleolithic transition as viewed from New Mexico and Iberia. *Quat Int.* **137**, 47-67 (2005).

20. Zilhão, J. Chronostratigraphy of the Middle-to-Upper Paleolithic Transition in the Iberian Peninsula. *Pyrenae* **37**, 7–84 (2006).

21. Zilhão, J. *et al.* Pego do Diabo (Loures, Portugal): Dating the emergence of anatomical modernity in westernmost Eurasia.*PLoS ONE* **5**, e8880 (2010).

22. Straus, L. G., Bicho, N. & Winegardner, A. C. The Upper Palaeolithic settlement of Iberia: First-generation maps. *Antiquity* **74**, 553–66 (2000).

23. Cacho, C. *et al.* El Paleolítico superior en el interior de la Península Ibérica. Revisión crítica y perspectivas de futuro. El Paleolítico Superior Peninsular. *Novedades del Siglo XXI, Barcelona*, 115-136 (2010).

24. Sánchez-Yustos, P. & Díez, F. Dancing to the rhythms of the Pleistocene? Early Middle Paleolithic population dynamics in NW Iberia (Duero Basin and Cantabrian Region). *Quat. Sci. Rev.* **121**, 75-88 (2015).

25. Jordá, J. in *Cuaternario y Arqueología. Homenaje a Francisco Giles Pacheco* (ed Mata, E.) 101-110 (Diputación Provincial de Cádiz, Cádiz, 2010).

26. Wood, R. E. *et al.* Radiocarbon dating casts doubt on the late chronology of the Middle to Upper Paleolithic transition in southern Iberia. *PNAS* **110**, 2781-2786 (2013).

27. Higham, T. *et al.* The timing and spatiotemporal patterning of Neanderthal disappearance. *Nature* **512**, 306–309 (2014).

28. Kehl, M. *et al.* Late Neanderthals at Jarama VI (central Iberia)? *Quat. Res.* **80**, 218-234 (2013).

29. Álvarez-Alonso, D., Andrés-Herrero, M., Díez-Herrero, A., Medialdea, A. & Rojo-Hernández, J. Neanderthal settlement in central Iberia: Geo-archaeological research in the Abrigo del Molino site, MIS 3 (Segovia, Iberian Peninsula). http://www.sciencedirect.com/science/article/pii/S104061821530094X (2016).

30. Kehl, M. *et al.* Dating the last Neanderthals in Central Iberia - New evidence from Abrigo del Molino, Segovia, Spain. *Geophysical Research Abstracts* **19**, 6402 (2017).

31. Alcaraz-Castaño, M. *et al.* A context for the last Neandertals of interior Iberia: Los Casares cave revisited. *PLoS ONE* **12(7)**, e0180823 (2017).

32. Navazo, M., Díez, C., Torres, T., Colina, A. & Ortiz, J. E. *La cueva de Prado Vargas. Un yacimiento del Paleolítico Medio en el sur de la Cordillera Cantábrica* (Museo de Altamira, Monografías 20, 2005), pp. 151-166.

33. Arnold, L., Demuro, M., Navazo, M., Benito-Calvo, A. & Pérez-González, A. OSL dating of the Middle Palaeolithic Hotel California site, Sierra de Atapuerca, north-central Spain. *Boreas* **42**, 285-305 (2013).

34. Díez, C., Jordá, J. & Arceredillo, D. in *Los cazadors recolectores del Pleisteceno y del Holoeno en Iberia y el Estrecho de Gibraltar* (ed Sala Ramos, R.) 608-610 (Universidad de Burgos-Fundación Atapuerca, Burgos, 2014).

35. Díez, C. *et al.* El Paleolítico Medio en el valle del Arlanza (Burgos). Los sitios de La Ermita, Millán y La Mina. *Cuaternario & Geomorfología* **22**, 135-157 (2008).

36. Navazo, M. & Carbonell, E. Neanderthal settlement patterns during MIS 4-3 in Sierra de Atapuerca (Burgos, Spain). *Quat. Int.* **331**, 267-277 (2014).

37. Silva, P. G. *et al.* Contexto geomorfológico y principales rasgos tecnológicos de nuevos yacimientos del Pleistoceno Medio y Superior en el Valle Inferior del Manzanares (Madrid, España). *Estudios Geológicos* **68**, 57-89 (2012).

38. Baena-Preysler, J., Ortiz, I., Torres, C. & Bárez, S. Recycling in abundance: Re-use and recycling processes in the Lower and Middle Paleolithic contexts of the central Iberian Peninsula. *Quat. Int.* **361**, 142-154 (2015).

39. Alcaraz-Castaño, M. Central Iberia around the Last Glacial Maximum: Hopes and Prospects. *Journal of Anthropological Research* **71**, 565-578 (2015).

40. Valladas, H. *et al.* TL dating of Upper Palaeolithic sites in the Côa Valley (Portugal). *Quat. Sci. Rev.* **20**, 939–943 (2001).

41. Aubry, T., Luis, L., Mangado Llach, J. & Matías, H. We will be known by the tracks we leave behind: Exotic lithic raw materials, mobility and social networking among the Côa Valley foragers (Portugal). *Journal of Anthropological Archaeology* **31**, 528-550 (2012).

42. Gaspar, R., Ferreira, J., Carrondo, J., Silva, M. J. & García-Vadillo, F. J. Open-air Gravettian lithic assemblages from Northeast Portugal: The Foz do Medal site (Sabor valley). *Quat. Int.* **406**, 44-64 (2016).

43. Mosquera, M. *et al.* Valle de las Orquídeas: un yacimiento al aire libre del Pleistoceno Superior en la Sierra de Atapuerca (Burgos). *Trabajos de Prehistoria* **64**, 143-155 (2007).

44. Carretero, J. M. *et al.* A Late Pleistocene-Early Holocene archaeological sequence of Portalón de Cueva Mayor (Sierra de Atapuerca, Burgos, Spain). *Munibe (Antropologia-Arkeologia)* **59**, 67-80 (2008).

45. de la Peña, P. The beginning of the Upper Paleolithic in the Baetic Mountain area (Spain). *Quat Int.* **318**, 69-89 (2013).

46. Aubry, T., Luis, L., Mangado Llach, J. & Matias, H. Adaptation to Resources and Environments during the Last Glacial Maximum by Hunter-Gatherer Societies in Atlantic Europe. *Journal of Anthropological Research* **71**, 521-544 (2015).

47. Aubry, T. *et al.* Upper Palaeolithic lithic raw material sourcing in Central and Northern Portugal as an aid to reconstructing hunter-gatherer societies. *Journal of Lithic Studies* **3** (2016), p. 22.

48. Alcaraz-Castaño, M. *et al.* Los orígenes del Solutrense y la ocupación pleniglaciar del interior de la Península Ibérica: implicaciones del nivel 3 de Peña Capón (valle del Sorbe, Guadalajara). *Trabajos de Prehistoria* **70**, 28-53 (2013).

49. Alcaraz-Castaño, M. *et al.* The human settlement of Central Iberia during MIS 2: New technological, chronological and environmental data from the Solutrean workshop of Las Delicias (Manzanares River valley, Spain). *Quat. Int.* **431**, 104-124 (2017).

50. Alcolea-González, J. J. & de Balbín-Behrmann, R. in *Arte sin artistas, una mirada al Paleolítico* (Museo Arqueológico Regional, Comunidad de Madrid, Madrid, 2013), pp. 187-207.

51. Arsuaga, J. L. *et al.* Neandertals at Atapuerca: the MIS3 Galería de las Estatuas site. *Proceedings of the European Society for the study of Human Evolution* **5**, pp. 36 (2016).

52. Alcaraz-Castaño, M. El Paleolítico Superior pre-Magdaleniense en el centro de la Península Ibérica: hacia un nuevo modelo. *ARPI (Arqueología y Prehistoria del interior peninsular)* **04**, 34-48 (2016).

53. Alcaraz-Castaño, M. *et al.* Neandertal adaptations in Central Iberia: a multi-proxy investigation of the Middle Paleolithic site of Peña Cabra, Guadalajara, Spain. *Proceedings of the European Society for the study of Human Evolution* **5**, pp. 31 (2016).

54. Baquedano, E. *et al.* The Des-Cubierta Cave (Pinilla del Valle, Comunidad de Madrid, Spain): a Neanderthal site with a likely funerary/ritualistic connection. *Proceedings of the European Society for the study of Human Evolution* **5**, pp. 41 (2016).

55. Klasen, N. *et al.* Application of luminescence dating of archaeological sequences – examples from Europe and North Africa. *Geophysical Research Abstracts* **19**, 6919 (2017).

56. Buggle, B., Glaser, B., Hambach, U., Gerasimenko, N. & Marković, S. An evaluation of geochemical weathering indices in loess–paleosol studies. *Quat. Int.* **240**, 12-21 (2011).

57. Mauz, B. & Lang, A. Removal of the feldspar-derived luminescence component from polymineral fine silt samples for optical dating applications: evaluation of chemical treatment protocols and quality control procedures. *Ancient TL* **22**, 1–8 (2004).

58. Durcan, J. A., King, G. E. & Duller, G. A. T. DRAC: Dose rate and age calculator for trapped charge dating. *Quaternary Geochronology* **28**, 54-61 (2015).

59. Guérin, G., Mercier, N. & Adamiec, G. Dose-rate conversion factors: update. *Ancient TL* **29**, 5–8 (2011).

60. Bronk Ramsey, C. & Lee, S. Recent and planned developments of the program OxCal. *Radiocarbon* **55**, 720–730 (2013).

61. Reimer, P. J. *et al. IntCal13 and Marine13 radiocarbon age calibration curves 0-50,000 years cal BP* (2013).

62. Silva, P. *et al.* Datos geoarqueológicos de la terraza compleja del Manzanares entre el sector del 12 de octubre y la desembocadura del arroyo Butarque (Villaverde, Madrid). *Cuaternario y Geomorfología* **22**, 47-70 (2008).

63. Navazo, M. & Díez, C. Prado Vargas y la variabilidad tecnológica a finales del Paleolítico Medio en la meseta norte. *Treballs d'Arqueologia* **14**, 121-139 (2008).

64. Navazo, M. *et al.* Hundidero: MIS 4 open air Neanderthal occupations in Sierra de Atapuerca. *Archaeology Ethnology & Anthropology of Eurasia* **39**, 29–41 (2011).

65. Baquedano, E., Márquez, B., Laplana, C., Arsuaga, J. L. & Pérez-González, A. in *Pleistocene and Holocene hunter-gatherers in Iberia and the Gibraltar Straits: the current archaeological record* (ed Sala Ramos, R.) 577-584 (Universidad de Burgos-Fundación Atapuerca, Burgos, 2014).

66. Pérez-González, A., Rubio, S., Panera, J. & Uribelarrea, J. Geocronología de la sucesión arqueoestratigráfica de Los Estragales en la Terraza Compleja de Butarque (Valle del río Manzanares, Madrid). *Geogaceta* **45**, 39-42 (2008).

67. Pérez-González, A. *et al.* in *Actas de la 1ª Reunión de científicos sobre cubiles de hiena (y otros grandes carnívoros en los yacimientos arqueológicos de la Península Ibérica)* (eds Baquedano, E. & Rosell, J.) 404-419 (Zona Arqueológica 13, 2010).

68. Terradillos-Bernal, M. *et al.* San Quirce (Palencia, Spain). A Neanderthal open air campsite with short term-occupation patterns. *Quat. Int.* **435**, 115-128 (2017).

69. Domínguez Alonso, R. M., Fernández, S., Ruiz, B. & Gil, M. J. "Nuevos datos sobre la Terraza Compleja de Butarque en Villaverde Bajo" (IV Jornadas sobre Patrimonio Arqueológico de la Comunidad de Madrid, 2009), pp. 339-43.

70. Laplana, C. *et al.* Biocronología de la Terraza Compleja de Butarque del río Manzanares en el Estanque de Tormentas al sur de Madrid (España). *Estudios Geológicos* **71**, e028 (2015).

71. Panera, J. *et al.* Manzanares Valley (Madrid, Spain): A good country for Proboscideans and Neanderthals. *Quat. Int.* **318**, 326-343 (2014).

72. Arsuaga, J. L. *et al.* Understanding the ancient habitats of the last-interglacial (late MIS 5) Neanderthals of central Iberia: Paleoenvironmental and taphonomic evidence from the Cueva del Camino (Spain) site. *Quat. Int.* **275**, 55-75 (2012).

73. Silva, P. G. *et al.* Stratigraphy of the Arriaga Palaeolithic sites. Implications for the geomorphological evolution recorded by thickened fluvial sequences within the Manzanares River valley (Madrid Neogene Basin, Central Spain). *Geomorphology* **196**, 138–161 (2013).

74. Díez-Martín, F. *et al.* La ocupación neandertal en el Cañón de La Horadada (Mave, Palencia, España): Nuevas perspectivas arqueológicas en Cueva Corazón. *Munibe* **62**, 65-85 (2011).

75. Mercier, N. *et al.* Fariseu: First confirmed open-air Palaeolithic parietal art site in the Côa Valley (Portugal). *Antiquity* **80** (310), Project Gallery [online series], http://antiquity.ac.uk/projgall/mercier (2006).

76. Corchón, M. S. *et al.* Datación de las pinturas y revisión del Arte Paleolítico de Cueva Palomera Ojo Guareña, Burgos, Spain), *Zephyrus* **49**, 37-60 (1996).

77. Cacho, C. *et al.* Human landscapes of the Late Glacial Period in the interior of the Iberian Peninsula: La Peña de Estebanvela (Segovia, Spain). *Quat. Int.* **272–273**, 42–54 (2012).

78. Utrilla, P. *et al.* The Ebro Basin in NE Spain: A crossroads during the Magdalenian. *Quat. Int.* **272-273**, 88-104 (2012).

79. Rasilla, M., Hoyos, M. & Cañaveras, J. C. El Abrigo de Verdelpino (Cuenca). Revisión de su evolución sedimentaria y arqueológica. *Complutum Extra* **6-I**, 75-82 (1996).

80. Vega-Toscano, L. G. *et al.* "Nuevas investigaciones sobre los yacimientos paleolíticos en la Sierra Norte de la Comunidad de Madrid" (Actas de las V Jornadas de Patrimonio Arqueológico en la Comunidad de Madrid. Los primeros pobladores: Arqueología del Pleistoceno, 2010), pp. 115-132.

81. Torre, I. *et al.* Chronological and palaeoenvironmental context of human occupations at the Buendía rockshelter (Central Spain) during the late Upper Pleistocene in inland Iberia. *J. Quat. Sci.* **30**, 376-390 (2015).

82. Tapias, F. *et al.* Geoarqueología y paleontología de los depósitos de Pleistoceno Superior del antiguo arroyo Abroñigal (Cuenca del Manzanares, Madrid): el yacimiento del Puente de los Tres Ojos. *Cuaternario y Geomorfología* **26**, 105–32 (2012).

83. Canals, A. *et al.* in *El Paleolítico superior peninsular: Novedades del siglo XXI* (ed Mangado, X.) 199-218 (Monografi es del Seminari d'Estudis i Recerques Prehistòriques 88, Universitat de Barcelona, 2010).

84. Zilhão, J. *et al.* Precise dating of the Middle-to-Upper Paleolithic transition in Murcia (Spain) supports late Neandertal persistence in Iberia. *Heliyon* **3**, e00435 (2017).

85. Murray, A. S. & Wintle, A. G. Luminescence dating of quartz using an improved single aliquot regenerative dose protocol. *Radiation Measurements* **32**, 57-73 (2000).

86. Murray, A. S. & Wintle, A. G. The single aliquot regenerative dose protocol: potential for improvements in reliability. *Radiation Measurements* **37**, 377-381 (2003).
